# Synthesis of novel sulphamethoxazole derivatives and exploration of their anticancer and antimicrobial properties

**DOI:** 10.1371/journal.pone.0283289

**Published:** 2023-03-23

**Authors:** Rita Vaickelionienė, Vilma Petrikaitė, Irena Vaškevičienė, Alvydas Pavilonis, Vytautas Mickevičius

**Affiliations:** 1 Department of Organic Chemistry, Kaunas University of Technology, Kaunas, Lithuania; 2 Laboratory of Drug Targets Histopathology, Institute of Cardiology, Lithuanian University of Health Sciences, Kaunas, Lithuania; 3 Institute of Biotechnology, Life Sciences Center, Vilnius University, Vilnius, Lithuania; 4 Lithuanian Energy Institute, Laboratory of Heat-Equipment Research and Testing, Kaunas, Lithuania; 5 Institute of Microbiology and Virology, Lithuanian University of Health Sciences, Kaunas, Lithuania; Virginia Commonwealth University, UNITED STATES

## Abstract

A series of new derivatives based on sulfamethoxazole were designed and synthesized in this study. The structures of the new compounds were confirmed based on a comprehensive characterization of spectral data by applied IR and ^1^H as well as ^13^C NMR spectroscopy. The prepared compounds were tested for their anticancer and antimicrobial properties. Hydrazone **16b** demonstrated convincing anticancer effect against all tested cell cultures such as human prostate carcinoma PPC-1 and human kidney carcinoma CaKi-1 cell lines, and human fibroblasts HF, n = 3. The most promising compound **16b** showed higher activity against CaKi-1 cell line than the anticancer drugs axitinib and pazopanib used to treat renal cancer. Also, it was more active in the PPC-1 cell line compared to the approved PARP inhibitor Olaparib. Hydrazone **16b** was also found to possess good antimicrobial properties against gram-positive bacteria strains of *Staphylococcus aureus*, *Staphylococcus epidermidis*, as well as *Bacillus cereus*.

## Introduction

Synthesis and investigation of heterocyclic compounds have been one of the most important goals of organic and medicinal chemistry due to their broad potential for biological properties. Numerous heterocycle core is rightly considered privileged because as statistics show, more than 85% of all biologically active compounds possess a heterocyclic moiety [1]. Among them, *O*, *N*-heterocyclic rings are distinguished not only due to the simple synthesis but also by their wide distribution and bioactivity. Isoxazole derivatives being an entity of this group of compounds make a significant contribution displaying a wide range of important therapeutic properties viz. anti-inflammatory, anti-ulcer [2], anti-cancer [3, 4], anti-tubulin [5], antibacterial [6, 7], antioxidant [8, 9], immunoregulatory [10], antiplatelet [11], antifungal [12], anticonvulsant [13] and many others [14–16]. The substitution and modification of isoxazole structure provided a large library of biologically active compounds useful for the development of therapeutic agents with higher potency and lower toxicity [17]. Many isoxazole derivatives have been proved to possess activities like cytotoxicity, HIV inhibition, anti-tuberculosis, antiprotozoal [18–22], as well as androgen antagonists’ action [23, 24].

**Fig 1:**
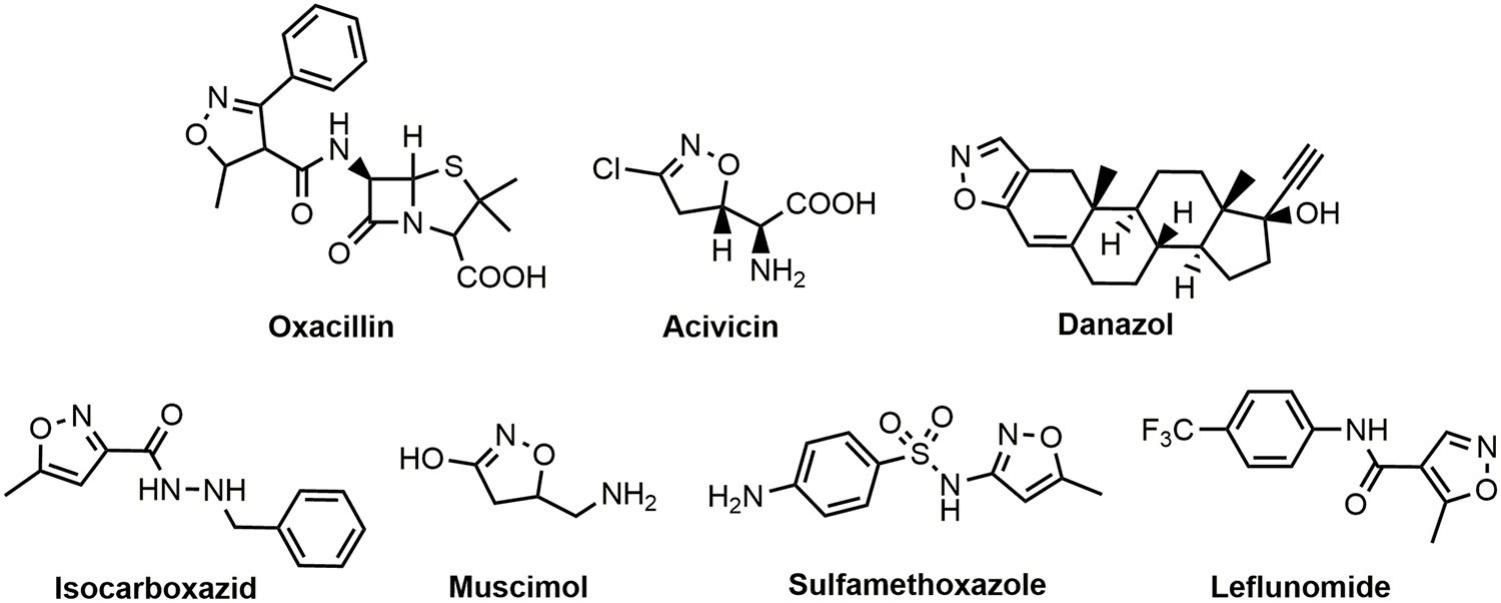
Isoxazole-based pharmaceuticals.

Medications with a structural isoxazole scaffold belong to different categories with diverse pharmaceutical action. For instance, sulfamethoxazole and oxacillin (Fig 1) are used as antibacterial, acivicin, a product of Streptomyces sviceus is effective as antitumor and antileishmanial agent, danazol is an androgen, isocarboxazid treats depression by restoring the balance of neurotransmitters. Leflunomide (Fig 1) has been approved for the management of active rheumatoid arthritis, muscimol is a potent and selective naturally derived agent, which displays sedative-hypnotic and depressant activity [25–27].

Sulfamethoxazole, a derivative with the isoxazole ring in the structure, is board spectrum bacteriostatic antibiotic frequently used in combination with trimethoprim. As a single medication, sulfamethoxazole is used to treat bacterial infections viz. bronchitis, urinary tract infections and prostatitis, also is effective against gram-positive *Staphylococcus aureus* and gram-negative *Escherichia coli* strains [28]. In recent years, a great number of sulfamethoxazole derivatives were synthesized, characterized and tested to obtain high-potency agents for clinical purposes. The investigations have disclosed analgesic, antioxidant, antimicrobial [29], antibacterial [30–33], and antifungal [32, 33] properties of newly synthesized derivatives. They proved to be promising starting points for the development of new antichlamydial medications [34] and HSP70 inhibitors [35]. These results also suggested that they are promising candidates for lung cancer treatment by targeting EGFR tyrosine kinase T790M/L858R [36]. Sulfonamide-salicylaldehyde imines were investigated against methicillin- and trimethoprim/sulfamethoxazole (SMX)-resistant Gram-positive species. *Staphylococci* exhibited the highest susceptibility including resistant strains at MIC values from 3.91 μM [37]. In addition, complexes of sulfonamide core-based ligands with the transition metals (V, Fe, Co, Ni, Cu, and Zn) when evaluated against four bacterial (*Staphylococcus aureus*, *Bacillus subtilis*, *Escherichia coli*, and *Klebsiella pneumoniae*) and two fungal (*Aspergillus flavus* and *Aspergillus niger*) species showed enhanced bioactions in comparison with their respective precursors. It is noteworthy, that the same complexes gave excellent results in antioxidant and enzyme inhibition tests implying their bioactive nature [38]. Sulfonamides complexed with metals have proven as a useful and promising tool to reduce microbial adhesion on inert surfaces, stimulating to improve the insertion of compounds as new antibacterial coating agents for medicinal and hospital materials. Sulfonamide-metal complexes have proven to be useful and promising tools in reducing microbial adhesion to inert surfaces and in promoting the development of new antibacterial coatings for medical and hospital materials. Sulfonamide-Au, Cd, Ag, Cu, and Hg complexes demonstrated excellent activity against various microorganisms and confirmed the effectiveness of sulfonamide-metal complexes against rapidly growing mycobacteria (*Mycobacterium abscessus*, *M*. *fortuitum*, and *M*. *massiliense)* biofilm [39].

In the light of the above facts and due to the huge development in antimicrobial activities of sulfamethoxazole derivatives, as well as in the continuation of our works in the field of the synthesis and investigation of various sulfonamides [40–45] we have synthesized a series of completely new unpublished sulfamethoxazole-based derivatives and have screened them against cancer cell and several standard bacteria and fungus to study their potential in search of novel anticancer and antimicrobial compounds.

## Materials and methods

### Synthesis

Starting materials and solvents were purchased from Sigma-Aldrich (St. Louis, MO, USA) and used without further purification. The reaction course and purity of the synthesized compounds were monitored by TLC using aluminium plates pre-coated with Silica gel with F254 nm (Merck KGaA, Darmstadt, Germany). Melting points were determined with a B-540 melting point analyser (Büchi Corporation, New Castle, DE, USA) and are uncorrected. IR spectra (ν, cm^–1^) were recorded on a Perkin–Elmer Spectrum 100 FT–IR spectrometer (Perkin–Elmer Inc., Waltham, MA, USA) using KBr pellets. NMR spectra were recorded on a Brucker Avance III (400, 101 MHz) spectrometer (Bruker BioSpin AG, Fällanden, Switzerland). Chemical shifts were reported in (*δ*) ppm relative to tetramethylsilane (TMS) with the residual solvent as internal reference ([D6] DMSO, *δ* = 2.50 ppm for ^1^H and *δ* = 39.52 ppm for ^13^C). Data are reported as follows: chemical shift, multiplicity, coupling constant [Hz], integration and assignment. Mass spectra were obtained on Bruker Daltonic–maXis 4G mass spectrometer (Bruker Daltonics, Bremen, Germany) with ESI ionization. Elemental analyses (C, H, N) were conducted using the Elemental Analyzer CE-440, their results were found to be in good agreement (±0.3%) with the calculated values.

3-((4-(N-(5-methylisoxazol-3-yl)sulfamoyl)phenyl)amino)propanoic acid **(2)**

A mixture of aromatic amine **1** (75 mmol, 19 g), acrylic acid (225 mmol, 15.5 mL) and water 180 mL was heated at reflux for 24 h, then cooled down, the formed precipitate was filtered off, washed with water and purified by dissolving it in 7.5% sodium solution (90 mL), filtering and acidifying the filtrate with acetic acid to pH 6.

White solid, yield 23.1 g, 94.8%, m.p. 188–189°C.

^1^H-NMR (400 MHz, DMSO-d_6_): δ = 2.28 (s, 3H, CH_3_), 2.45–2.49 (m, overlaps with the signal of the DMDO-d_6_, 2H, CH_2_CO), 3.28 (dd, J = 10.9, 5.7 Hz, 2H, NHCH_2_), 6.09 (s, 1H, CH_isox_), 6.62 (d, J = 8.7 Hz, 2H, H_ar_), 6.62–6.75 (m, 1H, NHCH_2_), 7.52 (d, J = 8.7 Hz, 2H, H_ar_), 11.48 (br. s, 2H, NH, OH) ppm;

^13^C-NMR (101 MHz, DMSO-d_6_): δ = 12.07, 33.38, 38.21, 95.31, 110.93, 124.38, 128.75, 152.28, 157.98, 169.91, 172.93 ppm.

IR (KBr): ν_max_ = 3398, 3289 (2NH), 1709 (C = O), 1599 (C = N) cm^–1^.

Calcd for C_13_H_15_N_3_O_5_S, %: C 47.99; H 4.65; N 12.92. Found, %: C 47.76; H 4.93; N 12.82.

Methyl 3-((4-(N-(5-methylisoxazol-3-yl)sulfamoyl)phenyl)amino)propanoate **(3)**

To a mixture of acid **2** (50 mmol, 16.3 g) and methanol (75 mL) catalytic amount of sulfuric acid (5 drops) was added and the mixture was refluxed for 26 h, then cooled down, and the formed precipitate was filtered off, boiled with 5% sodium carbonate solution (50 mL), cooled down, filtered of, washed with plenty of water and recrystallized from methanol.

White solid, yield 12.9 g, 75.8%, m.p. 148–149°C.

^1^H-NMR (400 MHz, DMSO-d_6_): δ = 2.28 (s, 3H, CH_3_), 2.58 (t, J = 6.6 Hz, 2H, CH_2_CO), 3.28–3.33 (m, 2H, NHCH_2_), 3.60 (s, 3H, OCH_3_), 6.09 (s, 1H, CH_isox_), 6.62 (d, J = 8.7 Hz, 2H, H_ar_), 6.69 (t, J = 5.4 Hz, 1H, NHCH_2_), 7.52 (d, J = 8.7 Hz, 2H, H_ar_), 10.95 (s, 1H, NH) ppm;

^13^C-NMR (101 MHz, DMSO-d_6_): δ = 12.04, 33.09, 38.06, 95.28, 110.96, 124.46, 128.73, 152.16, 157.91, 169.91, 171.81 ppm.

IR (KBr): ν_max_ = 3378, 3170 (NH), 1721 (C = O), 1619, 1602 (C = N) cm^–1^.

Calcd for C_14_H_17_N_3_O_5_S, %: C 49.55; H 5.05; N 12.38. Found, %: C 49.41; H 5.20; N 12.42.

4-((3-Hydrazineyl-3-oxopropyl)amino)-N-(5-methylisoxazol-3-yl)benzenesulfonamide **(4)**

A mixture of ester **3** (30 mmol, 10.31 g), hydrazine monohydrate (90 mmol, 4.5 g) and propan-2-ol (150 mL) was heated at reflux for 24 h, then cooled down. The solvent was decanted, and the viscous mass was twice washed with boiling 2-propanol. The resulting sufficiently pure material without addition purification was used for further syntheses.

Brown viscous mass, yield 9.06 g, 89%.

^1^H-NMR (400 MHz, DMSO-d_6_): δ = 2.15 (s, 3H, CH_3_), 2.28 (t, J = 7.0 Hz, 2H, CH_2_CO), 3.25 (q, J = 6.5 Hz, 2H, NHCH_2_), 4.13 (br. s, 3H, NHNH_2_, NH), 5.85 (s, 1H, CH_isox_), 6.12 (t, J = 5.2 Hz, 1H, CH_isox_), 6.51 (d, J = 8.4 Hz, 2H, H_ar_), 7.42 (d, J = 8.4 Hz, 2H, H_ar_), 9.03 (br. s, 1H, NH) ppm.

^13^C-NMR (101 MHz, DMSO-d_6_): δ = 12.12, 33.32, 40.19, 96.37, 110.63, 127.81, 131.92, 150.24, 155.75, 169.88, 170.77 ppm.

Procedures for the preparation of compounds **5, 6**

Method A: A mixture of carboxylic acid **2** (10 mmol, 3.25 g), carbamide (30 mmol, 1.8 g, **5**) or potassium thiocyanate (30 mmol, 4.08 g, **6**) and acetic acid (25 mL) was heated at reflux for 26 h, then hydrochloric acid was added (to pH 1–2) and the mixture was refluxed for 1 h. After completion of the reaction (TLC), the mixture was cooled down and diluted with water. The formed precipitate was filtered off, washed with water and recrystalized from methanol.

Method B for the synthesis of **6**: A mixture of thioureido acid **6a** (10 mmol, 3.84 g) and 15% hydrochloric acid (15 mL) was refluxed for 1 h, then cooled down and diluted with water, the formed precipitate was filtered off, washed with water and recrystalized from methanol to give a white solid with the yield of 3.44 g, 94%.

4-(2,4-Dioxotetrahydropyrimidin-1(2H)-yl)-N-(5-methylisoxazol-3-yl)benzenesulfonamide **(5)**

White solid, yield 2.59 g, 74%, m.p. 182–183°C.

^1^H-NMR (400 MHz, DMSO-d_6_): δ = 2.30 (s, 3H, CH_3_), 2.71 (t, J = 6.5 Hz, 2H, CH_2_CO), 3.86 (t, J = 6.5 Hz, 2H, NHCH_2_), 6.16 (s, 1H, CH_isox_), 7.56 (d, J = 8.4 Hz, 2H, H_ar_), 7.85 (d, J = 8.4 Hz, 2H, H_ar_), 10.54 (s, 1H, NH), 11.45 (s, 1H, NH) ppm;

^13^C-NMR (101 MHz, DMSO-d_6_): δ = 12.09, 30.99, 43.95, 95.40, 124.82, 127.25, 135.47, 146.12, 151.97, 157.48, 170.41, 170.42 ppm.

IR (KBr): ν_max_ = 3374, 3172 (2NH), 1729, 1683 (2C = O), 1592 (C = N) cm^–1^.

Calcd for C_14_H_14_N_4_O_5_S, %: C 48.00; H 4.03; N 15.99. Found, %: C 47.99; H 4.09; N 15.92.

N-(5-methylisoxazol-3-yl)-4-(4-oxo-2-thioxotetrahydropyrimidin-1(2H)-yl)benzenesulfonamide **(6)**

Light brown solid, yield 2.98 g, 81.4%, m.p. 240–241°C.

^1^H-NMR (400 MHz, DMSO-d_6_): δ = 2.31 (s, 3H, CH_3_), 2.81 (t, J = 6.8 Hz, 2H, CH_2_CO), 3.94 (t, J = 6.8 Hz, 2H, NHCH_2_), 6.19 (s, 1H, CH_isox_), 7.61 (d, J = 8.2 Hz, 2H, H_ar_), 7.91 (d, J = 8.2 Hz, 2H, H_ar_), 11.41 (s, 1H, NH), 11.55 (br. s, 1H, NH) ppm;

^13^C-NMR (101 MHz, DMSO-d_6_): δ = 12.12, 30.36, 48.43, 95.48, 127.66, 128.36, 138.19, 148.91, 157.47, 166.97, 170.48, 179.62 ppm.

IR (KBr): ν_max_ = 3164, 3093 (NHNH2), 1732 (C = O), 1593 (C = N) cm^–1^.

Calcd for C_14_H_14_N_4_O_4_S_2_, %: C 45.89; H 3.85; N 15.29. Found, %: C 45.97; H 3.86; N 15.22.

3-(1-(4-(N-(5-methylisoxazol-3-yl)sulfamoyl)phenyl)thioureido)propanoic acid **(6a)**

A mixture of compound **6** (1.36 mmol, 0.5 g) and aqueous 5% sodium hydroxide solution (5 mL) was kept at room temperature for 24 h, then filtered off, and the filtrate was acidified with glacial acetic acid to pH 6–7. The formed solid was filtered off, washed with hexane, dried and purified by dissolving it in 5% sodium carbonate, filtering and acidifying the filtrate with acetic acid to pH 6–7.

White solid, 0.5 g, 95.7%, m.p. 182–183°C.

^1^H-NMR (400 MHz, DMSO-d_6_): δ = 2.54 (t, J = 7.8 Hz, 2H, CH_2_CO), 4.14 (t, J = 7.8 Hz, 2H, NHCH_2_), 6.18 (s, 1H, CH_isox_), 7.48 (d, J = 8.2 Hz, 2H, H_ar_), 7.88 (d, J = 8.2 Hz, 2H, H_ar_), 11.64 (br. s, 1H, NH), 12.24 (br. s, 1H, OH) ppm;

^13^C-NMR (101 MHz, DMSO-d_6_): δ = 12.13, 32.09, 49.94, 95.46, 128.27, 128.95, 138.49, 146.99, 157.55, 170.33, 172.32, 181.93 ppm.

IR (KBr): ν_max_ = 3451, 3291, 3174 (NH, NH_2_), 1710 (C = O), 1618 (C = N) cm^–1^.

MS, m/z [M-H]+ calcd for C_14_H_16_N_4_O_5_S_2_ 383.05, found 383.0.

Calcd for C_14_H_16_N_4_O_5_S_2_, %: C 43.74; H 4.20; N 14.57. Found, %: C 43.69; H 4.25; N 14.64.

3-((4-(N-(5-methylisoxazol-3-yl)sulfamoyl)phenyl)amino)-N-(4-sulfamoylphenyl)propenamide **(7)**

To a mixture of acid **2** (5 mmol, 1.63 g), 4-aminobenzenesulfonamide (6 mmol, 1.03 g) and DMSO (10 mL) triethyl amine (19 mmol, 2.7 mL, dropwise) and HBTU (6.3 mmol, 2.4 g) were added and the mixture was stirred for 20 h at room temperature. After completion of the reaction (TLC) the mixture was diluted with water (20 mL), the formed precipitate was filtered off, washed with water and recrystallized from methanol.

White solid, yield 1.64 g, 68.6%, m.p. 213–214°C.

^1^H-NMR (400 MHz, DMSO-d_6_): δ = 2.29 (s, 3H, CH_3_), 2.63 (t, J = 6.7 Hz, 2H, CH_2_CO), 3.41 (t, J = 6.4 Hz, 2H, NHCH_2_), 6.10 (s, 1H, CH_isox_), 6.66 (d, J = 8.6 Hz, 2H, H_ar_), 6.75 (t, J = 5.8 Hz, 1H, NH), 7.24 (s, 2H, NH2), 7.54 (d, J = 8.5 Hz, 2H, H_ar_), 7.75 (s, 4H, H_ar_), 10.29 (s, 1H, NH), 11.96 (s, 1H, NH) ppm;

^13^C-NMR (101 MHz, DMSO-d_6_): δ = 12.07, 35.81, 38.27, 95.31, 110.97, 118.58, 124.35, 126.69, 128.75, 138.21, 142.02, 152.29, 157.95, 169.90, 170.00 ppm.

IR (KBr): ν_max_ = 3361, 3317, 3272 (NH), 1678 (C = O), 1595 (C = N) cm^–1^.

Calcd for C_19_H_21_N_5_O_6_S_2_, %: C 47.59; H 4.41; N 14.61. Found, %: C 47.65; H 4.45; N 14.68.

N-(2,5-dimethyl-1H-pyrrol-1-yl)-3-((4-(N-(5-methylisoxazol-3-yl)sulfamoyl)phenyl)amino)propenamide **(8)**

To a solution of hydrazide **4** (1.5 mmol, 0.51 g) in 1,4-dioxane (10 mL) hexane-2,5-dione (4.5 mmol, 0.51 g) and catalytic amount of acetic acid (2 drops) were added and the mixture was heated at reflux for 1 h, the volatile fraction was evaporated under reduced pressure, and the residue was dissolved in methanol, and the obtained solution was diluted with water. The formed precipitate was filtered off, washed with methanol and recrystallized from propan-2-ol.

Light brown solid, yield 0.18 g, 28%, m.p. 118–119°C.

^1^H-NMR (400 MHz, DMSO-d_6_): δ = 1.92, 1.94 (2s, 6H, 2CH_3_), 2.28 (s, 3H, CH_3_), 2.54 (t, J = 6.7 Hz, 2H, CH_2_CO), 3.43 (q, J = 6.5 Hz, 2H, NHCH_2_), 5.61, 5.66 (2s, 2H, 2CH_pyr_), 6.10 (s, 1H, CH _isox_), 6.67 (t, J = 8.6 Hz, 2H, H_ar_), 6.77 (t, J = 5.6 Hz, 1H, NH), 7.54 (d, J = 8.6 Hz, 2H, H_ar_), 10.61 (s, 1H, NH), 10.96 (br. s, 1H, NH) ppm;

^13^C-NMR (101 MHz, DMSO-d_6_): δ = 10.90, 12.06, 32.86, 38.43, 95.32, 102.88, 111.00, 124.54, 126.73, 128.74, 152.16, 158.01, 169.85, 170.00 ppm.

IR (KBr): ν_max_ = 3582, 3381, 3259, 3159 (NH), 1685 (C = O), 1599 (C = N) cm^–1^.

Calcd for C_19_H_23_N_5_O_4_S, %: C 54.66; H 5.55; N 16.78. Found, %: C 54.89; H 5.73; N 16.72.

4-((3-(3,5-Dimethyl-1H-pyrazol-1-yl)-3-oxopropyl)amino)-N-(5-methylisoxazol-3-yl)benzenesulfonamide **(9)**

To a solution of hydrazide **4** (1.5 mmol, 0.51 g) in 1,4-dioxane (10 mL) pentane-2,4-dione (4.5 mmol, 0.45 g) and catalytic amount of hydrochloric acid (1 drop) were added and the mixture was heated at reflux for 1 h, the volatile fraction was evaporated under reduced pressure, and the residue was poured with boiling water, stirred and left to cool down to room temperature. The formed precipitate was filtered off, washed with 2-propanol and recrystallized from propan-2-ol.

Light brown solid, yield 0.43 g, 71.2%, m.p. 144–145°C.

^1^H-NMR (400 MHz, DMSO-d_6_): δ = 2.16, 2.46 (2s, 6H, 2CH_3_), 2.28 (s, 3H, CH_3_), 3.28–3.34 (m, 2H, CH_2_CO), 3.38–3.48 (m, 2H, NHCH_2_), 6.10 (s, 1H, CH_isox_), 6.17 (s, 1H, CH_pyr_), 6.65 (d, J = 8.5 Hz, 2H, H_ar_), 6.77 (t, J = 5.6 Hz, 1H, NH), 7.53 (d, J = 8.5 Hz, 2H, H_ar_), 10.96 (s, 1H, NH) ppm;

^13^C-NMR (101 MHz, DMSO-d_6_): δ = 12.05, 13.45, 14.08, 34.47, 37.60, 95.28, 110.93, 111.19, 124.37, 128.74, 143.24, 151.45, 152.23, 157.92, 169.88, 171.62 ppm.

IR (KBr): ν_max_ = 3674, 3410 (NH), 1716 (C = O), 1596 (C = N) cm^–1^.

Calcd for C_18_H_21_N_5_O_4_S, %: C 53.59; H 5.25; N 17.36. Found, %: C 53.31; H 5.33; N 17.04.

General procedure for the preparation carbo(thio)amides **10, 11**

To a solution of hydrazide **4** (3 mmol, 1.02 g) in methanol (30 mL) phenyl isocyanate or isothiocyanate (6 mmol) was added dropwise, and the mixture was heated at reflux for 24 h, then volatile fraction was evaporated under reduced pressure, and the residue was dissolved in methanol, and the obtained solution was diluted with water. The formed precipitate was filtered off, washed with methanol and recrystallized from propan-2-ol.

2-(3-((4-(N-(5-methylisoxazol-3-yl)sulfamoyl)phenyl)amino)propanoyl)-N-phenylhydrazine-1-carboxamide **(10)**

White solid, yield 0.39 g, 28%, m.p. 204–205°C.

^1^H-NMR (400 MHz, DMSO-d_6_): δ = 2.28 (s, 3H, CH_3_), 2.44 (t, J = 7.0 Hz, 2H, CH_2_CO), 3.31–3.38 (m, 2H, NHCH_2_), 6.10 (s, 1H, CH_isox_), 6.65 (d, J = 8.9 Hz, 2H, H_ar_), 6.67 (t, J = 4.8 Hz, 1H, NH), 6.95 (t, J = 7.3 Hz, 1H, H_ar_), 7.25 (t, J = 7.7 Hz, 2H, H_ar_), 7.43 (d, J = 8.0 Hz, 2H, H_ar_), 7.54 (d, J = 8.6 Hz, 2H, H_ar_), 8.03, 8.36 (2s, 1H, NH), 8.70, 8.90, 9.03 (3s, 1H, NH), 9.74 (s, 1H, NH), 10.93 (br. s, 1H, NH) ppm;

^13^C-NMR (101 MHz, DMSO-d_6_): δ = 12.08, 32.81, 38.48, 95.30, 110.99, 118.50, 121.92, 124.43, 128.65, 128.74, 139.56, 152.25, 155.34, 157.95, 169.89, 170.42 ppm.

IR (KBr): ν_max_ = 3625, 3378, 3312, 3250 (NH), 1686, 1656 (C = O), 1598 (C = N) cm^–1^.

Calcd for C_20_H_22_N_6_O_5_S, %: C 52.39; H 4.84; N 18.33 Found, %: C 52.44; H 4.85; N 18.28.

2-(3-((4-(N-(5-methylisoxazol-3-yl)sulfamoyl)phenyl)amino)propanoyl)-N-phenylhydrazine-1-carbothioamide **(11)**

White solid, yield 0.46 g, 36.5%, m.p. 183–184°C.

^1^H-NMR (400 MHz, DMSO-d_6_): δ = 2.28 (s, 3H, CH_3_), 2.48 (t, 2H, CH_2_CO overlaps with the signal of the DMSO-d_6_), 3.32–3.38 (m, 2H, NHCH_2_), 6.10 (s, 1H, CH_isox_), 6.64 (d, J = 8.5 Hz, 2H, H_ar_), 6.64–6.72 (m, 1H, H_ar_, NH), 7.17 (t, J = 7.4 Hz, 1H, H_ar_), 7.33 (t, J = 7.6 Hz, 2H, H_ar_), 7.36–7.50 (m, 2H, H_ar_), 7.54 (d, J = 8.5 Hz, 2H, H_ar_), 9.04, 9.35, 9.55, 9.85, 9.95, 9.99 (6s, 3H, 3NH), 10.92 (br. s, 1H, NH) ppm;

^13^C-NMR (101 MHz, DMSO-d_6_): δ = 12.08, 32.85, 38.28, 95.32, 110.99, 116.80, 121.04, 124.43, 125.19, 125.88, 128.11, 128.76, 139.07, 152.26, 157.94, 169.91, 170.39, 187.69 ppm;

IR (KBr): ν_max_ = 3646, 3349, 3293, 3251 (NH), 1698 (C = O), 1594 (C = N) cm^–1^.

Calcd for C_20_H_22_N_6_O_4_S_2_, %: C 50.62; H 4.67; N 17.71. Found, %: C 50.59; H 4.65; N 17.75.

General procedure for the preparation of triazoles **12, 13**

A mixture of compounds **10** or **11** (2 mmol) in aqueous 4% sodium solution (10 mL) was heated at reflux for 4 h, then cooled down and acidified with diluted hydrochloric acid (1:1) to pH 2. The formed precipitate was filtered off, washed with water and recrystallized from methanol.

N-(5-methylisoxazol-3-yl)-4-((2-(5-oxo-4-phenyl-4,5-dihydro-1H-1,2,4-triazol-3-yl)ethyl)amino)-benzenesulfonamide **(12)**

White solid, yield 0.83 g, 94%, m.p. 188–189°C.

^1^H-NMR (400 MHz, DMSO-d_6_): δ = 2.29 (2s, 3H, CH_3_), 2.45–2.52 (m, 1H, CH_2_CO), 2.62 (t, J = 7.2 Hz, 1H, CH_2_CO), 3.25 (q, J = 6.6 Hz, 2H, NHCH_2_), 6.09 (s, 1H, CH_isox_), 6.43, 6.62 (2d, J = 8.7 Hz, 2H, H_ar_), 6.65–6.72 (m, 1H, NH), 6.90–7.72 (m, 7H, H_ar_), 10.95 (s, 1H, NH), 11.73 (s, 1H, NH) ppm.

IR (KBr): ν_max_ = 3347, 3319, 3157 (NH), 1699 (C = O), 1597 (C = N) cm^–1^.

Calcd for C_20_H_20_N_6_O_4_S, %: C 54.54; H 4.58; N 19.08. Found, %: C 54.39; H 4.62; N 18.92.

N-(5-methylisoxazol-3-yl)-4-((2-(4-phenyl-5-thioxo-4,5-dihydro-1H-1,2,4-triazol-3-yl)ethyl)amino)-benzenesulfonamide **(13)**

White solid, yield 0.88 g, 96%, m.p. 222–223°C.

^1^H-NMR (400 MHz, DMSO-d_6_): δ = 2.29 (s, 3H, CH_3_), 2.65 (t, J = 7.1 Hz, 2H, CH_2_CO), 3.29 (q, J = 6.8 Hz, 2H, NHCH_2_), 6.10 (s, 1H, CH_isox_), 6.43, 6.62 (2d, J = 8.6 Hz, 2H, H_ar_), 6.70 (t, J = 5.9 Hz, 1H, NH), 7.37–7.55 (m, 7H, H_ar_), 10.96 (s, 1H, NH), 13.77 (s, 1H, NH) ppm;

^13^C-NMR (101 MHz, DMSO-d_6_): δ = 12.07, 25.02, 39.00, 95.29, 110.85, 124.61, 128.41, 128.69, 129.42, 129.46, 133.61, 150.28, 151.80, 157.90, 167.65, 169.92 ppm.

IR (KBr): ν_max_ = 3354, 3259, 3159 (NH), 1598 (C = N) cm^–1^.

Calcd for C_20_H_20_N_6_O_3_S_2_, %: C 52.62; H 4.42; N 18.41. Found, %: C 52.22; H 4.39; N 18.62

N-(5-methylisoxazol-3-yl)-4-((3-oxo-3-(2-(2-oxoindolin-3-ylidene)hydrazineyl)propyl)amino)-benzenesulfonamide **(14)**

To a solution of hydrazide **4** (5 mmol, 1.7 g) in 1,4-dioxane (25 mL) isatin (5.25 mmol, 0.77 g) and acetic acid (3 drops) were added and the mixture was heated at reflux for 3 h, then cooled down, diluted with 40 mL of water and stirred for 1 h. Afterwards, the formed crystalline solid was filtered off, washed with water, diethyl ether and recrystallized from 1,4-dioxane.

Yellow solid, yield 1.26 g, 54%, m.p. 209–210°C.

^1^H-NMR (400 MHz, DMSO-d_6_): Z/E 75:25, δ = 2.28 (2s, 3H, CH_3_), 2.82–3.13 (m, 1H, CH_2_CO), 2.88–2.97 (m, 2H, CH_2_CO), 3.40–3.48 (m, 2H, NHCH_2_), 6.10 (s, 1H, CH_isox_), 6.63–6.79 (m, 3H, H_ar_, NH), 6.93 (d, J = 7.7 Hz, 1H, H_ar_), 7.07 (t, J = 7.6 Hz, 1H, H_ar_), 7.30–7.58 (m, 4H, H_ar_), 10.68, 10.79 (2s, 2H, 2NH), 12.57, 12.93 (2s, 1H, NH) ppm;

^13^C-NMR (101 MHz, DMSO-d_6_): 12.06, 30.72, 30.75, 37.66, 95.30, 110.97, 111.13, 119.75, 120.37, 122.57, 124.45, 127.04, 128.77, 131.47, 142.33, 152.25, 157.93, 162.49, 162.87, 169.91, 173.44 ppm.

IR (KBr): ν_max_ = 3567, 3395, 3247, 3158 (4NH), 1713, 1691 (2C = O), 1599 (2C = N) cm^–1^.

Calcd for C_21_H_20_N_6_O_5_S, %: C 53.84; H 4.30; N 17.94. Found, %: C 53.79; H 4.22; N 17.83.

General procedure for the preparation of hydrazones **15** and **16**

To a mixture of hydrazide **4** (5 mmol, 1.7 g), the corresponding carbaldehyde, benzaldehyde or 1-naphthaldehyde and 1,4-dioxane hydrochloric acid (**16, 15a-i**) or acetic acid (**15k**) (5 drops) was added dropwise and the mixture was heated at reflux for 4 (**16**), 24 (**15a-i**) or 2 (**15k**) h, then cooled down, the formed precipitate was filtered off, washed with 1,4-dioxane, diethyl ether and recrystallized from 1,4-dioxane.

4-((3-(2-Benzylidenehydrazineyl)-3-oxopropyl)amino)-N-(5-methylisoxazol-3-yl)benzenesulfonamide **(15a)**

White solid, yield 0.78 g, 36.4%, m.p. 199–200°C.

^1^H-NMR (400 MHz, DMSO-d_6_): Z/E 60:40, δ = 2.27, 2.28 (2s, 3H, CH_3_), 2.44–1.49 (overlaps with the signal of the DMSO-d_6_, 1H, CH_2_CO), 2.91 (t, J = 7.1 Hz, 1H, CH_2_CO), 3.35–3.42 (m, 2H, NHCH_2_), 6.09, 6.10 (2s, 1H, CH_isox_), 6.66 (t, J = 8.5 Hz, 2H, H_ar_), 6.71–6.85 (m, 1H, NH), 7.29–7.50 (m, 3H, H_ar_), 7.53 (d, J = 8.5 Hz, 2H, H_ar_), 7.56–7.79 (m, 2H, H_ar_), 8.01, 8.17 (2s, 1H, CH = N), 10.96 (br. s, 1H, NH), 11.39, 11.53 (2s, 1H, NH) ppm.

IR (KBr): ν_max_ = 3307, 3247 (NH), 1659 (C = O), 1601 (C = N) cm^–1^.

Calcd for C_20_H_21_N_5_O_4_S, %: C 56.19; H 4.95; N 16.38. Found, %: C 56.29; H 5.03; N 16.32.

4-((3-(2-(2-Hydroxybenzylidene)hydrazineyl)-3-oxopropyl)amino)-N-(5-methylisoxazol-3-yl)benzene-sulfonamide **(15b)**

Light grey solid, yield 1.53 g, 69%, m.p. 193–194°C.

^1^H-NMR (400 MHz, DMSO-d_6_): Z/E 60:40, δ = 2.29 (2s, 3H, CH_3_), 2.41–2.52 (overlaps with the signal of the DMSO-d_6_, 1H, CH_2_CO), 2.82–3.04 (m, 1H, CH_2_CO), 3.38–3.52 (m, 2H, NHCH_2_), 6.09, 6.10 (2s, 1H, CH_isox_), 6.55–7.05 (m, 5H, NH, H_ar_), 7.10–7.75 (m, 4H, H_ar_), 8.29, 8.32, 8.34 (3s, 1H, CH = N), 10.08 (s, 1H, OH), 10.96 (s, 1H, NH), 11.13, 11.15 (2s, 0.4H, NH), 11.34, 11.65 (2s, 0.6H, NH) ppm.

IR (KBr): ν_max_ = 3321, 3236 (NH), 1654 (C = O), 1600 (C = N) cm^–1^.

Calcd for C_20_H_21_N_5_O_5_S, %: C 54.17; H 4.77; N 15.79. Found, %: C 54.25; H 4.57; N 15.68.

4-((3-(2-(4-Hydroxybenzylidene)hydrazineyl)-3-oxopropyl)amino)-N-(5-methylisoxazol-3-yl)benzene-sulfonamide **(15c)**

Light yellow solid, yield 1.42 g, 64%, m.p. 218–219°C.

^1^H-NMR (400 MHz, DMSO-d_6_): Z/E 60:40, δ = 2.27, 2.28 (2s, 3H, CH_3_), 2.44, 2.87 (2t, J = 7.2 Hz, 2H, CH_2_CO), 3.36–3.46 (m, 2H, NHCH_2_), 6.10 (s, 1H, CH_isox_), 6.55–6.95 (m, 5H, H_ar_, NH), 7.38–7.72 (m, 4H, H_ar_), 7.89, 8.02 (2s, 1H, CH = N), 10.96 (s, 1H, NH), 11.17, 11.19 (2s, 1H, NH) ppm.

IR (KBr): ν_max_ = 3516, 3291 (NH), 1651 (C = O), 1603 (C = N) cm^–1^.

Calcd for C_20_H_21_N_5_O_5_S, %: C 54.17; H 4.77; N 15.79. Found, %: C 54.11; H 4.70; N 15.73.

4-((3-(2-(4-Fluorobenzylidene)hydrazineyl)-3-oxopropyl)amino)-N-(5-methylisoxazol-3-yl)benzene-sulfonamide **(15d)**

Light grey solid, yield 1.31 g, 59%, m.p. 213–214°C.

^1^H-NMR (400 MHz, DMSO-d_6_): Z/E 60:40, δ = 2.28, 2.29 (2s, 3H, CH_3_), 2.48 (overlaps with the signal of the DMSO-d_6_, 1H, CH_2_CO), 2.90 (t, J = Hz, 1H, CH_2_CO), 3.25–3.42 (m, 2H, NHCH_2_), 6.09, 6.10 (2s, 1H, CH_isox_), 6.59–6.78 (m, 3H, H_ar_, NH), 7.23–7.38 (m, 2H, H_ar_), 7.52 (d, J = 9.0 Hz, 2H, H_ar_), 7.62–7.97 (m, 2H, H_ar_), 7.98, 8.14 (2s, 1H, CH = N), 10.95 (s, 1H, NH), 11.38, 11.41 (2s, 1H, NH) ppm;

IR (KBr): ν_max_ = 3595, 3312, 3167 (NH), 1656 (C = O), 1601 (C = N) cm^–1^.

Calcd for C_20_H_20_FN_5_O_4_S, %: C 53.93; H 4.53; N 15.72. Found, %: C 53.88; H 4.57; N 15.76.

4-((3-(2-(2-Chlorobenzylidene)hydrazineyl)-3-oxopropyl)amino)-N-(5-methylisoxazol-3-yl)benzene-sulfonamide **(15e)**

White solid, yield 0.83 g, 36%, m.p. 202–203°C.

^1^H-NMR (400 MHz, DMSO-d_6_): Z/E 60:40, δ = 2.27, 2.28 (2s, 3H, CH_3_), 2.50 (overlaps with the signal of the DMSO-d_6_, 1H, CH_2_CO), 2.92 (t, J = 6.7 Hz, 1H, CH_2_CO), 3.37–3.43 (m, 2H, NHCH_2_), 6.09, 6.10 (2s, 1H, CH_isox_), 6.59–6.82 (m, 3H, H_ar_, NH), 7.31–7.63 (m, 5H, H_ar_), 7.82–7.97 (m, 1H, H_ar_), 8.38, 8.54 (2s, 1H, CH = N), 10.95 (s, 1H, NH), 11.57, 11.65 (2s, 1H, NH) ppm;

IR (KBr): ν_max_ = 3575, 3329, 3275 (NH), 1652 (C = O), 1602 (C = N) cm^–1^.

Calcd for C_20_H_20_ ClN_5_O_4_S, %: C 52.00; H 4.36; N 15.16. Found, %: C 51.91; H 4.42; N 15.08.

4-((3-(2-(4-Chlorobenzylidene)hydrazineyl)-3-oxopropyl)amino)-N-(5-methylisoxazol-3-yl)benzene-sulfonamide **(15f)**

White solid, yield 1.25 g, 54%, m.p. 210–211°C.

^1^H-NMR (400 MHz, DMSO-d_6_): Z/E 60:40, δ = 2.28, 2.29 (2s, 3H, CH_3_), 2.50 (overlaps with the signal of the DMSO-d_6_, 1H, CH_2_CO), 2.91 (t, J = 6.9 Hz, 1H, CH_2_CO), 3.36–3.44 (m, 2H, NHCH_2_), 6.09, 6.10 (2s, 1H, CH_isox_), 6.47–6.87 (m, 3H, H_ar_, NH), 7.46–7.53 (m, 3H, H_ar_), 7.58–7.91 (m, 3H, H_ar_), 7.98, 8.14 (2s, 1H, CH = N), 10.96 (s, 1H, NH), 11.44, 11.47 (2s, 1H, NH) ppm.

IR (KBr): ν_max_ = 3662, 3303, 3239 (NH), 1656 (C = O), 1600 (C = N) cm^–1^.

Calcd for C_20_H_20_ ClN_5_O_4_S, %: C 52.00; H 4.36; N 15.16. Found, %: C 52.08; H 4.29; N 15.22.

4-((3-(2-(4-Methoxybenzylidene)hydrazineyl)-3-oxopropyl)amino)-N-(5-methylisoxazol-3-yl)benzene-sulfonamide **(15g)**

Light grey solid, yield 1.44 g, 63%, m.p. 206–207°C.

^1^H-NMR (400 MHz, DMSO-d_6_): Z/E 60:40, δ = 2.27, 2.29 (2s, 3H, CH_3_), 2.45 (t, J = 7.0 Hz, 1H, CH_2_CO), 2.89 (t, J = 7.0 Hz, 1H, CH_2_CO), 3.35–3.41 (m, 2H, NHCH_2_), 3.79, 3.83 (2s, 3H, OCH_3_), 6.09, 6.10 (2s, 1H, CH_isox_), 6.65 (t, J = 8.7 Hz, 2H, H_ar_), 6.75 (t, J = 5.7 Hz, 1H, NH), 6.97–7.06 (m, 2H, H_ar_), 7.47–7.68 (m, 4H, H_ar_),7.81 (d, J = 8.5 Hz, 1H, H_ar_), 7.94, 8.08 (2s, 1H, CH = N), 10.95 (s, 1H, NH), 11.24, 11.28 (2s, 1H, NH) ppm;

IR (KBr): ν_max_ = 3269, 3239 (NH), 1654 (C = O), 1602 (C = N) cm^–1^.

Calcd for C_21_H_23_N_5_O_5_S, %: C 55.13; H 5.07; N 15.31. Found, %: C 55.79; H 5.17; N 15.53.

4-((3-(2-(3-Nitrobenzylidene)hydrazineyl)-3-oxopropyl)amino)-N-(5-methylisoxazol-3-yl)benzene-sulfonamide **(15h)**

White solid, yield 1.07 g, 45.5%, m.p. 208–209°C.

^1^H-NMR (400 MHz, DMSO-d_6_): Z/E 60:40, δ = 2.27, 2.28 (2s, 3H, CH_3_), 2.51–2.55 (overlaps with the signal of the DMSO-d_6_, 1H, CH_2_CO), 2.95 (t, J = 6.8 Hz, 1H, CH_2_CO), 3.40 (q, J = 6.5 Hz, 2H, NHCH_2_), 6.08, 6.10 (2s, 1H, CH_isox_), 6.58–6.84 (m, 3H, H_ar_, NH), 7.46–7.57 (m, 2H, H_ar_), 7.71 (q, J = 8.4 Hz, 1H, H_ar_), 7.99–8.34 (m, 3H, H_ar_), 8.43, 8.50 (2s, 1H, CH = N), 10.95 (s, 1H, NH), 11.63, 11.83 (2s, 1H, NH) ppm.

IR (KBr): ν_max_ = 3328, 3252 (NH), 1660 (C = O), 1601 (C = N) cm^–1^.

Calcd for C_20_H_20_N_6_O_6_S, %: C 50.84; H 4.27; N 17.79. Found, %: C 50.79; H 4.27; N 17.85.

4-((3-(2-(4-Nitrobenzylidene)hydrazineyl)-3-oxopropyl)amino)-N-(5-methylisoxazol-3-yl)benzene-sulfonamide **(15j)**

Yellow solid, yield 1.04 g, 44%, m.p. 214–215°C.

^1^H-NMR (400 MHz, DMSO-d_6_): Z/E 60:40, δ = 2.27, 2.28 (2s, 3H, CH_3_), 2.51–2.56 (overlaps with the signal of the DMSO-d_6_, 1H, CH_2_CO), 2.88–2.97 (m, 1H, CH_2_CO), 3.36–3.50 (m, 2H, NHCH_2_), 6.09, 6.10 (2s, 1H, CH_isox_), 6.53–6.87 (m, 3H, H_ar_, NH), 7.45–7.58 (m, 2H, H_ar_), 7.59–9.11 (m, 5H, H_ar_, CH = N), 10.96 (s, 1H, NH), 11.68, 11.72 (2s, 1H, NH) ppm.

IR (KBr): ν_max_ = 3431, 3323, 3272 (NH), 1682, 1659 (C = O), 1599 (C = N) cm^–1^.

Calcd for C_20_H_20_ N_6_O_6_S, %: C 50.84; H 4.27; N 17.79. Found, %: C 50.90; H 4.20; N 17.70.

4-((3-(2-(2-Chloro-5-nitrobenzylidene)hydrazineyl)-3-oxopropyl)amino)-N-(5-methylisoxazol-3-yl)benzenesulfonamide **(15i)**

Light yellow solid, yield 0.79 g, 31%, m.p. 210–211°C.

IR (KBr): ν_max_ = 3614, 3331, 3255 (NH), 1650 (C = O), 1599 (C = N) cm^–1^.

MS (ESI): m/z 505.16 [M-H]^+^.

Calcd for C_20_H_19_ClN_6_O_6_S, %: C 47.39; H 3.78; N 16.58. Found, %: C 47.48; H 3.71; N 16.53.

N-(5-methylisoxazol-3-yl)-4-((3-(2-(naphthalen-1-ylmethylene)hydrazineyl)-3-oxopropyl)amino)-benzenesulfonamide **(15k)**

White solid, yield 1.07 g, 45%, m.p. 206–207°C.

^1^H-NMR (400 MHz, DMSO-d_6_): Z/E 60:40, δ = 2.26, 2.29 (2s, 3H, CH_3_), 2.52–2.57 (overlaps with the signal of the DMSO-d_6_, 0.6H, CH_2_CO), 2.87–3.08 (m, 1.4H, CH_2_CO), 3.37–3.52 (m, 2H, NHCH_2_), 6.10, 6.12 (2s, 1H, CH_isox_), 6.60–6.84 (m, 3H, H_ar_, NH), 7.47–7.69 (m, 5H, H_ar_), 7.86 (d, J = 7.2 Hz, 1H, H_ar_), 8.00, 8.06 (2d, J = 8.1 Hz, 2H, H_ar_), 8.54, 8.85 (2d, J = 8.4 Hz, 1H, H_ar_), 8.71, 8.76 (2s, 1H, CH = N), 10.97 (s, 1H, NH), 11.45, 11.53 (2s, 1H, NH) ppm;

IR (KBr): ν_max_ = 3595, 3316, 3240 (NH), 1663 (C = O), 1599 (C = N) cm^–1^.

Calcd for C_24_H_23_N_5_O_4_S, %: C 60.36; H 4.85; N 14.67. Found, %: C 60.31; H 4.91; N 14.72.

4-((3-(2-(Furan-2-ylmethylene)hydrazineyl)-3-oxopropyl)amino)-N-(5-methylisoxazol-3-yl)benzene-sulfonamide **(16a)**

Light yellow solid, yield 1.17 g, 56%, m.p. 185–186°C.

^1^H-NMR (400 MHz, DMSO-d_6_): Z/E 60:40, δ = 2.28 (s, 3H, CH_3_), 2.43–2.47 (m, 0.7H, CH_2_CO), 2.83, 2.91 (2t, J = 7.1, 7.8 Hz, 1.3H, CH_2_CO), 3.35–3.48 (m, 2H, NHCH_2_), 6.10 (s, 1H, CH_isox_), 6.36–7.05 (m, 5H, H_ar_, NH), 7.53 (d, J = 8.5 Hz, 2H, H_ar_), 7.80 (d, J = 8.4 Hz, 1H, H_ar_), 7.88, 8.03 (2s, 1H, CH = N), 10.96 (s, 1H, NH), 11.33, 11.34 (2s, 1H, NH) ppm.

IR (KBr): ν_max_ = 3540, 3304, 3266 (NH), 1657 (C = O), 1600 (C = N) cm^–1^.

Calcd for C_18_H_19_N_5_O_5_S, %: C 51.79; H 4.59; N 16.78. Found, %: C 51.86; H 4.52; N 16.82.

N-(5-methylisoxazol-3-yl)-4-((3-(2-((5-nitrofuran-2-yl)methylene)hydrazineyl)-3-oxopropyl)amino)-benzenesulfonamide **(16b)**

Dark yellow solid, yield 1.29 g, 56%, m.p. 221–222°C.

^1^H-NMR (400 MHz, DMSO-d_6_): Z/E 60:40, δ = 2.28, 2.29 (2s, 3H, CH_3_), 2.51–2.55 (m, overlaps with the signal of the DMSO-d_6_, 2H, CH_2_CO), 3.37–3.42 (m, 2H, NHCH_2_), 6.09, 6.10 (2s, 1H, CH _isox_), 6.58–6.80 (m, 3H, H_ar_, NH), 7.17, 7.20 (2d, J = 4.0 Hz, 1H, H_ar_), 7.53 (d, J = 8.5 Hz, 2H, H_ar_), 7.77 (d, J = 3.9 Hz, 1H, H_ar_), 7.93, 8.11 (2s, 1H, CH = N), 10.94, 10.95 (2s, 1H, NH), 11.77, 11.80 (2s, 1H, NH) ppm;

IR (KBr): ν_max_ = 3410, 3345 (NH), 1679 (C = O), 1600 (C = N) cm^–1^.

Calcd for C_18_H_18_N_6_O_7_S, %: C 46.75; H 3.92; N 18.17. Found, %: C 46.86; H 4.01; N 18.22.

N-(5-methylisoxazol-3-yl)-4-((3-oxo-3-(2-(thiophen-2-ylmethylene)hydrazineyl)propyl)amino)-benzenesulfonamide **(16c)**

Light yellow solid, yield 1.52 g, 70%, m.p. 215–216°C.

^1^H-NMR (400 MHz, DMSO-d_6_): δ = 2.28, 2.29 (2s, 3H, CH_3_), 2.45 (t, J = 7.0 Hz, 1H, CH_2_CO), 2.83 (t, J = 7.0 Hz, 1H, CH_2_CO), 3.35–3.41 (m, 2H, NHCH_2_), 6.10 (s, 1H, CH _isox_), 6.65 (d, J = 8.6 Hz, 2H, H_ar_), 6.70–6.80 (m, 1H, NH), 7.00–7.19 (m, 1H, H_ar_), 7.39, 7.42 (2d, J = 3.6 Hz, 1H, H_ar_), 7.53 (d, J = 8.5 Hz, 2H, H_ar_), 7.61, 7.63 (2d, J = 5.0 Hz, 1H, H_ar_), 8.17, 8.36 (2s, 1H, CH = N), 10.95 (s, 1H, NH), 11.34 (s, 1H, NH) ppm.

IR (KBr): ν_max_ = 3638, 3295 (NH), 1652 (C = O), 1601 (C = N) cm^–1^.

Calcd for C_18_H_19_N_5_O_4_S_2_, %: C 49.87; H 4.42; N 16.16. Found, %: C 50.06; H 4.49; N 16.28.

N-(5-methylisoxazol-3-yl)-4-((3-(2-((5-nitrothiophen-2-yl)methylene)hydrazineyl)-3-oxopropyl)amino)-benzenesulfonamide **(16d)**

Yellow solid, yield 1.05 g, 44%, m.p. 195–196°C.

^1^H-NMR (400 MHz, DMSO-d_6_): Z/E 60:40, δ = 2.27, 2.29 (2s, 3H, CH_3_), 2.51–2.53 (m, overlaps with the signal of the DMSO-d_6_, 1H, CH_2_CO), 2.88 (t, J = 6.9 Hz, 1H, CH_2_CO), 3.37 (q, J = 6.9 Hz, 2H, NHCH_2_), 6.09 (br. s, 1H, CH_isox_), 6.65 (d, J = 8.6 Hz, 2H, H_ar_), 6.70–6.79 (m, 1H, NH), 7.49 (d, J = 4.4 Hz, 1H, H_ar_), 7.53 (d, J = 8.6 Hz, 2H, H_ar_), 8.10 (t, J = 3.9 Hz, 1H, H_ar_), 8.17, 8.42 (2s, 1H, CH = N), 10.95 (br. s, 1H, NH), 11.80, 11.88 (2s, 1H, NH) ppm.

IR (KBr): ν_max_ = 3647, 3334, 3246 (NH), 1656 (C = O), 1601 (C = N) cm^–1^.

Calcd for C_18_H_18_N_6_O_6_S_2_, %: C 45.18; H 3.79; N 17.56. Found, %: C 45.01; H 3.73; N 17.88.

### Pharmacology

#### Cell culturing

The human clear cell renal cell carcinoma line (CaKi-1) was obtained from American Type Culture Collection (ATCC, Manassas, VA). The human prostate carcinoma cell line PPC-1 was originally obtained from ATCC and kindly provided by Prof. Tambet Teesalu (University of Tartu, Estonia). Human foreskin fibroblasts (HF) CRL-4001 were originally obtained from ATCC and kindly provided by Prof. Helder Santos (University of Helsinki, Finland). Cells were cultured in Dulbecco’s Modified Eagle’s GlutaMAX medium (Gibco (Carlsbad, CA, USA) supplemented with 10,000 U/mL penicillin, 10 mg/mL streptomycin (Gibco), and 10% fetal bovine serum (Gibco). During cell passaging, Tryple Express reagent (Gibco) was used for cell detachment. Cell cultures were grown at 37°C in a humidified atmosphere containing 5% CO_2_. They were used until the passage of 20.

#### Compound effect on cancer cell viability

The effect of synthesized compounds on cell viability was studied using 3-(4,5-dimethylthiazol-2-yl)-2,5-diphenyltetrazolium bromide (MTT; Sigma-Aldrich Co., St Louis, MO, USA) assay, as described elsewhere [46]. Briefly, HF, PPC-1, and CaKi-1 cells were seeded in 96-well plates (Corning) in triplicates at a volume of 100 μL at a concentration of 4 × 10^3^ cells/well. After 24 h of incubation, the cells were treated with 100 μM of tested compounds. After 72 h, the MTT reagent (at 0.5 mg/mL concentration) has been added to the wells, and cells were incubated for 4 h. Then the medium was aspirated, and the formed purple crystals were dissolved in 100 μL DMSO (Sigma-Aldrich Co.). The absorbance was measured at 570 and 630 nm using a multi-detection microplate reader. The compound effect on cell viability was calculated using a formula (1):

$$Relative\;cell\;viability\;\left(\%\right)=\;\frac{A-\;A_{0}}{A_{NC}-A_{0}}$$ (1)

Where:

A–mean of absorbance of tested compound,

A_0_–mean of absorbance of blank (no cells, positive control),

A_NC_–mean of absorbance of negative control (only cells, no treatment).

The EC_50_ values of the most active compounds **15e**, **15k**, **16b**, and **16d** was established by the same MTT procedure. The compound serial dilutions from 100 μM to 3.125 μM have been made in a medium and added to the cells in triplicates. EC_50_ value that represent the concentration of a compound causing 50% reduction of cancer cell metabolic activity has been calculated using Hill equation.

#### Antimicrobial activity by serial dilution in a liquid media

Antibacterial and antifungal susceptibility to the compound **16b** was tested using a serial broth dilution technique in Mueller-Hinton broth II (BBL, Cockeysville, USA), as described elsewhere [47]. All procedures were done in aseptic conditions. Standard bacterial and fungal cultures were used: *Staphylococcus aureus* ATCC 25923, *Staphylococcus epidermidis* ATCC 12228, *Escherichia coli* ATCC 25922, *Enterococcus faecalis* ATCC 29212, *Klebsiella pneumoniae* ATCC 33499, *Pseudomonas aeruginosa* ATCC 27853, *Proteus mirabilis* ATCC 12459, *Bacillus cereus* ATCC 8035, and *Candida albicans* ATCC 60193.

Standard cultures of nonsporic bacteria *S*. *aureus*, *S*. *epidermidis*, *E*. *coli*, *E*. *faecalis*, *K*. *pneumoniae*, *P*. *aeruginosa* and *P*. *mirabilis* were cultivated for 20–24 hours at 37°C in Mueller-Hinton Agar (BBL, Cockeysville, USA). A bacterial suspension was prepared from cultivated bacterial cultures in physiological solution according to turbidity standard 0.5 McFarland. Standard culture of sporic bacteria *B*. *cereus* was cultivated for 7 days at 37°C in Mueller-Hinton II Agar. After sporic bacteria culture had grown, it was washed away from the surface of the broth with sterile physiological solution, and the prepared suspension was being heated for 30 min. at the temperature of 70°C and diluted till the concentration of spores in 1 ml ranged from 10 x 10^6^ to 100 x 10^6^. The fungal culture C. albicans was cultivated for 20–24 hours at 30 ºC in Mueller-Hinton agar. A fungal suspension was prepared in physiological solution according to the turbidity standard 0.5 McFarland.

The compound 16B was dissolved in dimethylsulfoxide right before experiment at a concentration of 20 mM. Then compound serial dilutions from 200 μM to 6.25 μM have been made in a Mueller-Hinton broth and added to the 96-well plates in triplicates at a volume of 50 μl. The prepared microbial cultures were diluted in a Mueller-Hinton brith 150 times and added to the 96-well plate at a volume of 50 μL. In such a way, the final compound concentrations were in the range from 100 μM to 3.125 μM. The wells with only medium served as a positive control, and the wells containing microbial suspension and 0.5% dimethylsulfoxide served as a negative control. The 96-well plates were incubated at 37 ºC for 24 hours (plates with the fungal culture were incubated for 48 hours). Then the absorbance was measured at 600 nm using a multi-detection microplate reader. EC_50_ value that represent the concentration of a compound causing 50% reduction of microbial growth has been calculated using Hill equation.

#### Antimicrobial activity by serial dilution on a solid agar

Antimicrobial activity of compound **16b** on solid agar was tested using the same microbial cultures, and the same suspension preparation procedure, as described in above section. All procedures were done in aseptic conditions.

Mueller-Hinton agar was boiled and kept at to 37 ºC during the whole procedure. Compound **16b** solution was prepared in DMSO right before experiment. The Agar was mixed with compound solution at different ratios to make the final compound concentrations of 1 mM, 400 μM, 200 μM and 100 μM. Then 10 ml of prepared solution was poured into sterile Petri dishes (85 mm in diameter) and kept in aseptic conditions while the agar solidified. The agar with 0.5% of DMSO served as a negative control. With the help of loop standard microbial cultures by 0.1 ml were poured on the surface of prepared agar with investigative compound. Then Petri dishes were kept for 24 hours in thermostatic conditions.

The minimal dilution, i.e. the lowest concentration in μg/mL of the investigative and comparison compounds that inhibited visible growth of microorganisms was the minimal inhibitory concentration (MIC).

#### Statistical analysis

All biological experiments were repeated at least three times, calculating the mean and standard deviation. The data was processes using Microsoft Office Excel 365 software (Microsoft Corporation, Redmond, WA, USA). Statistical analysis was performed by using Student’s t-test. The level of significance was set as p < 0.05.

## Results and discussion

### Chemistry

In this study, sulfamethoxazole was used as initial compound. To obtain carboxylic acid **2** (Scheme 1) sulfamethoxazole **1** was reacted with acrylic acid in water. The refluxing of the reaction mixture for 24 hours yielded compound **2**, which then was applied for the preparation of derivatives **3–7**.

**Scheme 1. Synthesis of sulfamethoxazole derivatives 2–7. 5** X = O, **6**, **6a** X = S. Reagents and conditions: (a) acrylic acid, water, Δ, 24 h; (b) MeOH, H_2_SO_4_, Δ, 26 h, aqueous 5% Na_2_CO_3_; (c) N_2_H_4_∙H_2_O, 2-PrOH, Δ, 24 h; (d) (NH_2_)_2_CO or KSCN, AcOH, Δ, 26 h; (e) 15% HCl, Δ, 1 h, water; (f) 5% NaOH, r.t., 24 h, AcOH to pH 6–7; (g) sulphanilamide, DMSO, NEt_3_, HBTU, r.t., 20 h, water.

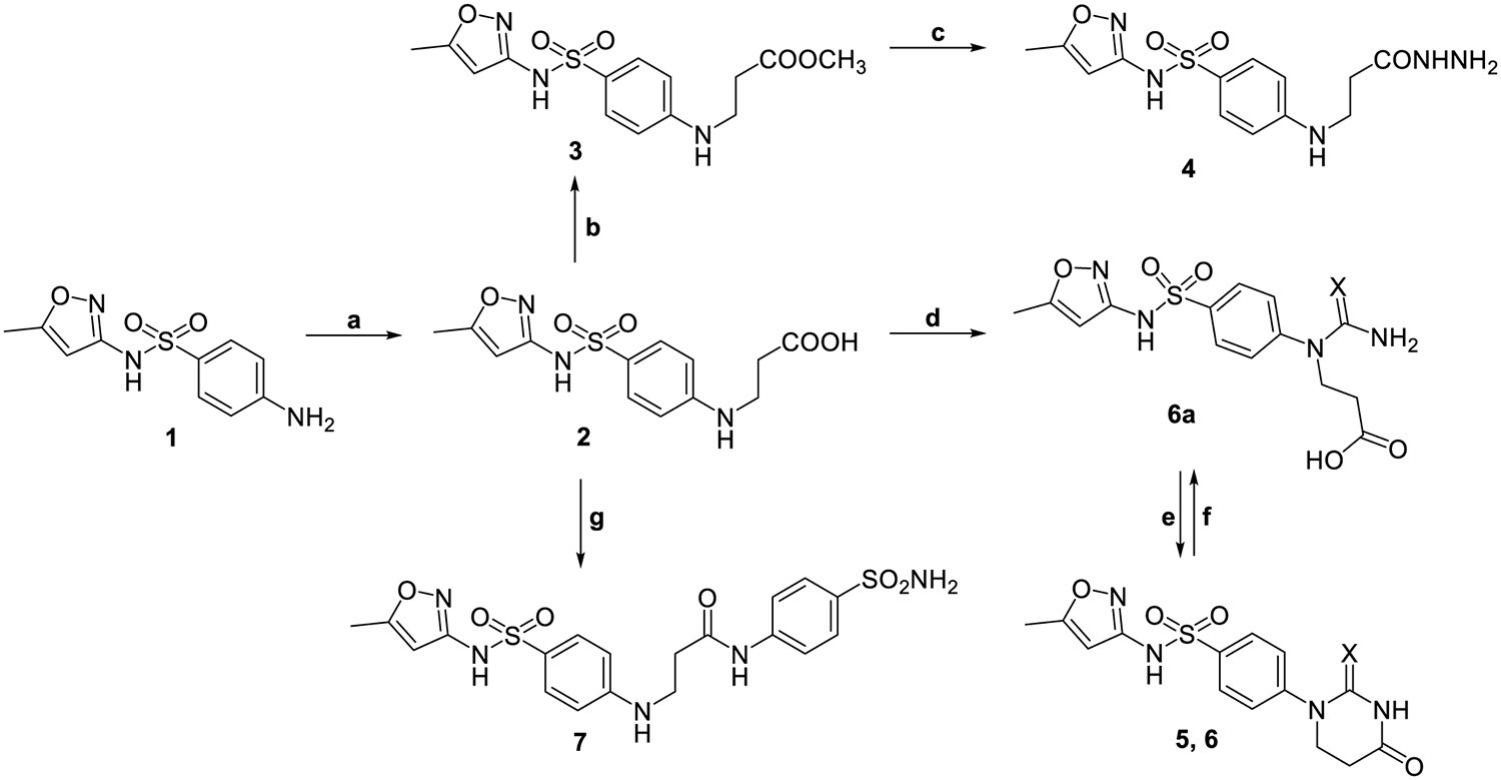


In the route to acid hydrazide **4** ester **3** was prepared and used as a building block in the reaction with hydrazine monohydrate. A viscous product **4** was separated from the reaction mixture after heating at reflux in propan-2-ol for 24 hours.

The interaction of β-alanine **2** with urea or potassium thiocyanate in refluxing acetic acid followed by the acidification of the reaction mixture with hydrochloric acid to cyclize the intermediate ureido or thioureido acid resulted in 1-aryl-5,6-dihydrouracil **5** and its 2-thio analogue **6**, respectively. In the ^1^H NMR spectra, the additional peaks at 11.45 ppm (**5**) and 11.55 ppm (**6**) were assigned to the protons of NH groups of the cyclic fragment. The ^13^C NMR spectrum of compound **5** showed resonances at 157.48 and 170.42 ppm, which were attributed to 2C = O of the formed cycle, as well as in the spectrum of compound **6** spectral lines at 170.48 and 179.62 (**6**) ppm confirmed a ring bearing the carbonyl and thiocarbonyl groups. To isolate intermediate **6a** a solution of thioxotetrahydropyrimidinone **6** in 5% sodium hydroxide was stirred at room temperature for 24 hours then filtered off and the filtrate was acidified with glacial acetic acid to pH 6–7. The comparison of the ^1^H NMR spectra of **6** and **6a** demonstrated the characteristic shifts of the protons’ triplets of the NCH_2_CH_2_ fragment from 2.81 and 3.94 ppm for **6** to 2.54 and 4.14 ppm for **6a**. Also, the OH hydrogen appearing at 12.24 ppm and broad singlet of the NH_2_ protons in the aromatic area at 7.22 ppm in the ^1^H NMR spectrum of thioureido acid **6a** confirmed the obtained structure.

The formation of amide **7** was performed by coupling carboxylic acid **2** and sulfanilamide with HBTU as coupling agent and TEA in dimethyl sulfoxide for 20 hours at room temperature. The dilution of the reaction mixture with water afforded the target compound **7**. The ^1^H and ^13^C NMR spectra of compound **7** are in excellent agreement with the predicted ones.

Another goal of this study was the synthesis of azole derivatives. Acid hydrazide **4** as a versatile precursor for azole synthesis was used for this purpose. To synthesized 2,5-dimethylpyrrole **8** and 3,5-dimethylpyrazole **9** hydrazide **4** was treated with hexane-2,5-dione or pentane-2,4-dione, respectively, in 1,4-dioxane for 1 hour. The reactions were catalysed by acetic (**8**) or hydrochloric (**9**) acid.

In the NMR spectra of compound **8** the singlets at 5.61 and 5.66 ppm (^1^H) and resonance at 102.88 ppm (^13^C) are characteristic of the protons and carbons of the 2CH of the pyrrole cycle and approve the presence of this structure (Scheme 2). Meanwhile, singlets at 2.16 and 2.46 ppm (2CH_3_) and an intense singlet at 6.17 ppm (CH) in the ^1^H NMR spectrum of compound **9** as well as spectral lines at 13.45, 14.08 (2CH_3_) and at 110.93 (CH_pyraz_) ppm in the ^13^C NMR spectrum confirm the formed 3,5-dimethylpyrazole ring.

**Scheme 2. Synthesis of compounds 8–16. 10, 12** X = O, **11, 13** X = S; **15a** Ar: C_6_H_5_; **15b** Ar: 2-HO-C_6_H_4_; **15c** Ar: 4-HO-C_6_H_4_; **15d** Ar: 4-F-C_6_H_4_; **15e** Ar: 2-Cl-C_6_H_4_; **15f** Ar: 4-Cl-C_6_H_4_; **15g** Ar: 4-H_3_CO-C_6_H_4_; **15h** Ar: 3-O_2_N-C_6_H_4_; **15j** Ar: 4-O_2_N-C_6_H_4_; **15i** Ar: 2-Cl-5-O_2_N-C_6_H_3_; **15k** Ar: 1-naphthyl; **16a** R: 2-furyl; **16b** R: 5-nitro-2-furyl; **16c** R: 2-thienyl; **16d** R: 5-nitro-2-thienyl. Reagents and conditions: (a) hexane-2,5-dione, 1,4-dioxane, AcOH, Δ, 1 h; (b) pentane-2,4-dione, 1,4-dioxane, HCl, Δ, 1 h; (c) PhNCO or PhNCS, MeOH, Δ, 24 h; (d) 4% NaOH, Δ, 4 h, HCl: H_2_O 1:1 to pH 2; (e) isatin, MeOH, AcOH, Δ, 3 h, water; (f) corresponding aldehyde, 1,4-dioxane, HCl, (AcOH for **15k**), Δ, 24 h for **15a–i**, 2 h for **15k** and 4 h for **16**.

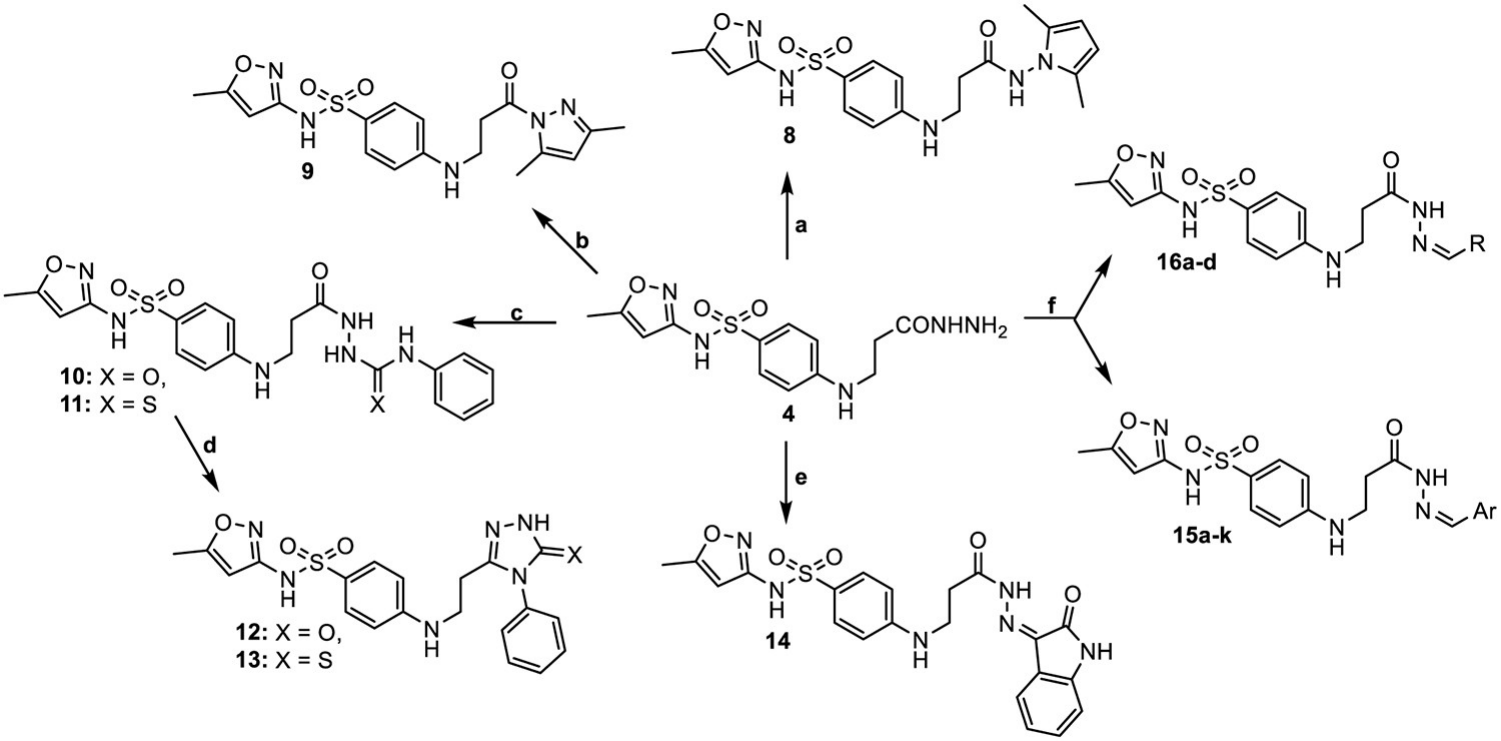


Semicarbazide **10** and thiosemicarbazide **11** were prepared upon refluxing compound **4** with phenyl isocyanate or isothiocyanate in methanol. Afterwards, they were cyclized to triazoles **12** and **13** by refluxing in aqueous 4% sodium hydroxide solution for 4 hours and subsequent acidification of the reaction mixture with diluted hydrochloric acid to pH 2. Comparison of the NMR spectra of compounds **10**, **11** and **12**, **13** demonstrated obvious characteristic differences, which approve the target structures.

In the ^1^H NMR spectrum of semicarbazide **10**, singlets at 8.03, 8.36, 8.70, 8.90, 9.03 and a sharp single peak at 9.74 ppm were observed due to the presence of a CONHNHCONH moiety protons. The same spectrum of its thio analogue **11** displayed peaks at 9.04, 9.35, 9.55, 9.85, 9.95 and 9.99 ppm integrated for 3 protons which confirm the presence of a CONHNHCSNH fragment. Analysing the ^1^H NMR spectra of their cyclic derivatives **12** and **13**, intense peaks in the down-field of the spectra (11.73 ppm for **12** and 13.77 ppm for **13**) were observed. They were attributed to the NH of the triazole ring. The ^13^C NMR spectra of compounds **10**–**13** showed resonances in agreement with target structures.

When acid hydrazide **4** was treated with isatin in the equimolar amount in 1,4-dioxane in the presence of a catalyst glacial acetic acid at 100°C for 3 hours the hydrazone **14** possessing the 2-oxindole moiety was obtained. The success of the reaction was confirmed by the data of spectroscopy and the elemental analysis. In the ^1^H NMR spectrum, the double sets of the peaks of the NHCO proton arise at 10.68, 10.79 and 12.57, 12.93 ppm with the intense ratio of 25:75, indicating the presence of a mixture of the *Z/E* isomers in DMSO-*d*_*6*_ solution, where usually the *Z*-form predominates [48, 49]. In the ^1^H NMR spectrum of **14** the resonance of the *Z* isomer appeared at a lower-field area of the spectrum with the respect of the peak of the *E*-form [50]. The ^13^C NMR spectrum exhibited the characteristic resonances belonging to the carbons of the 2-oxindole and amide carbonyls, which appeared at 162.49 and 169.91 ppm.

Hydrazones **15a–k**, as well as **16a–d**, were prepared by condensing of hydrazide **4** with the corresponding aromatic aldehyde (for **15**) or carbaldehyde (for **16**) in refluxing 1,4-dioxane with the addition of hydrochloric acid as a catalyst. To obtain hydrazone **15k** with the 1-naphthalenyl moiety in the molecule catalytic amount of glacial acetic acid was used instead of HCl. The ^1^H NMR spectra of the target hydrazones showed a mixture of two isomers in DMSO-*d*_*6*_ solutions which form due to the presence of an amide bond in the structures and the restricted rotation around this bond. The resulting hydrazones exist as *Z* and *E*-amide conformers with an intensity ratio found to be 60:40 with the Z-conformer predominating [48, 51] and arising up-field for almost all hydrazones except **16c** based on NH signal integration. In the ^1^H NMR spectrum of compound **16c** with the thiophene substituent in the molecule, only one intense peak for NH proton at 11.34 ppm was found. Comparison of the ^1^H NMR spectra of hydrazide **4** with hydrazones **15** and **16** showed a substantial difference between the chemical shifts of their NH signals. In the spectra of **15** and **16** double sets of the amide NH proton signals were visible in the range of 11.13–11.88 ppm while the hydrazide NH proton signal appeared at 9.03 ppm. In addition, the aromatic region of the spectra revealed an increase in the peaks integrated for the corresponding number of protons of the newly attached aromatic ring.

After confirming the structures of the resulting compounds, they were used for the evaluation of their anticancer and antimicrobial properties.

### Pharmacology

#### Anticancer activity

**Fig 2:**
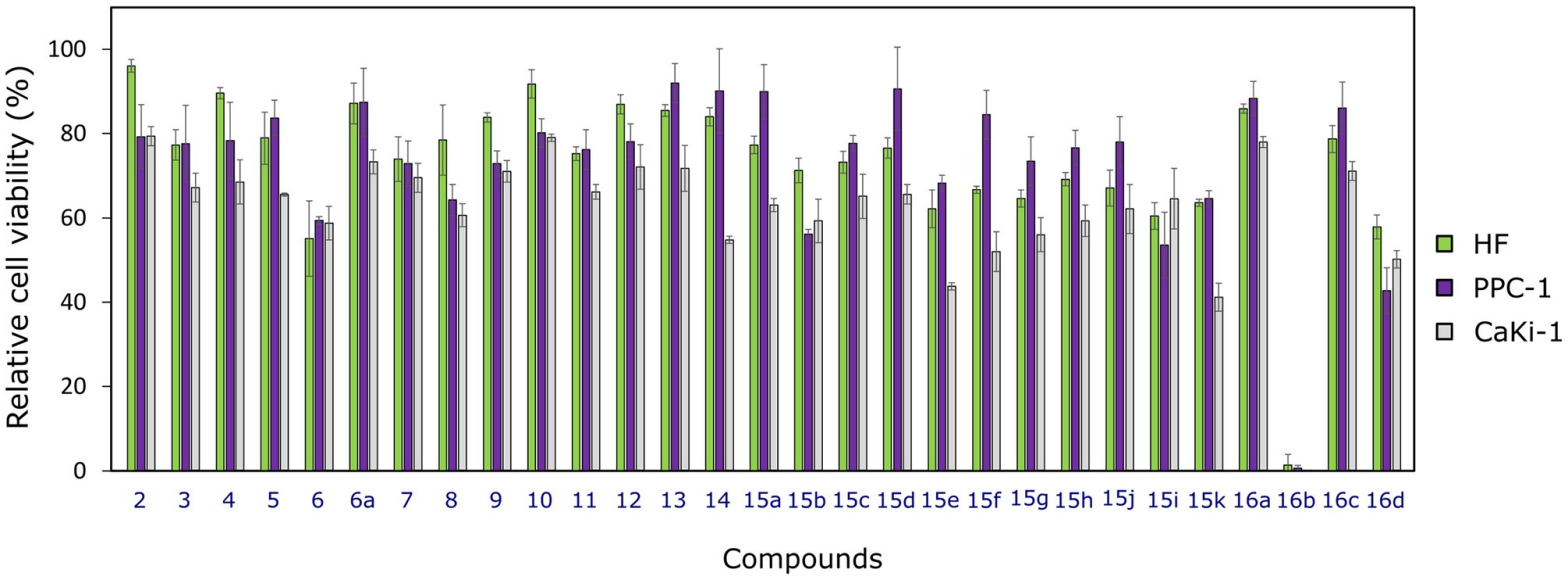
Effect of compounds on cancer cell viability at 100 μM concentration against human prostate carcinoma PPC-1, human kidney carcinoma CaKi-1 cell lines, and human fibroblasts HF, n = 3.

Tested compounds demonstrated different activity against human prostate carcinoma PPC-1 and kidney carcinoma CaKi-1 cell lines (Fig 2). The cytotoxicity of sulfamethoxazole derivatives against both types of cancer cells was similar. CaKi-1 cells were slightly more sensitive to several compounds, especially **14, 15a, 15d, 15f, 15g, 15h, 15j**, and **15k** containing phenyl, 4-fluorophenyl-, 4-chlorophenyl-, 4-methoxyphenyl-, 3- and 4-nitrophenyl as well as 1-naphthyl substituents in the structure. In general, compound selectivity towards cancer cell lines was not high. The most active compound was **16b**, and it showed practically the same effect on the viability of all tested cell cultures (Fig 2). The result was expected due to the presence of a 5-nitrofuryl moiety in the hydrazone molecule, as such type of compounds are characterized by the pronounced anticancer activity [52].

For further experiments four most promising compounds were selected, that reduced cell viability up to about 40% and more, namely, compounds **15e, 15k, 16b**, and **16d**.

**Fig 3:**
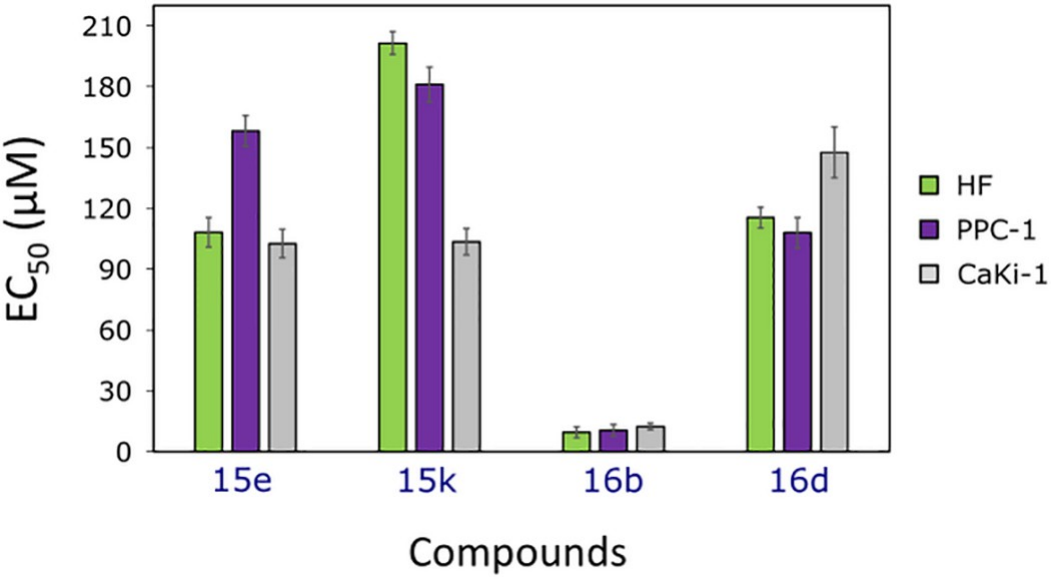
EC_50_ values of the most active sulfamethoxazole derivatives 15e, 15k, 16b, and 16d, obtained by MTT assay, after 72 hours of incubation, n = 3.

Compound **16b** was confirmed to be the most active one of all tested sulfamethoxazole derivatives (Fig 3), however, it was not selective towards cancer cells (EC_50_ = 10.4 ± 3.0 μM against PPC-1, EC_50_ = 12.4 ± 1.6 μM against CaKi-1, and EC_50_ = 9.5 ± 2.7 μM against HF). The least active and selective was compound **15k** (EC_50_ = 181.0 ± 8.5 μM against PPC-1, EC_50_ = 103.5 ± 6.5 μM against CaKi-1, and EC_50_ = 201.5 ± 5.6 μM against HF). The compound **15e** showed a similar effect on CaKi-1 and HF cell viability, while activity against PPC-1 was even lower. However, the activity of compounds in cell monolayer may not reflect the activity *in vivo* [53], thus their effects on three-dimensional models would be worthy to study, taking into account that such systems better represent tumour microenvironment and its properties [54].

Metastatic renal cell carcinoma is a very aggressive cancer type, and the 5-year survival rate of the patients is less than 12% [55]. Tyrosine kinase inhibitors, such as sorafenib, are approved to treat this cancer, but in most of patients these drugs become not effective due to the development of cancer resistance to the treatment [56]. The activity of sunitinib against CaKi-1 cells is higher (EC_50_ = 2.2 μM) [57] compared to the most active tested sulfamethoxazole derivative in our studies. On the other hand, two anticancer compounds that are also approved for the treatment of renal cancer, axitinib and pazopanib, possess lower activity (EC_50_ is 25 and 30 μM after 72 hours of incubation, accordingly) [58]. Thus, the activity of most active identified compounds from tested sulfamethoxazole derivatives is comparable, and they could be modified and further developed for this type of cancer.

Despite relatively good response to the available treatment, prostate cancer is the fifth leading cause of death worldwide [59]. It affects usually older population of men, and the treatment could be problematic. There is a need for newer, more effective agents [60]. The most active compound **16b** shows even higher activity in PPC-1 cell line than the approved anticancer drug, PARP inhibitor, Olaparib (EC_50_ values in prostate cancer cell lines are in range from 5.4 to even 73.2 μM) [61].

In summary, synthesized sulfamethoxazole derivatives show relatively moderate cytotoxic effect on prostate and kidney carcinoma cell lines and are not very selective. The most active candidate bearing a 5-nitrofuryl fragment shows a promising activity against both cancer cell lines. However, it could be mentioned, that 2-chlorophenyl, naphthalen-1-yl and 5-nitrothiophen-2-yl moieties are also worthy of attention and could be further used for searching and developing more selective compounds against both types of cancer.

#### Antimicrobial activity

Compound **16b** showed relatively high activity in cancer cell lines, but it was not very selective to them compared to fibroblasts. Sulfamethoxazole is widely used as antimicrobial medicinal substance, thus we decided to explore the effects of this compound against different microbial cultures.

**Fig 4:**
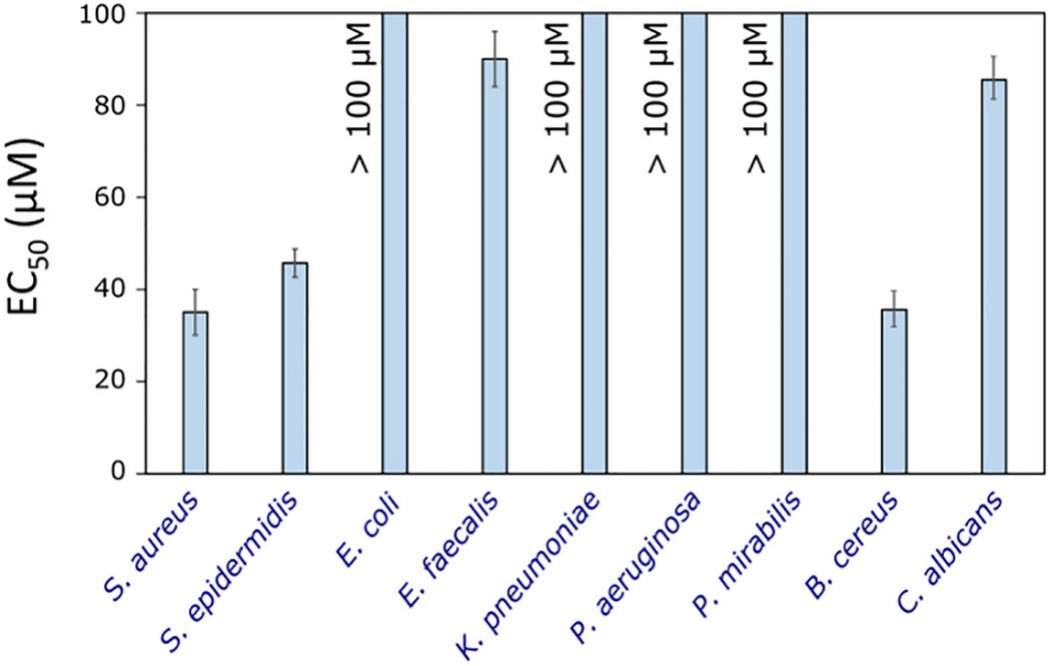
Antimicrobial activity of compound 16b in a liquid medium, expressed as half maximal effect concentration (EC_50_ values), n = 3.

Compound **16b** was not active against gram-negative bacteria *E*. *coli*, *K*. *pneumoniae*, *P*. *aeruginosa*, and *P*. *mirabilis* (Fig 4). The most sensitive were gram-positive cocci *S*. *aureus* and *S*. *epidermidis*, also a spore-forming rod *B*. *cereus*. Gram-positive anaerobic bacteria *E*. *faecalis* and fungus *C*. *albicans* were sensitive only at very high concentrations (EC_50_ = 90.0 ± 6.0 μM and EC_50_ = 85.5 ± 7.1 μM, respectively).

Similar results were obtained from experiments on solid agar (Table 1). The MICs values were higher compared to the EC_50_ values, and this observation is consistent with those made but other researchers [62].

**Table 1:** MIC values obtained by serial dilution of compound 16b on a solid agar.

Microbe	MIC (μM)
***S*. *aureus***	100
***S*. *epidermidis***	100
***E*. *coli***	400
***E*. *faecalis***	1000
***K*. *pneumoniae***	1000
***P*. *aeruginosa***	>1000
***P*. *mirabilis***	1000
***B*. *cereus***	400
***C*. *albicans***	400

Activity of compound **16b** was higher against *S*. *aureus* compared to its original structural component–sulfamethoxazole, which is still used nowadays in combination with trimethoprim [63]. Considering the fact that most gram-negative microorganisms are not sensitive to sulfamethoxazole, it could be possible to explain the selectivity of **16b** towards gram-positive bacteria [64]. Incorporation of 5-nitro-2-furyl fragment in its structure did not grant the activity against gram-negative rods, despite this structural component is present in nitrofurans and is responsible for their mechanism of action [65]. In summary, **16b** compound could be a potential drug-lead molecule for the new antimicrobial agents, especially those acting on drug resistant bacteria.

## Conclusions

A series of 3-((4-(N-(5-methylisoxazol-3-yl)sulfamoyl)phenyl)amino)propanoic acid and its hydrazide derivatives were synthesized and evaluated for their anticancer and antimicrobial activity. Hydrazone with a 5-nitrofuryl moiety in the structure was found to show the best efficiency on the viability of tested cancer cell cultures, i.e. human prostate carcinoma PPC-1 and human kidney carcinoma CaKi-1 cell lines. The results of an antimicrobial test in a liquid medium demonstrated that gram-positive bacteria strains of *S*. *aureus* and *S*. *epidermidis* and *B*. *cereus* were the most sensitive to its action, while gram-negative bacteria strains of *E*. *coli*, *K*. *pneumoniae*, *P*. *aeruginosa*, and *P*. *mirabilis* showed complete resistance for compound.

## Supporting information

S1 File Additional ^1^H, ^13^C NMR and MS information. (DOCX)

## References

[1] Jampilek J. Heterocycles in Medicinal Chemistry. *Molecules*. 2019; 24: 3839. doi: 10.3390/molecules24213839 31731387 PMC6864827

[2] M PH, Al-Ostoot FH, Vivek HK, Khanum ShA. Synthesis, characterization, DFT, docking studies and molecular dynamics of some 3-phenyl-5-furan isoxazole derivatives as anti-inflammatory and anti-ulcer agents. *J*. *Mol*. *Struct*. 2022; 1250: 131812. doi: 10.1016/j.molstruc.2021.131812

[3] Araya GCh, Kaur K, Jaitak V. Isoxazole derivatives as anticancer agent: A review on synthetic strategies, mechanism of action and SAR studies. Eur. J. Med. Chem. 2021; 221: 113511. doi: 10.1016/j.ejmech.2021.113511 34000484

[4] Aktaş DA, Akinalp G, Sanli F, Yucel MA, Gambacorta N, Nicolotti O, et al. Design, synthesis and biological evaluation of 3,5-diaryl isoxazole derivatives as potential anticancer agents. Bioorg. Med. Chem. Lett. 2020; 30: 127427. doi: 10.1016/j.bmcl.2020.127427 32750679

[5] Wang G, Liu W, Huang Y, Li Y, Peng Zh. Design, synthesis and biological evaluation of isoxazole-naphthalene derivatives as anti-tubulin agents. Arab. J. Chem. 2020; 13: 5765–5775. doi: 10.1016/j.arabjc.2020.04.014

[6] Bommagani MB, Yerrabelly JR, Chitneni M, Thalari G, Vadiyala NR, Boda SK, et al. Synthesis and antibacterial activity of novel cinnoline-isoxazole derivatives. Chem. Data Collect. 2021; 31: 100629. doi: 10.1016/j.cdc.2020.100629

[7] Kang YK, Shin KJ, Yoo KH, Seo KJ, Hong ChY, Lee Ch-S, et al. Synthesis and antibacterial activity of new carbapenems containing isoxazole moiety. *Bioorg*. *Med*. *Chem*. *Lett*. 2000; 10: 95–99. doi: 10.1016/s0960-894x(99)00646-0 10673088

[8] Alshamari A, Al-Qudah M, Hamadeh F, Al-Momani L, Abu-Orabi S. Synthesis, antimicrobial and antioxidant activities of 2-isoxazoline derivatives. *Molecules*. 2020; 25: 4271. doi: 10.3390/molecules25184271 32961855 PMC7570493

[9] Padmaja A, Rajasekhar C, Muralikrishna A, Padmavathi V. Synthesis and antioxidant activity of oxazolyl/thiazolylsulfonylmethyl pyrazoles and isoxazoles. *Eur*. *J*. *Med*. *Chem*. 2011; 46: 5034–5038. doi: 10.1016/j.ejmech.2011.08.010 21864949

[10] Zimecki M, Bąchor U, Mączyński M. Isoxazole derivatives as regulators of immune functions. *Molecules*. 2018; 23: 2724. doi: 10.3390/molecules23102724 30360408 PMC6222914

[11] Gutiérrez M, Amigo J, Fuentes E, Palomo I, Astudillo L. Synthetic isoxazole as antiplatelet agent. *Platelets*. 2014; 25: 234–238. doi: 10.3109/09537104.2013.807335 23841686

[12] Vicentini CV, Romagnoli C, Manfredini S, Rossi D, Mares D. Pyrazolo [3, 4-c] isothiazole and isothiazolo [4, 3-d] isoxazole derivatives as antifungal agents. *Pharm*. *Biol*. 2011; 49: 545–552. doi: 10.3109/13880209.2010.527350 21385093

[13] Frølund B, Jensen LS, Storustovu SI, Stensbøl TB, Ebert B, Kehler J, et al. 4-Aryl-5-(4-piperidyl)-3-isoxazolol GABA A antagonists: synthesis, pharmacology, and structure–activity relationships. J. Med. Chem. 2007; 50: 1988–1992. doi: 10.1021/jm070038n 17375905

[14] Zhu J, Mo J, Lin H, Chen Y, Sun H. The recent progress of isoxazole in medicinal chemistry. *Bioorg*. *Med*. *Chem*. 2018; 26: 3065–3075. doi: 10.1016/j.bmc.2018.05.013 29853341

[15] Sysak A, Obmińska-Mrukowicz B. Isoxazole ring as a useful scaffold in a search for new therapeutic agents. *Eur*. *J*. *Med*. *Chem*. 2017; 137: 292–309. doi: 10.1016/j.ejmech.2017.06.002 28605676

[16] Filali I, Bouajila J, Znati M, Bousejra-El Garah F, Jannet HB. Synthesis of new isoxazoline derivatives from harmine and evaluation of their anti-Alzheimer, anti-cancer and anti-inflammatory activities. *J*. *Enzyme Inhib*. *Med*. *Chem*. 2015; 30: 371–376. doi: 10.3109/14756366.2014.940932 25068731

[17] Mączyński M, Borska S, Mieszała K, Kocięba M, Zaczyńska E, Kochanowska I, et al. Synthesis, immunosuppressive properties, and mechanism of action of a new isoxazole derivative. *Molecules*. 2018; 23: 1545; doi: 10.3390/molecules23071545 29949951 PMC6099534

[18] Reddy KR, Rao PS, Dev GJ, Poornachandra Y, Kumar CG, Rao PSh, et al. Synthesis of novel 1, 2, 3-triazole/isoxazole functionalized 2H-chromene derivatives and their cytotoxic activity. *Bioorg*. *Med*. *Chem*. *Lett*. 2014; 24: 1661–1663. doi: 10.1016/j.bmcl.2014.02.069 24641975

[19] Loh B, Vozzolo L, Mok BJ, Lee ChCh, Fitzmaurice RJ, Caddick S, et al. Inhibition of HIV-1 replication by isoxazolidine and isoxazole sulfonamides. *Chem*. *Biol*. *Drug Des*. 2010; 75: 461–474. doi: 10.1111/j.1747-0285.2010.00956.x 20486932 PMC2917890

[20] Deng B-L, Cullen MD, Zhou Zh, Hartman TL, Buckheit RW Jr, Pannecouque Ch, et al. Synthesis and anti-HIV activity of new alkenyldiarylmethane (ADAM) non-nucleoside reverse transcriptase inhibitors (NNRTIs) incorporating benzoxazolone and benzisoxazole rings. *Bioorg*. *Med*. *Chem*. 2006; 14: 2366–2374. doi: 10.1016/j.bmc.2005.11.014 16321539

[21] Mao J, Yuan H, Wang Y, Wan B, Pieroni M, Huang Q, et al. From serendipity to rational antituberculosis drug discovery of mefoquine-isoxazole carboxylic acid esters. *J*. *Med*. *Chem*. 2009; 52: 6966–6978. doi: 10.1021/jm900340a 19863050

[22] Patrick DA, Bakunov SA, Bakunova SM, Kumar EVKS, Lombardy RJ, Jones SK, et al. Synthesis and in vitro antiprotozoal activities of dicationic 3,5-diphenylisoxazoles. *J*. *Med*. *Chem*. 2007; 50: 2468–2485. doi: 10.1021/jm0612867 17439202

[23] Ishioka T, Kubo A, Koiso Y, Nagasawa K, Itai A, Hashimoto Y. Novel non-steroidal/non-anilide type androgen antagonists with an isoxazolone moiety. *Bioorg*. *Med*. *Chem*. 2022; 10: 1555–1566. doi: 10.1016/s0968-0896(01)00421-7 11886817

[24] Ishioka T, Tanatani A, Nagasawa K, Hashimoto Y. Anti-androgens with full antagonistic activity toward human prostate tumor LNCaP cells with mutated androgen receptor. *Bioorg*. *Med*. *Chem*. *Lett*. 2003; 13: 2655–2658. doi: 10.1016/s0960-894x(03)00575-4 12873487

[25] Davidson JR, Giller EL, Zisook S, Overall JE. An efficacy study of isocarboxazid and placebo in depression, and its relationship to depressive nosology. *Arch*. *Gen*. *Psychiatry*. 1988; 45: 120–127. doi: 10.1001/archpsyc.1988.01800260024003 3276281

[26] Van Roon EN, Jansen TLTHA, Mourad L, Houtman PM, Bruyn GAW, Griep EN, et al. Leflunomide in active rheumatoid arthritis: a prospective study in daily practice, *Br*. *J*. *Clin*. *Pharmacol*. 2004; 57: 790–797. doi: 10.1111/j.1365-2125.2004.02075.x 15151525 PMC1884519

[27] Heiss JD, Argersinger DP, Theodore WH, Butman JA, Sato S, Khan OI. Convection-enhanced delivery of muscimol in patients with drug-resistant epilepsy. *Neurosurgery*. 2019; 85: E4–E15. doi: 10.1093/neuros/nyy480 30407567 PMC6704347

[28] Sathya A, Ramalingam S, Nagabalasubramaniyan PB, Karpagam J. Structure activity investigation, CT complex analysis and spectroscopic investigation on antibiotic drug; Sulfamethoxazole using quantum chemical calculations. *Chemical Physics Impact*. 2021; 3: 100031. doi: 10.1016/j.chphi.2021.100031

[29] Sahoo J, Kshiroda P, Sarangi N, Rout SK, Paidesetty SK. In silico investigation and biological evaluation of synthesized sulfamethoxazole derivatives. *Indian J*. *Pharm*. *Sci*. 2020; 82: 123–130. doi: 10.36468/pharmaceutical-sciences.629

[30] Sh Rostamizadeh, Daneshfar Z, Moghimi H. Synthesis of sulfamethoxazole and sulfabenzamide metal complexes; evaluation of their antibacterial activity. *Eur*. *J*. *Med*. *Chem*. 2019; 171: 364–371. doi: 10.1016/j.ejmech.2019.03.002 30928708

[31] Sattar Majeed H, Mohammed Fayyadh Kh. Synthesis and characterization of two types of heterogeneous rings derived from sulfamethoxazole and evaluation their bacterial activity. *IOP Conf*. *Ser*.: *Mater*. *Sci*. *Eng*. 2018; 454: 012041. doi: 10.1088/1757-899X/454/1/012041

[32] Alsahib SA. Characterization and biological activity of some new derivatives derived from sulfamethoxazole compound. *Baghdad Sci*. *J*. 2020; 17: 0471. doi: 10.21123/bsj.2020.17.2.0471

[33] Chohan ZH, Shad HA, Toupet L, Hadda TB, Akkurt M. Structure of a new bioactive agent containing combined antibacterial and antifungal pharmacophore sites: 4-{[(E)-(5-Bromo-2-hydroxyphenyl)methylidene]amino}-N-(5-methyl-1,2-oxazol-3-yl)benzenesulfonamide. *J*. *Chem*. *Crystallogr*. 2011; 41: 159–162. doi: 10.1007/s10870-010-9856-x

[34] Marwaha S, Uvell H, Salin O, Lindgren AEG, Silver J, Elofsson M, et al. N-Acylated derivatives of sulfamethoxazole and sulfafurazole inhibit intracellular growth of Chlamydia trachomatis. *Antimicrob*. *Agents Chemother*. 2014; 58: 2968–2971. doi: 10.1128/AAC.02015-13 24566180 PMC3993265

[35] Moghaddam VA, Kasmaeifar V, Mahmoodi Z, Ghafouri H, Saberi O, Mohammadi A. A novel sulfamethoxazole derivative as an inhibitory agent against HSP70: A combination of computational with in vitro studies. *Int*. *J*. *Biol*. *Macromol*. 2021; 189: 194–205. doi: 10.1016/j.ijbiomac.2021.08.128 34428485

[36] Akili S, Hadda DB, Bitar Y, Balash A, Chehna MF. Design, synthesis and characterization of novel sulfonamides derivatives as anticancer agent targeting EGFR TK, and development of new methods of synthesis by microwave irradiation. *Int*. *J*. *Org*. *Chem*. 2021; 11: 199–223. doi: 10.4236/ijoc.2021.114014

[37] Krátký M, Konečná K, Janoušek J, Janďourek O, Maixnerová J, Kalivodová S, et al. Sulfonamide-salicylaldehyde imines active against methicillin- and trimethoprim/sulfonamide-resistant Staphylococci. *Future Med*. *Chem*. 2021; 13:1945–1962. doi: 10.4155/fmc-2021-0169 34633218

[38] Hassan AU, Sumrra SH, Raza MA, Zubair M, Zafar MN, Mughal EU, et al. Design, facile synthesis, spectroscopic characterization, and medicinal probing of metal-based new sulfonamide drugs: A theoretical and spectral study. *Appl*. *Organomet*. *Chem*. 2020. doi: 10.1002/aoc.6054

[39] Cordenonsi Bonez P, Agertt VA, Rossi GG, dos Santos Siqueira F, Siqueira JD, Marques LL, et al. Sulfonamides complexed with metals as mycobacterial biofilms inhibitors. *J*. *Clin*. *Tuberc*. *Other Mycobact*. *Dis*. 2021; 23: 100217. doi: 10.1016/j.jctube.2021.100217 33869806 PMC8044702

[40] Balandis B, Šimkūnas T, Paketurytė-Latvė V, Michailovienė V, Mickevičiūtė A, Manakova E, et al. Beta and gamma amino acid-substituted benzenesulfonamides as inhibitors of human carbonic anhydrases. *Pharmaceuticals*. 2022; 15: 1–31. doi: 10.3390/ph15040477 35455474 PMC9033141

[41] Urbelytė L, Bagdonas M, Grybaitė B, Vaickelionienė R, Mickevičiūtė A, Michailovienė V, et al. Design and synthesis of hydrazone-bearing benzenesulfonamides as carbonic anhydrase VB inhibitors. *ChemistrySelect*. 2021; 6: 13506–13513. doi: 10.1002/slct.202103636

[42] Balandis B, Mickevičius V, Petrikaitė V. Exploration of benzenesulfonamide-bearing Imidazole derivatives activity in triple-negative breast cancer and melanoma 2D and 3D cell cultures. *Pharmaceuticals*. 2021; 14: 1–14. doi: 10.3390/ph14111158 34832940 PMC8625351

[43] Balandis B, Ivanauskaitė G, Smirnovienė J, Kantminienė K, Matulis D, Mickevičius V, et al. Synthesis and structure-affinity relationship of chlorinated pyrrolidinone-bearing benzenesulfonamides as human carbonic anhydrase inhibitors. *Bioorg*. *Chem*. 2020; 97: 1–12. doi: 10.1016/j.bioorg.2020.103658 32088419

[44] Vaškevičienė I, Paketurytė V, Pajanok N, Žukauskas Š, Sapijanskaitė B, Kantminienė K, et al. Pyrrolidinone-bearing methylated and halogenated benzenesulfonamides as inhibitors of carbonic anhydrases. *Bioorg*. *Med*. *Chem*. 2019; 27: 322–337. doi: 10.1016/j.bmc.2018.12.011 30553625

[45] Vaškevičienė I, Paketurytė V, Zubrienė A, Kantminienė K, Mickevičius V, Matulis D. N-Sulfamoylphenyl- and N-sulfamoylphenyl-N-thiazolyl-β-alanines and their derivatives as inhibitors of human carbonic anhydrases. *Bioorg*. *Chem*. 2017; 75: 16–29. doi: 10.1016/j.bioorg.2017.08.017 28888097

[46] Čeponytė U, Paškevičiūtė M, Petrikaitė V. Comparison of NSAIDs activity in COX-2 expressing and non-expressing 2D and 3D pancreatic cancer cell cultures. *Cancer Manag Res*. 2018; 10: 1543–1551. doi: 10.2147/CMAR.S163747 29942156 PMC6007190

[47] Petrikaitė V, Tarasevičius E, Pavilonis A. New thiazolidones-4 with sulfamethizole-2 substituent as potential antifungal and antimicrobial preparations. *Biologija*. 2007; 53: 45–50.

[48] Parašotas I, Urbonavičiūtė E, Anusevičius K, Tumosienė I, Jonuškienė I, Kantminienė K, et al. Synthesis and biological evaluation of novel di- and trisubstituted thiazole derivatives. *Heterocycles*. 2017; 94: 1074–1097. doi: 10.3987/COM-17-13714

[49] Tumosienė I, Kantminienė K, Klevinskas A, Petrikaitė V, Jonuškienė I, Mickevičius V. Antioxidant and anticancer activity of novel derivatives of 3– [(4-methoxyphenyl)amino]propane-hydrazide. *Molecules*. 2020; 25: 2980. doi: 10.3390/molecules25132980 32610506 PMC7412228

[50] Tisovský P, Csicsai K, Donovalová J, Šandrik R, Sokolík R, Gáplovský A. Effect of a = X-NH-fragment, (X = C, N), on Z/E isomerization and ON/OFF functionality of isatin arylhydrazones, ((arylamino)methylene)indolin-2-ones and their anions. *Molecules*. 2020; 25: 3082. doi: 10.3390/molecules25133082 32640761 PMC7412119

[51] Tumosienė I, Peleckis A, Jonuškienė I, Vaickelionienė R, Kantminienė K, Šiugždaitė J, et al. Synthesis of novel 1,2- and 2-substituted benzimidazoles with high antibacterial and antioxidant activity. *Monatsh*. *Chem*. 2018; 149: 577–594, doi: 10.1007/s00706-017-2066-x

[52] Han Mİ, Bekçi H, Uba AI, Yıldırım Y, Karasulu E, Cumaoğlu A, et al. Synthesis, molecular modeling, in vivo study, and anticancer activity of 1,2,4-triazole containing hydrazide–hydrazones derived from (S)-naproxen. *Arch*. *Pharm*. *Chem*. *Life Sci*. 2019; e1800365. doi: 10.1002/ardp.201800365 31115928

[53] Jo Y, Choi N, Kim K, Koo H-J, Choi J, Kim HN. Chemoresistance of cancer cells: requirements of Tumor microenvironment-mimicking in vitro models in anti-cancer drug development. *Theranostics*. 2018; 8: 5259–5275. doi: 10.7150/thno.29098 30555545 PMC6276092

[54] Barbosa MAG, Xavier CPR, Pereira RF, Petrikaitė V, Vasconcelos MH. 3D cell culture models as recapitulators of the Tumor microenvironment for the screening of anti-cancer drugs. *Cancers*. 2021; 14: 190. doi: 10.3390/cancers14010190 35008353 PMC8749977

[55] Gray RE, Harris GT. Renal cell carcinoma: diagnosis and management. *Am Fam Physician*. 2019; 99: 179–184. 30702258

[56] Makhov P, Joshi S, Ghatalia P, Kutikov A, Uzzo RG, Kolenko V.M. Resistance to systemic therapies in clear cell renal cell carcinoma: mechanisms and management strategies. *Mol Cancer Ther*. 2018; 17: 1355–1364. doi: 10.1158/1535-7163.MCT-17-1299 29967214 PMC6034114

[57] Mizumoto A, Yamamoto K, Nakayama Y, Takara K, Nakagawa T, Hirano T, et al. Induction of epithelial-mesenchymal transition via activation of epidermal growth factor receptor contributes to sunitinib resistance in human renal cell carcinoma cell lines. *J Pharmacol Exp Ther*. 2015; 355: 152–158. doi: 10.1124/jpet.115.226639 26306766

[58] Damaraju VL, Kuzma M, Mowles D, Cass CE, Sawyer M.B. Interactions of multitargeted kinase inhibitors and nucleoside drugs: achilles heel of combination therapy? *Mol Cancer Ther*. 2015; 14: 236–245. doi: 10.1158/1535-7163.MCT-14-0337 25519698

[59] Rawla P. Epidemiology of prostate cancer. *World J Oncol*. 2019; 10: 63–89. doi: 10.14740/wjon1191 31068988 PMC6497009

[60] Nevedomskaya E, Baumgart SJ, Haendler B. Recent advances in prostate cancer treatment and drug discovery. *Int J Mol Sci*. 2018; 19: 1359. doi: 10.3390/ijms19051359 29734647 PMC5983695

[61] Zhou S, Dai Z, Wang L, Gao X, Yang L, Wang Z, et al. MET inhibition enhances PARP inhibitor efficacy in castration-resistant prostate cancer by suppressing the ATM/ATR and PI3K/AKT pathways. *Journal of Cellular and Molecular Medicine*. 2021; 25: 11157–11169. doi: 10.1111/jcmm.17037 34761497 PMC8650038

[62] Hossain O, Rahman E, Roy H, Azam MdS, Ahmed S. Synthesis, characterization, and comparative assessment of antimicrobial properties and cytotoxicity of graphene-, silver-, and zinc-based nanomaterials. *Analytical Science Advances*. 2022; 3: 54–63. doi: 10.1002/ansa.202100041 PMC10989569 38716059

[63] Leite-Sampaio NF, Gondim CNFL, Martins RAA, Siyadatpanah A, Norouzi R, Kim B, et al. 2022. Potentiation of the activity of antibiotics against ATCC and MDR bacterial strains with (+)-α-Pinene and (-)-Borneol. *BioMed Research International*. 2022; e8217380. doi: 10.1155/2022/8217380 35663042 PMC9159878

[64] Sulfonamides Scholar E., in: Enna SJ, Bylund DB (Eds.), XPharm: The Comprehensive Pharmacology Reference, Elsevier, New York, 2007: pp. 1–4.

[65] Le VVH, Rakonjac J. Nitrofurans: Revival of an “old” drug class in the fight against antibiotic resistance. *PLOS Pathogens*. 2021;17: e1009663. doi: 10.1371/journal.ppat.1009663 34237108 PMC8266087

