# Supplementary material for: Synthesis of novel sulphamethoxazole derivatives and exploration of their anticancer and antimicrobial properties
Source: PLoS One. 2023 Mar 23;18(3):e0283289. doi: 10.1371/journal.pone.0283289 (PMC10035904; doi:10.1371/journal.pone.0283289)
Supplement: S1 File — (DOCX) [file pone.0283289.s001.docx]

**Additional ^1^H, ^13^C NMR and MS information**


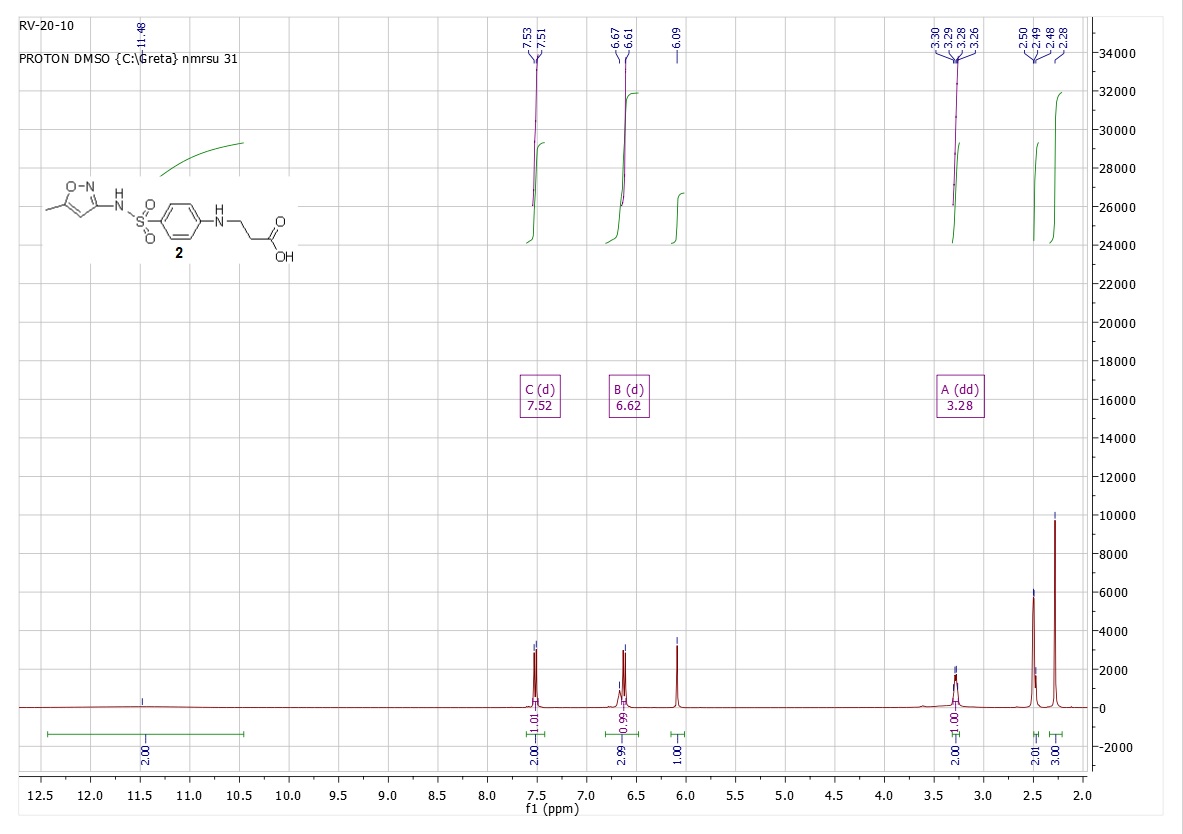


**S1 Fig.** ^1^H NMR of compound **2** at 400 MHz (DMSO-d_6_)


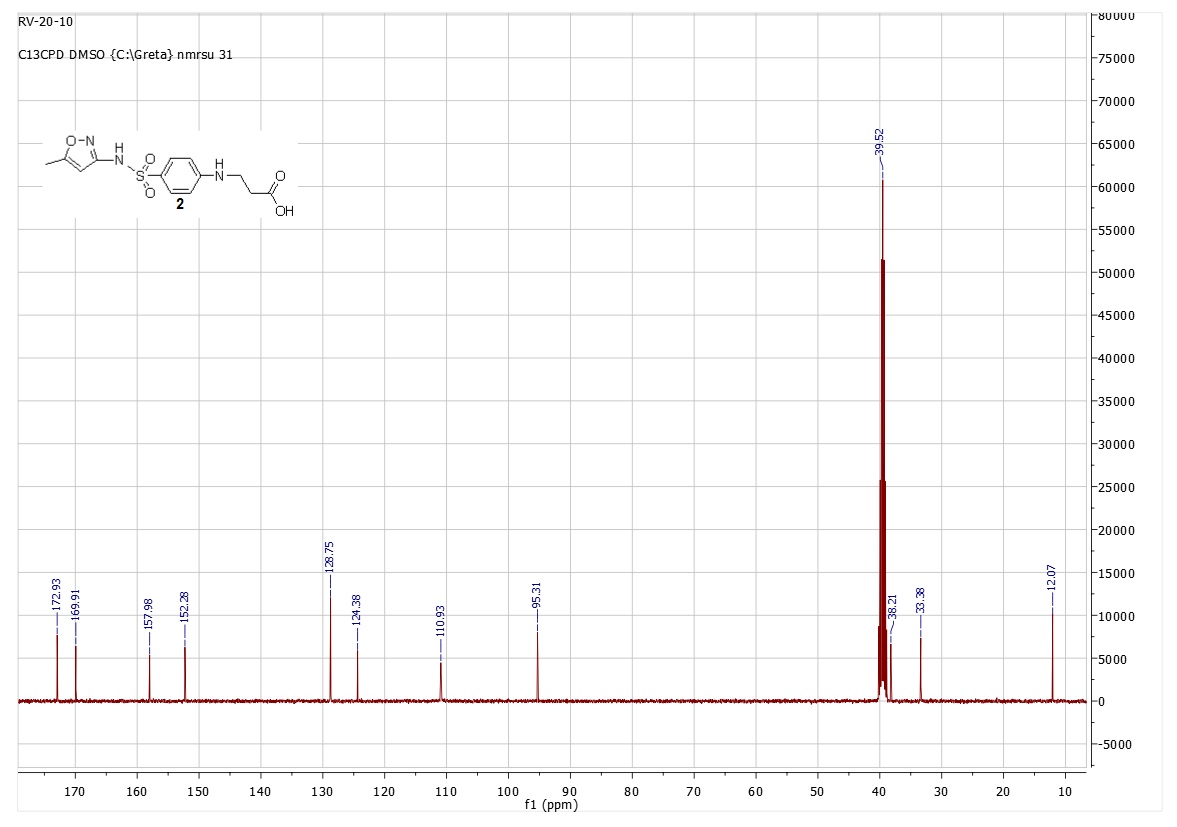


**S2 Fig.** ^13^C NMR of compound **2** at 101 MHz (DMSO-d_6_)


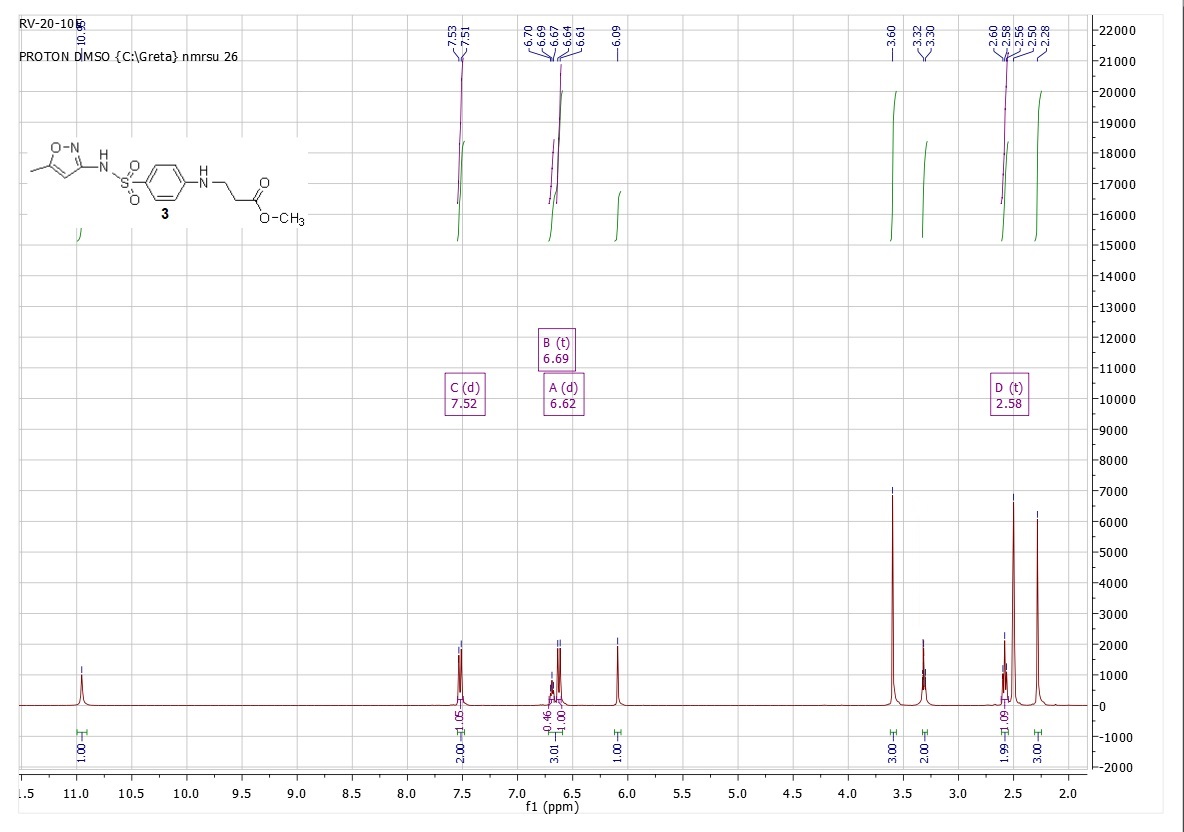


**S3 Fig.** ^1^H NMR of compound **3** at 400 MHz (DMSO-d_6_)


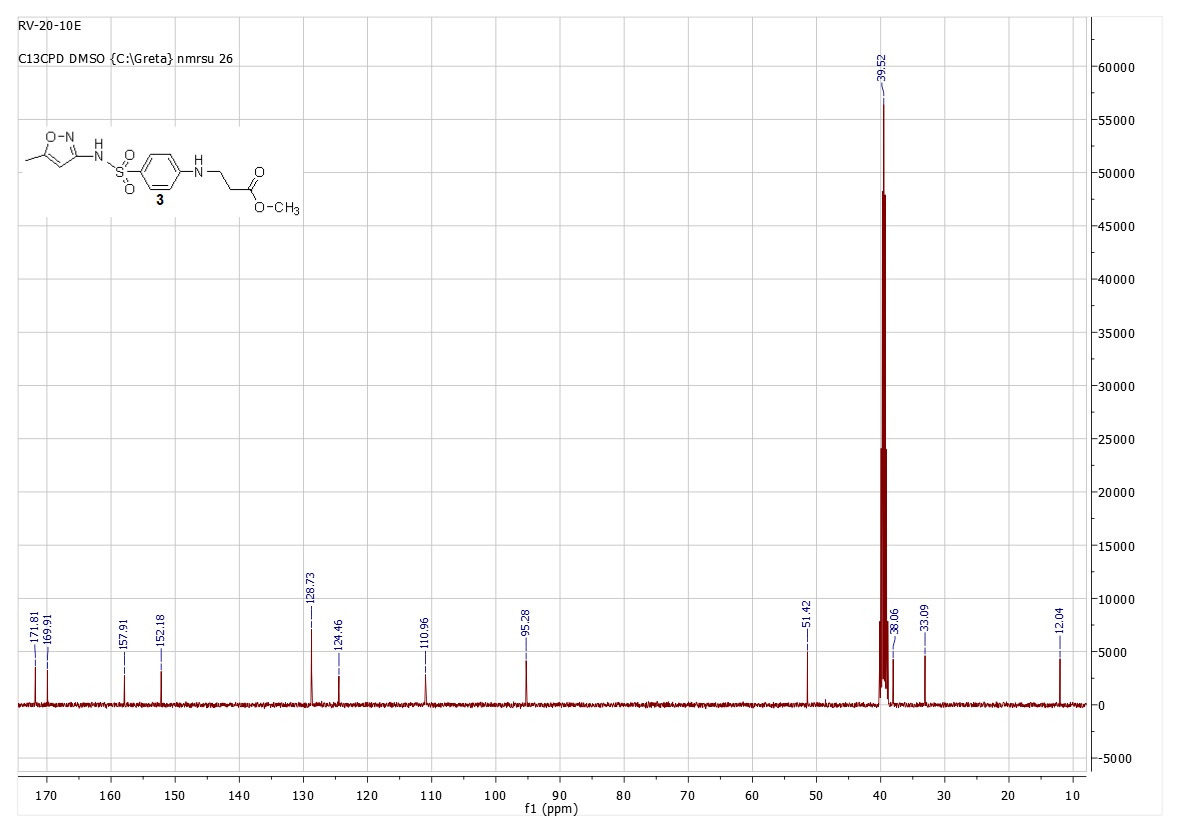


**S4 Fig.** ^13^C NMR of compound **3** at 101 MHz (DMSO-d_6_)


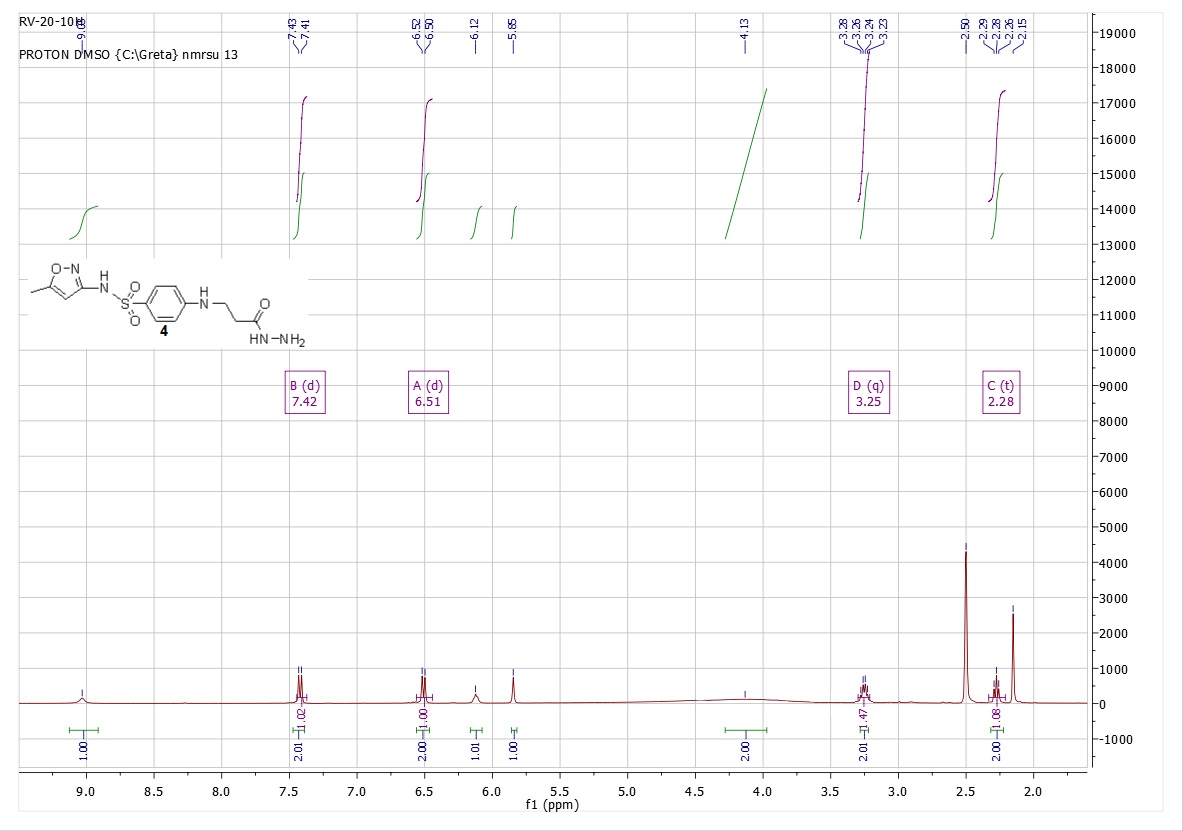


**S5 Fig.** ^1^H NMR of compound **4** at 400 MHz (DMSO-d_6_)


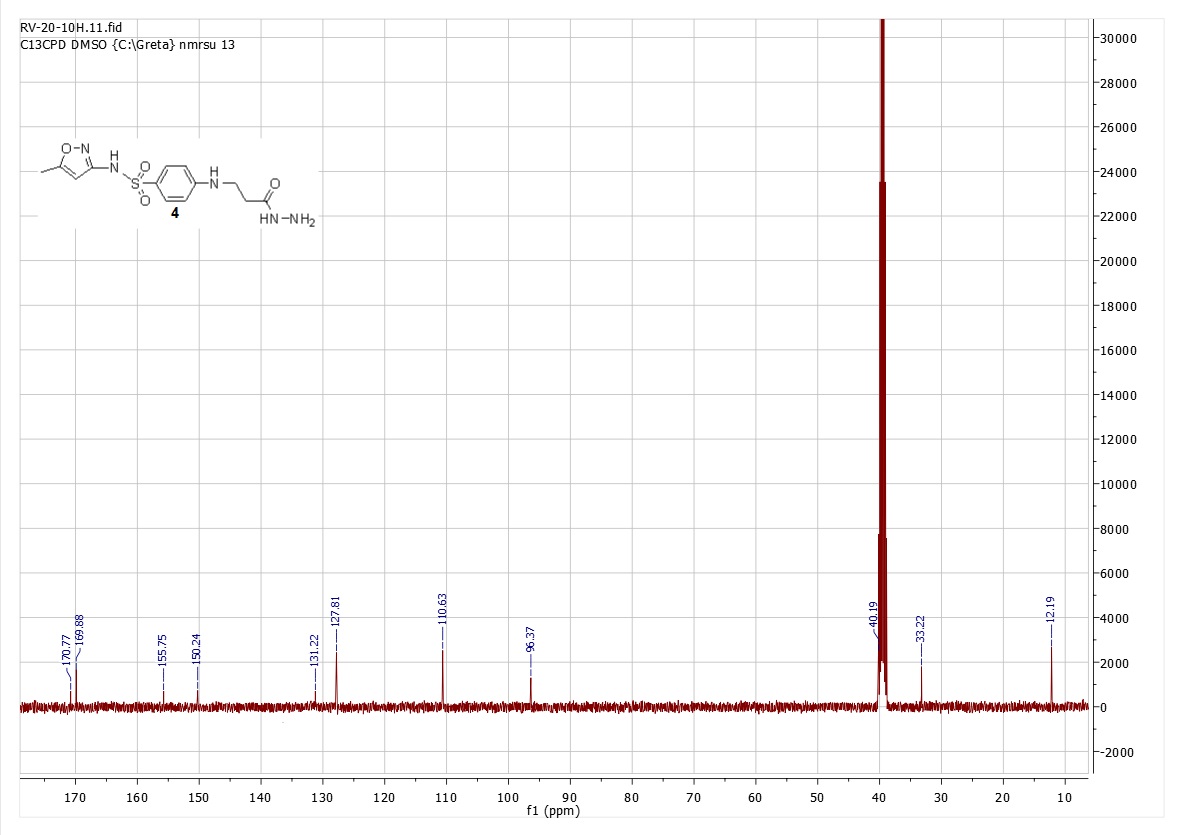


**S6 Fig.** ^13^C NMR of compound **4** at 101 MHz (DMSO-d_6_)


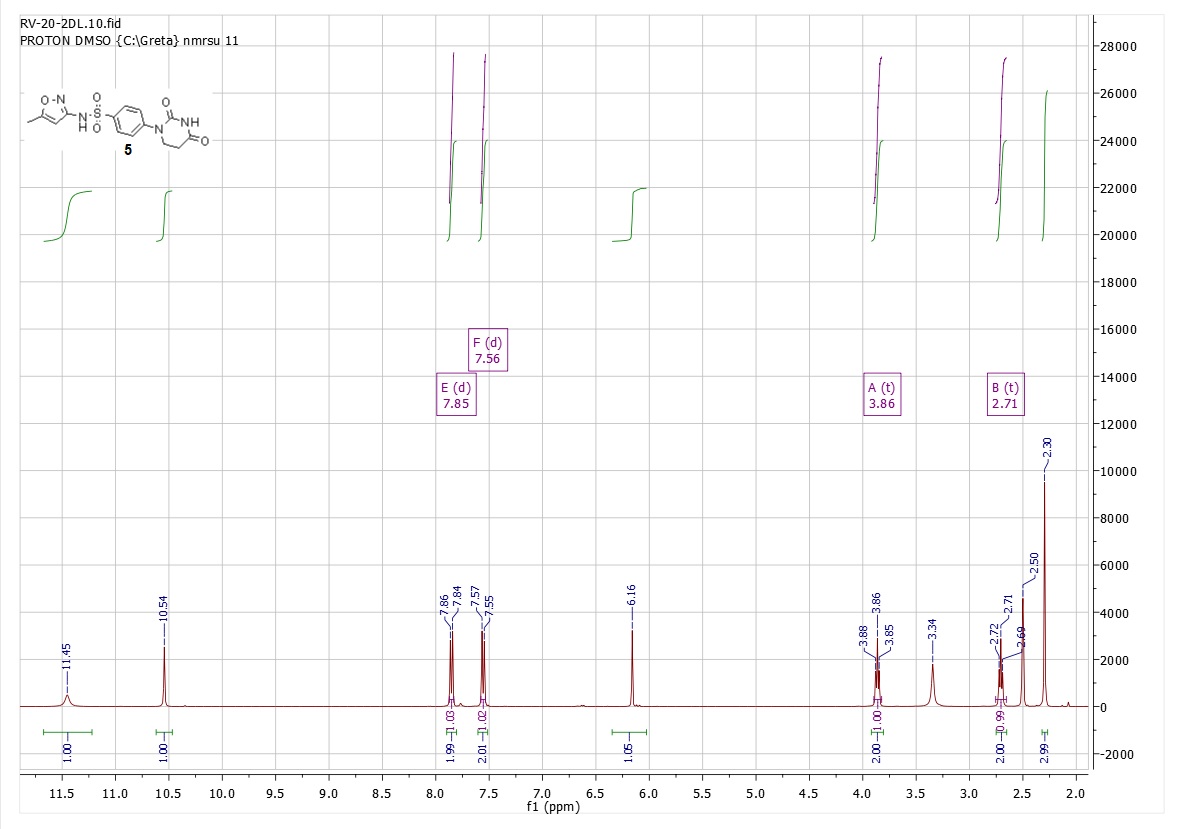


**S7 Fig.** ^1^H NMR of compound **5** at 400 MHz (DMSO-d_6_)


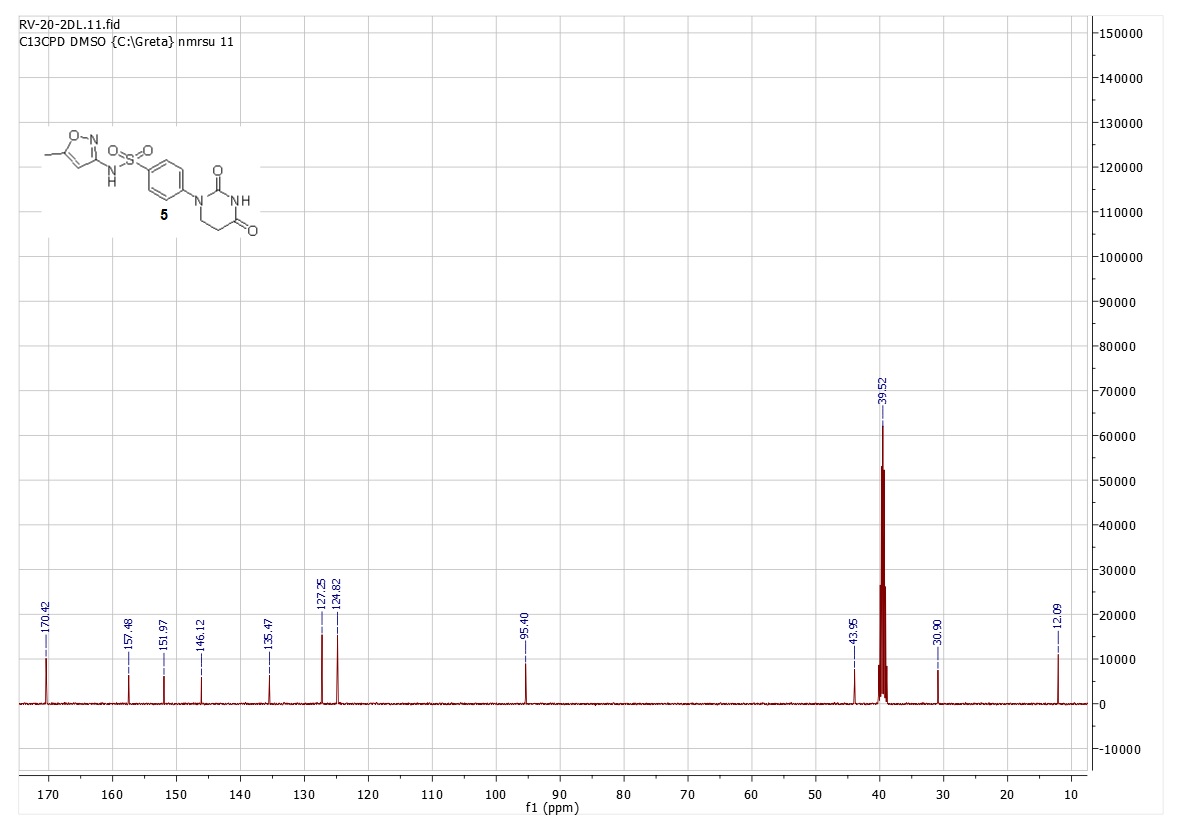


**S8 Fig.** ^13^C NMR of compound **5** at 101 MHz (DMSO-d_6_)

**
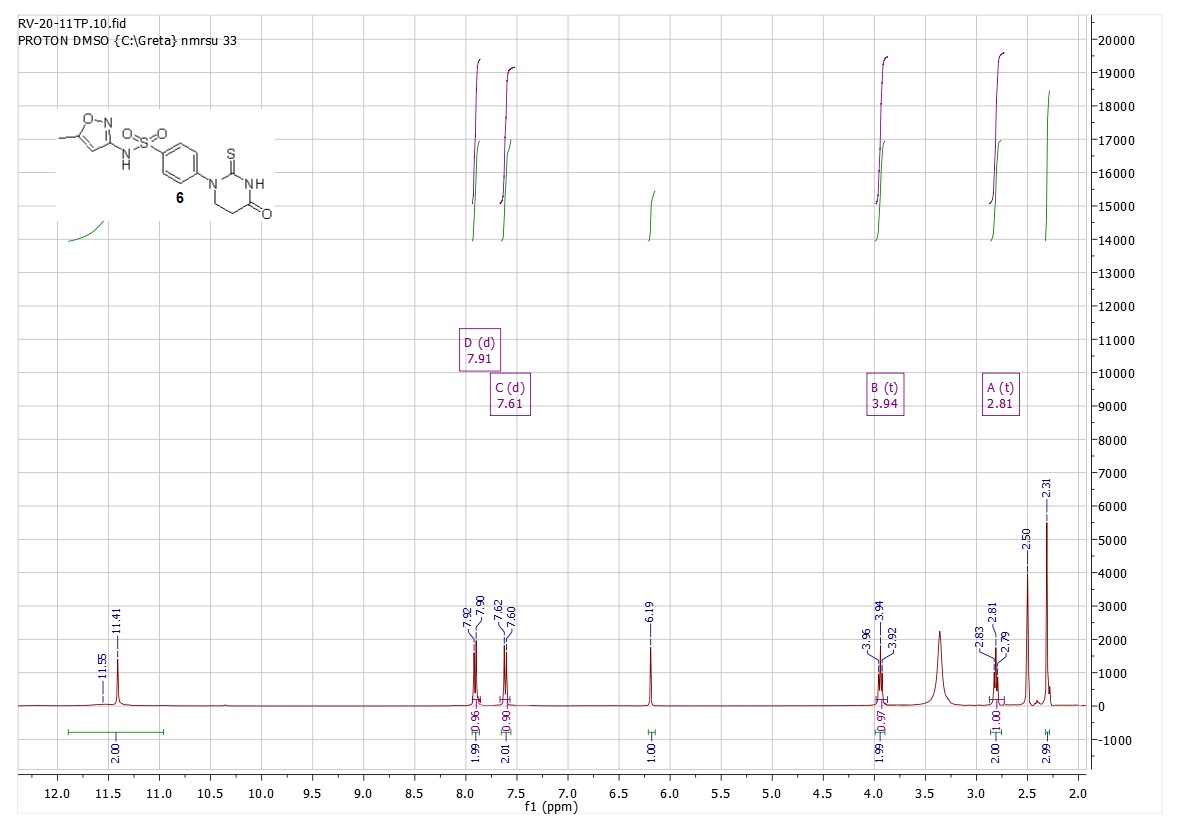
**

**S9 Fig.** ^1^H NMR of compound **6** at 400 MHz (DMSO-d_6_)


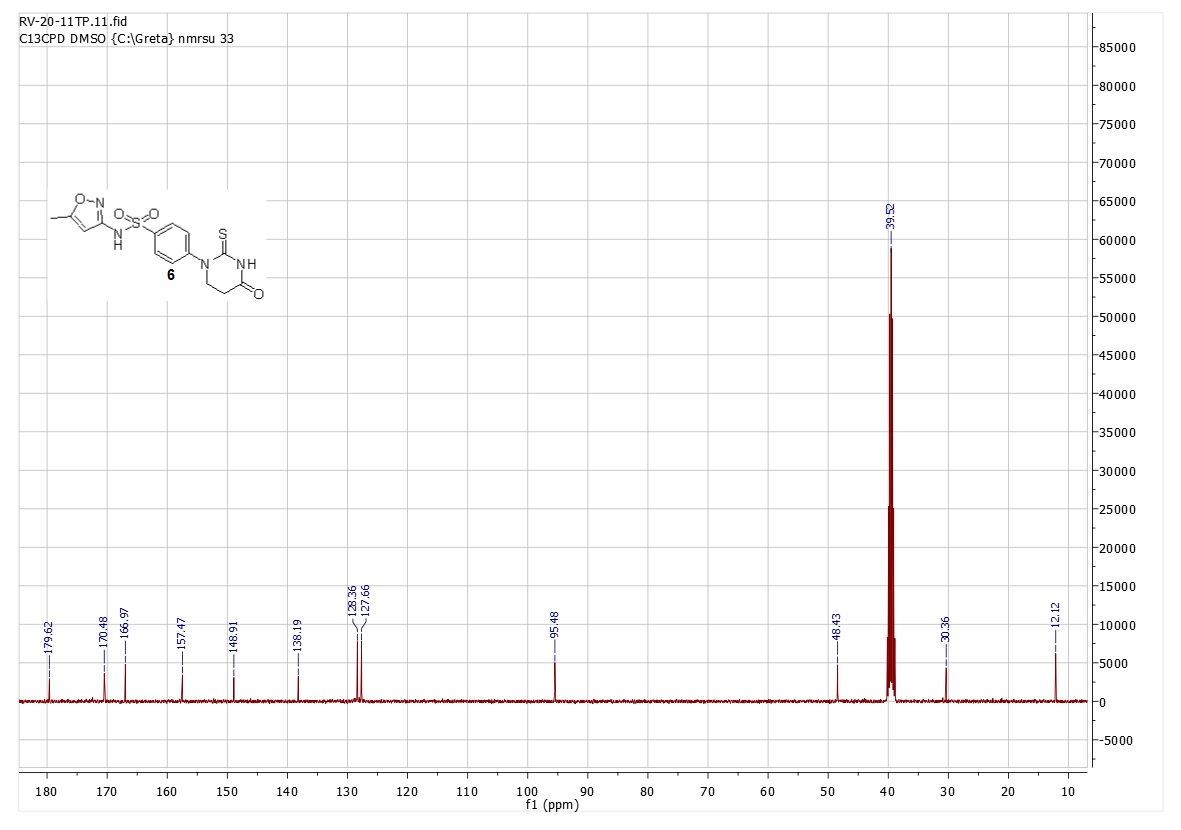


**S10 Fig.** ^13^C NMR of compound **6** at 101 MHz (DMSO-d_6_)


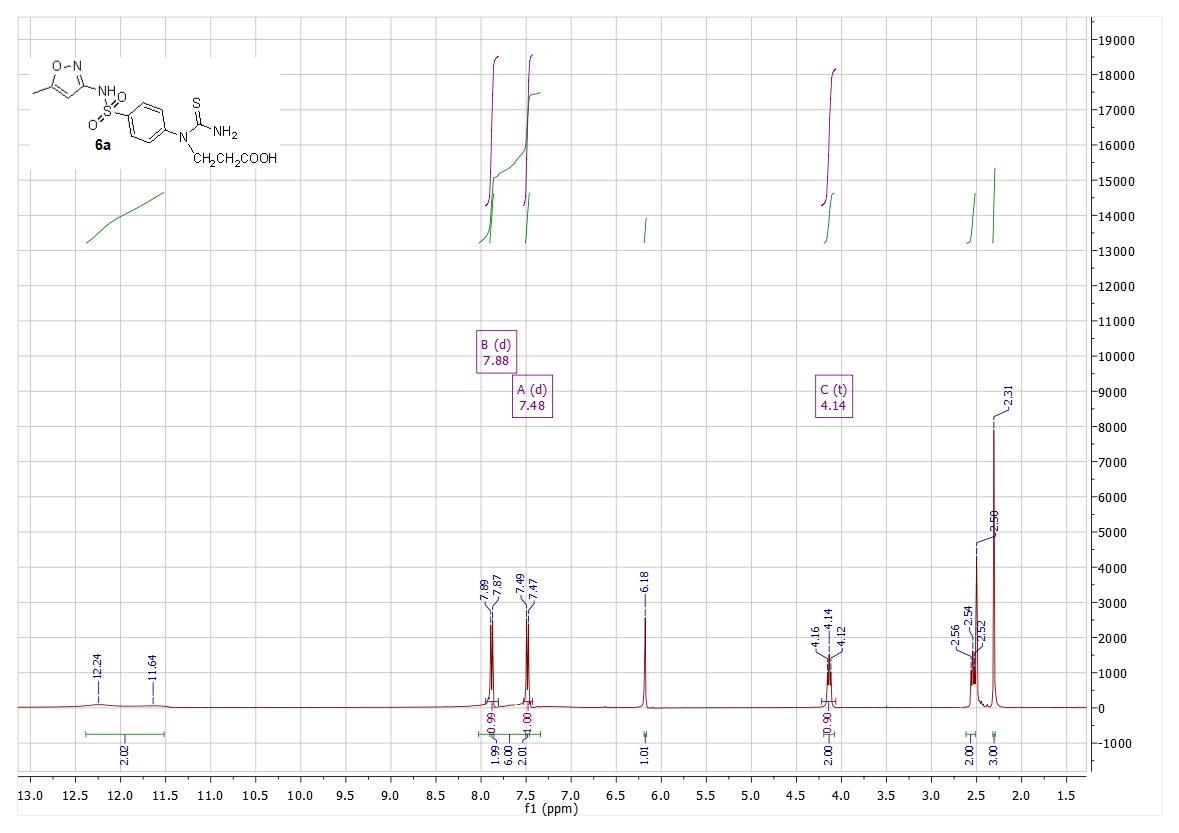


**S11 Fig.** ^1^H NMR of compound **6a** at 400 MHz (DMSO-d_6_)


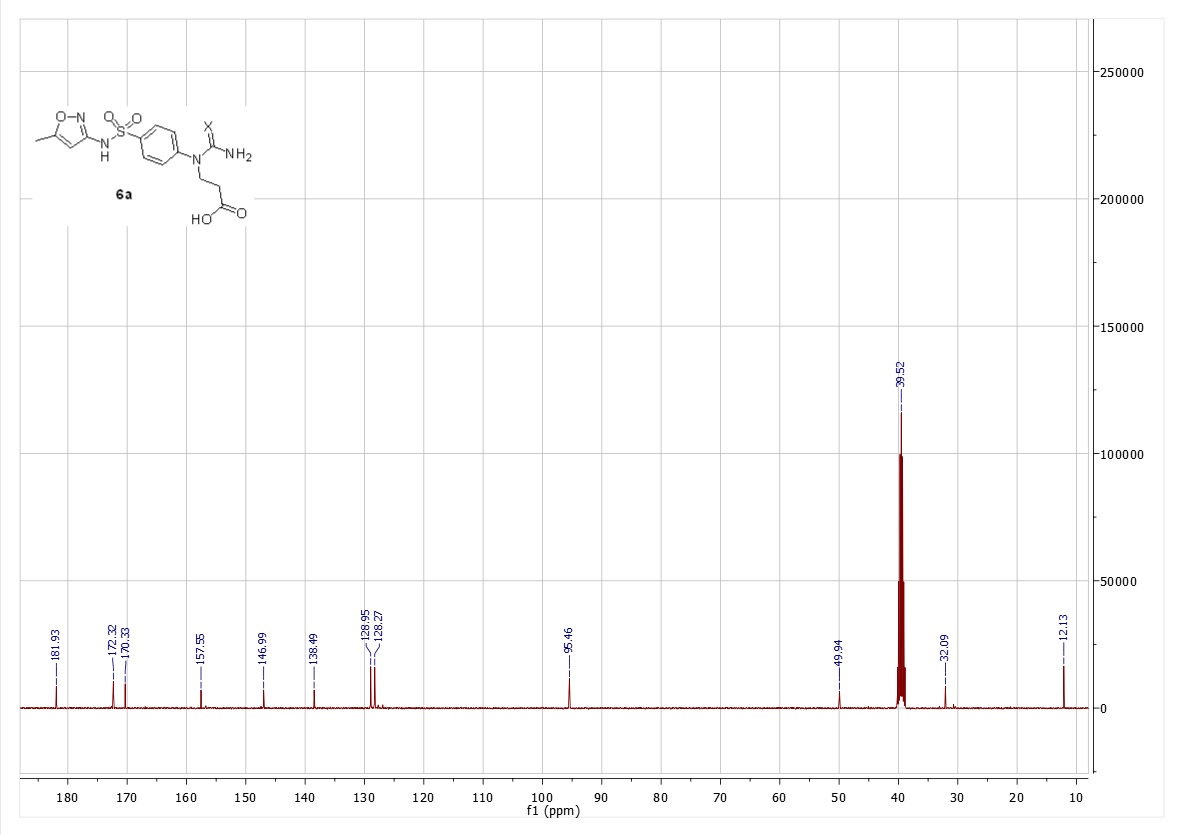


**S12 Fig.** ^13^C NMR of compound **6a** at 101 MHz (DMSO-d_6_)


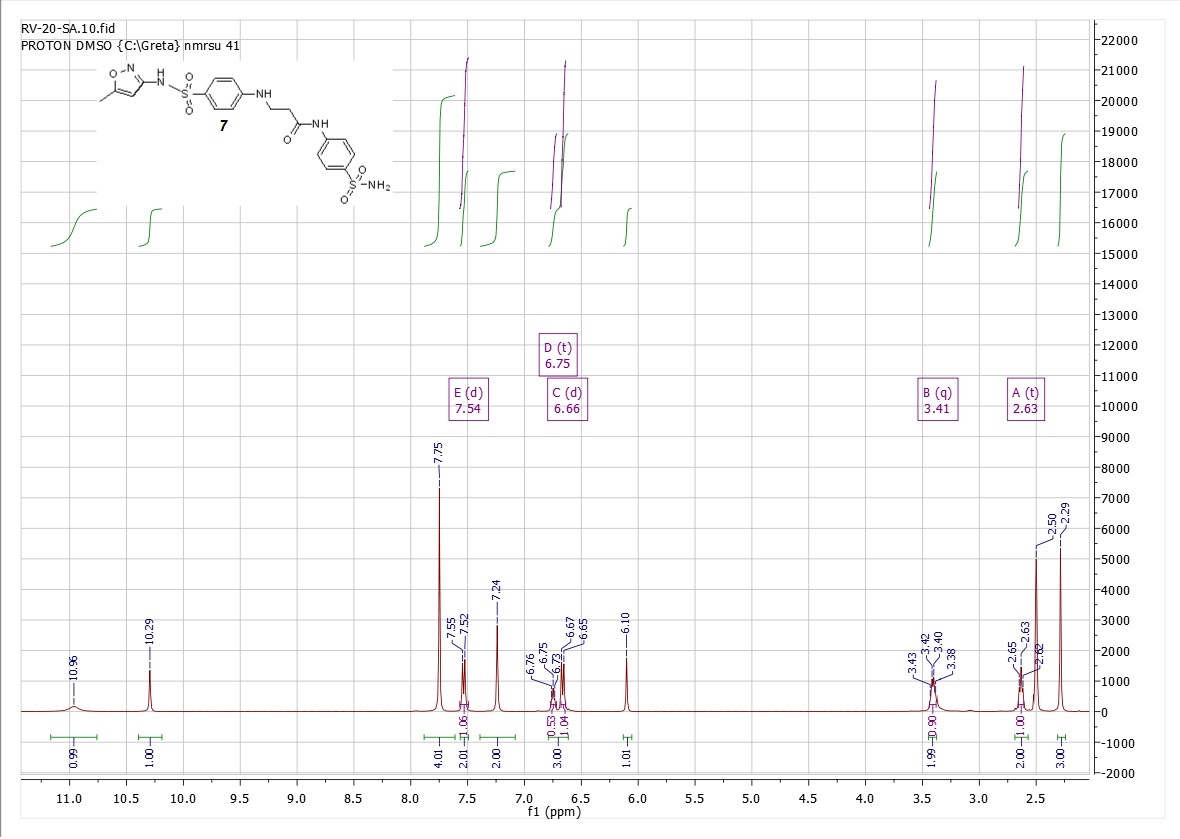


**S13 Fig.** ^1^H NMR of compound **7** at 400 MHz (DMSO-d_6_)


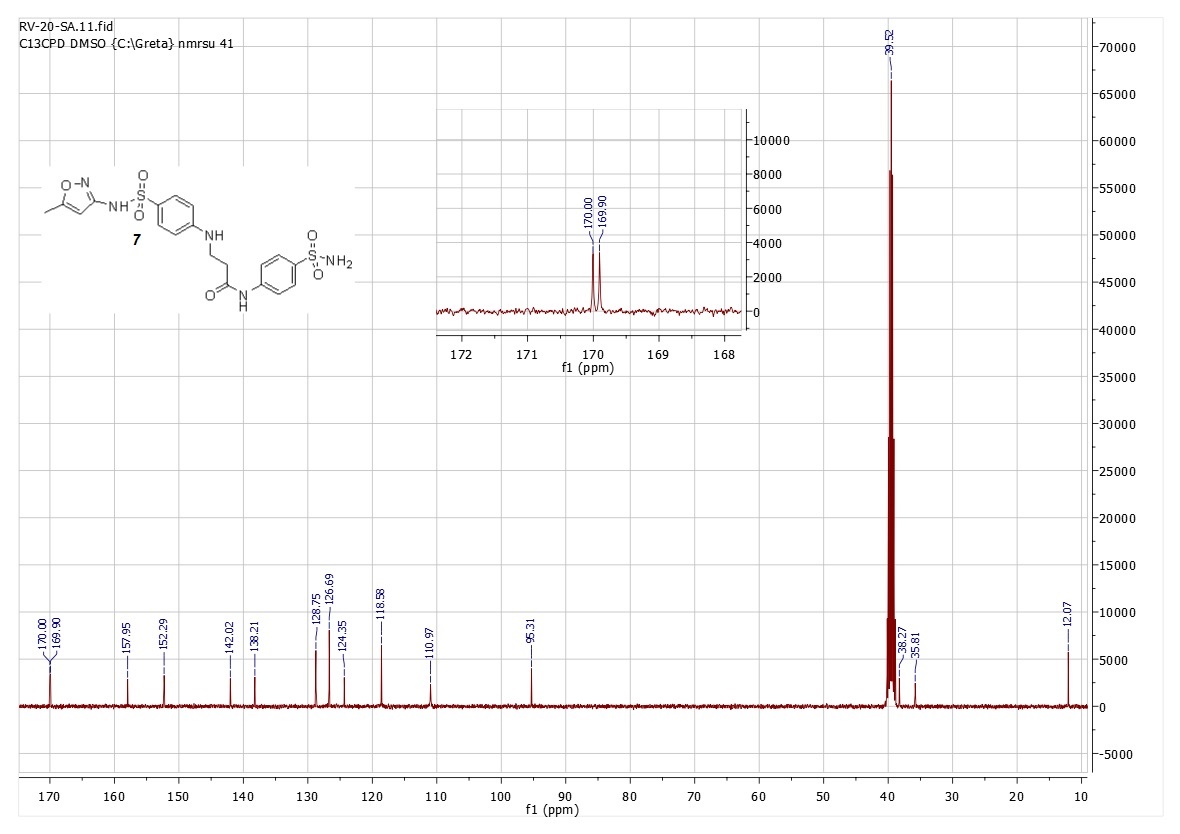


**S14 Fig.** ^13^C NMR of compound **7** at 101 MHz (DMSO-d_6_)

**
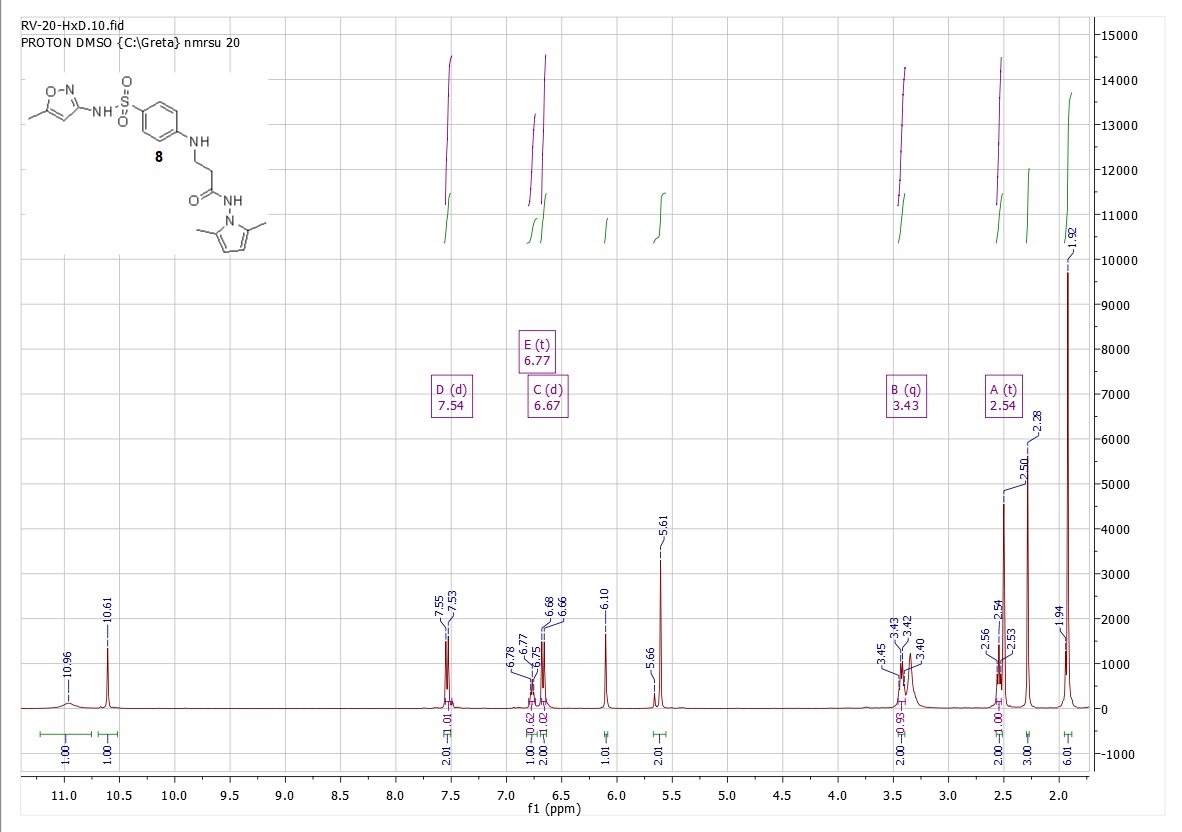
**

**S15 Fig.** ^1^H NMR of compound **8** at 400 MHz (DMSO-d_6_)

**
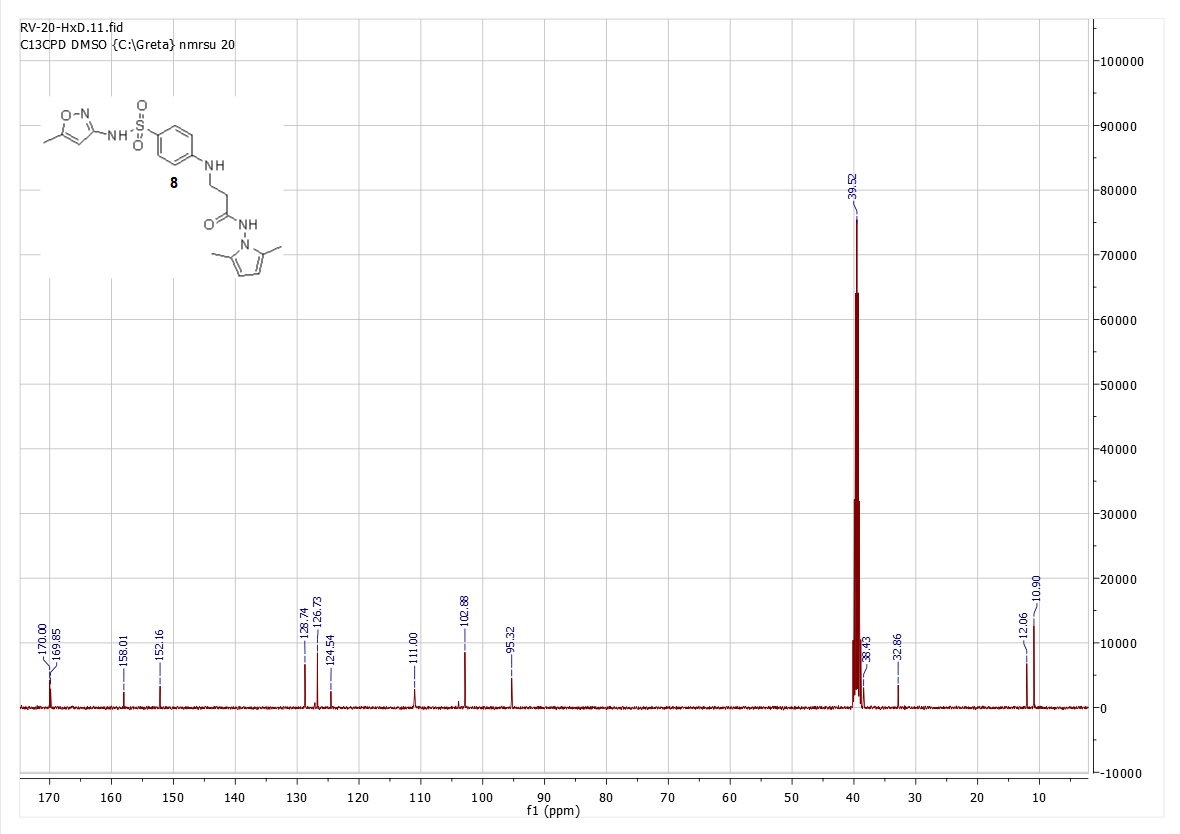
**

**S16 Fig.** ^13^C NMR of compound **8** at 101 MHz (DMSO-d_6_)


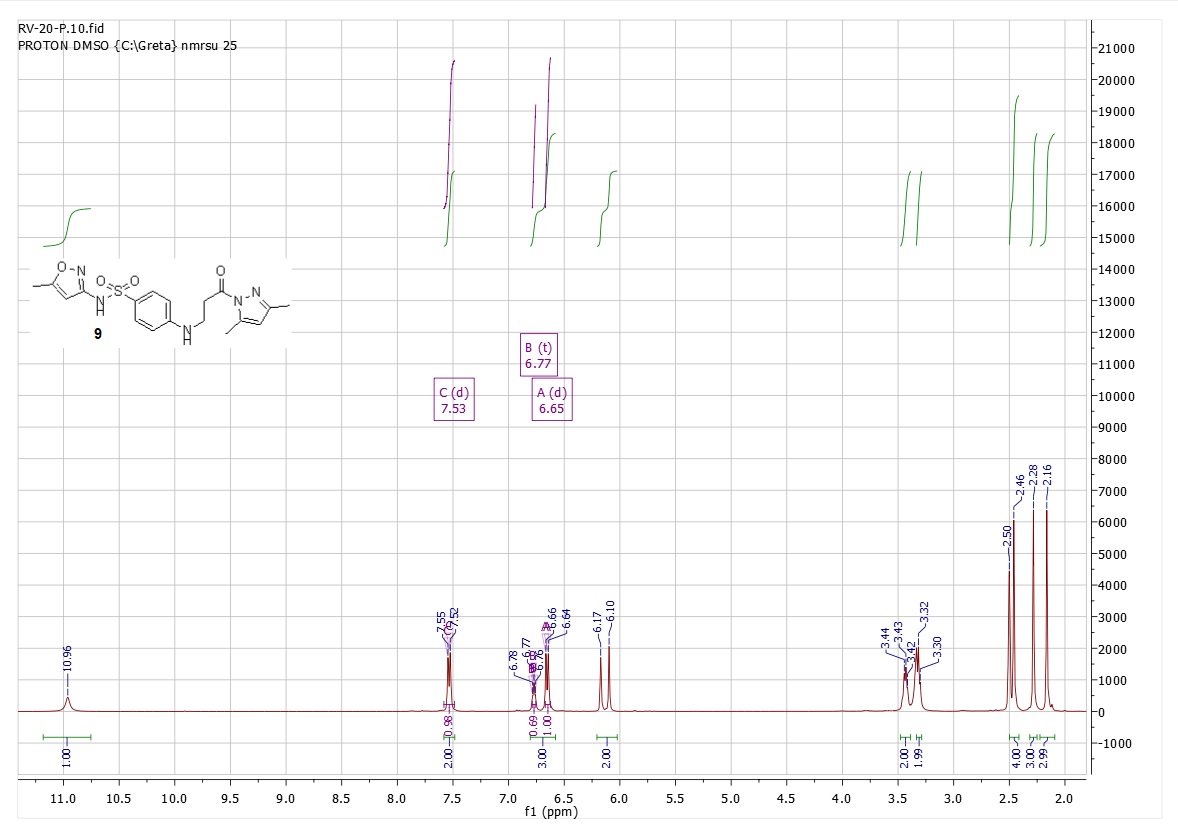


**S17 Fig.** ^1^H NMR of compound **9** at 400 MHz (DMSO-d_6_)


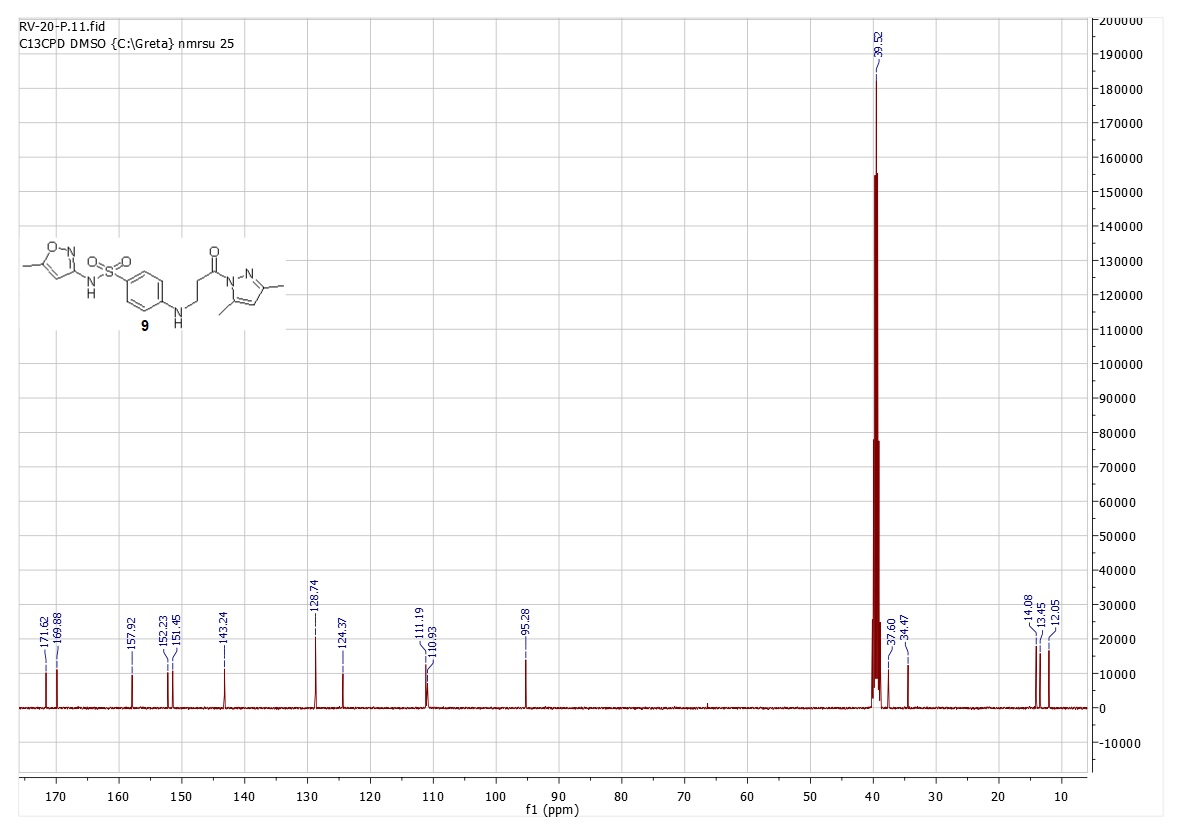


**S18 Fig.** ^13^C NMR of compound **9** at 101 MHz (DMSO-d_6_)


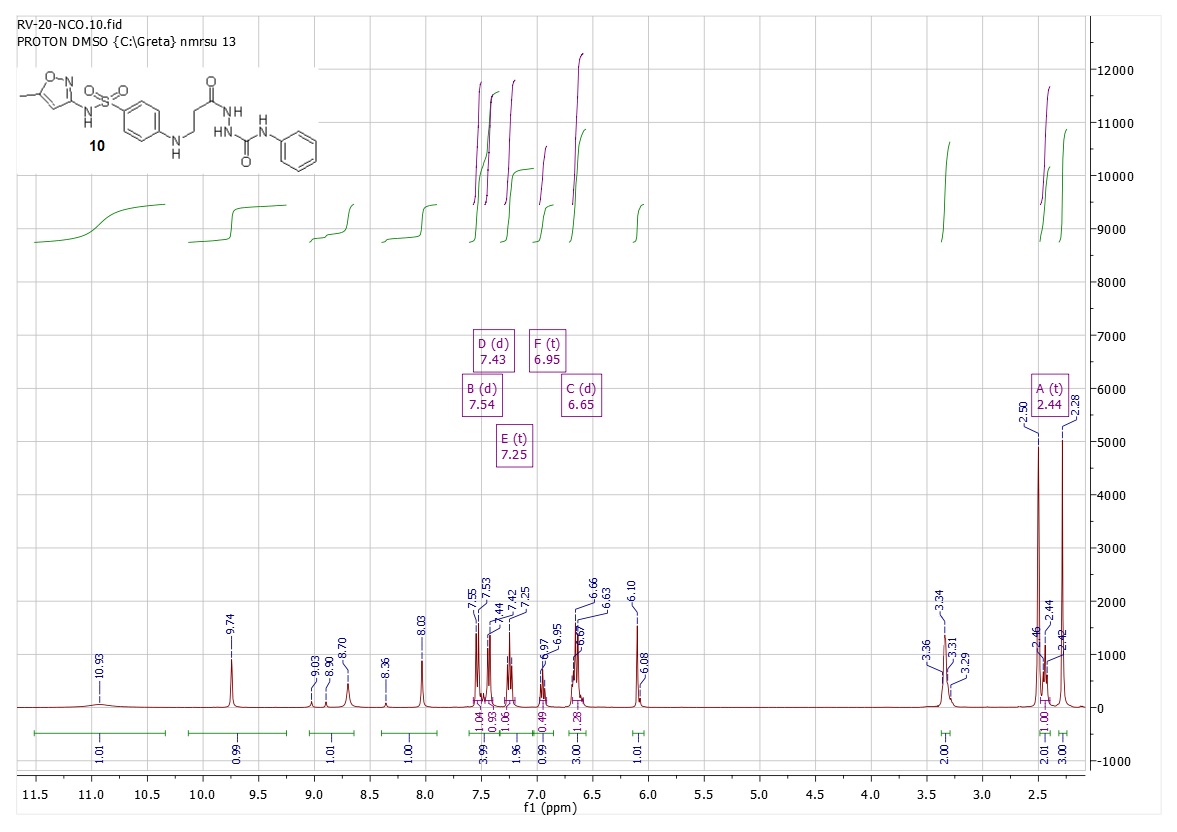


**S19 Fig.** ^1^H NMR of compound **10** at 400 MHz (DMSO-d_6_)

**
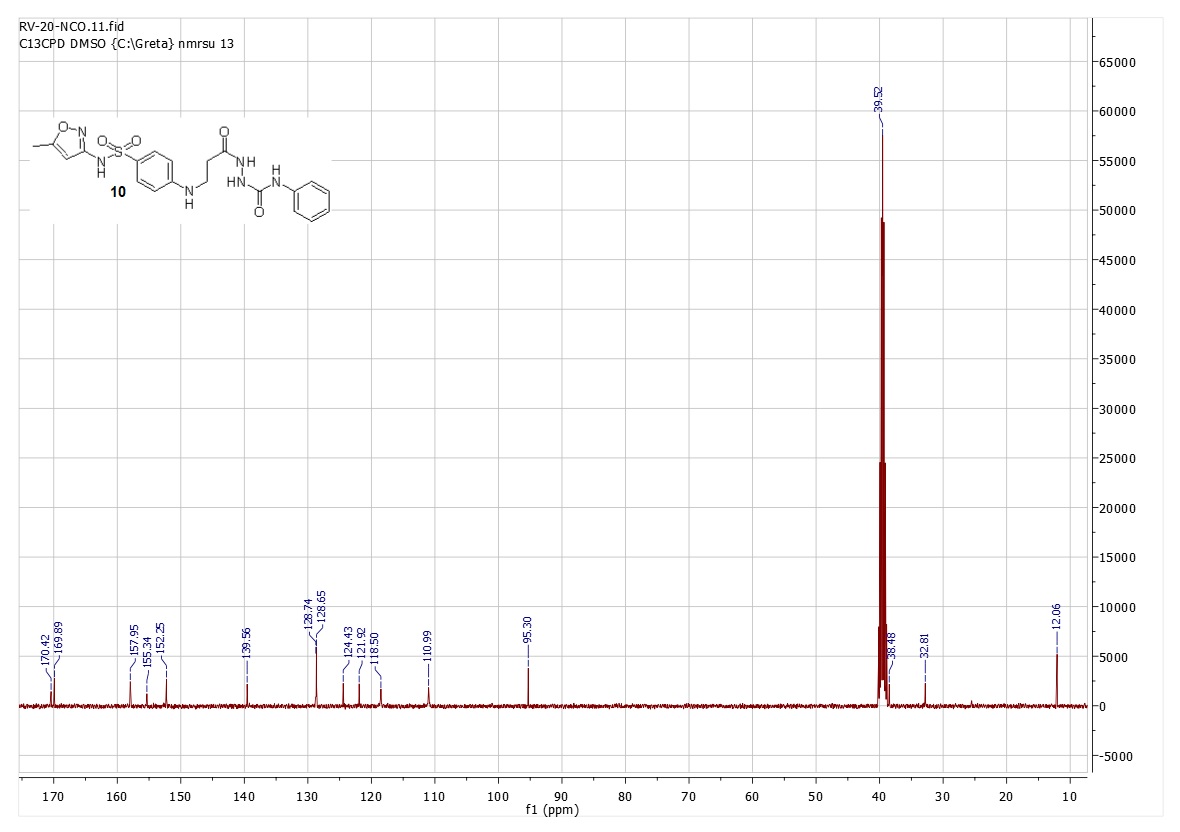
**

**S20 Fig.** ^13^C NMR of compound **10** at 101 MHz (DMSO-d_6_)


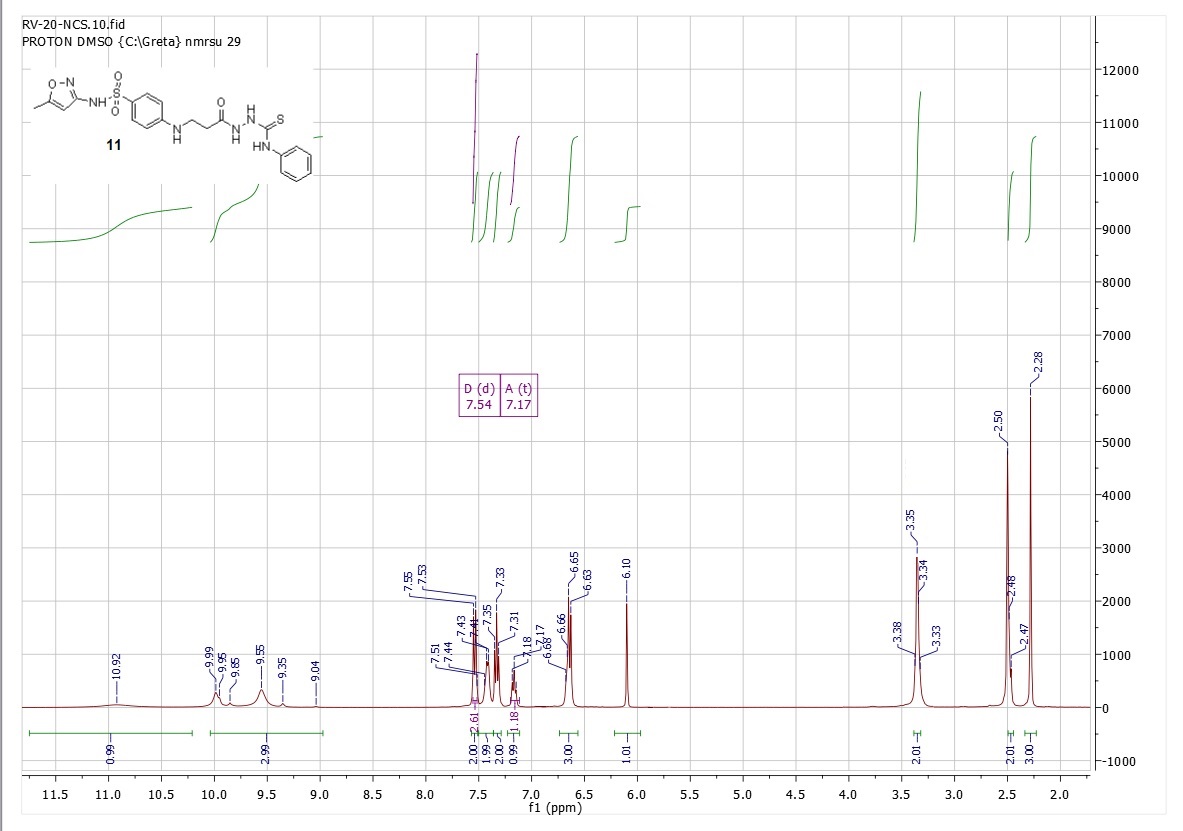


**S21 Fig.** ^1^H NMR of compound **11** at 400 MHz (DMSO-d_6_)

**
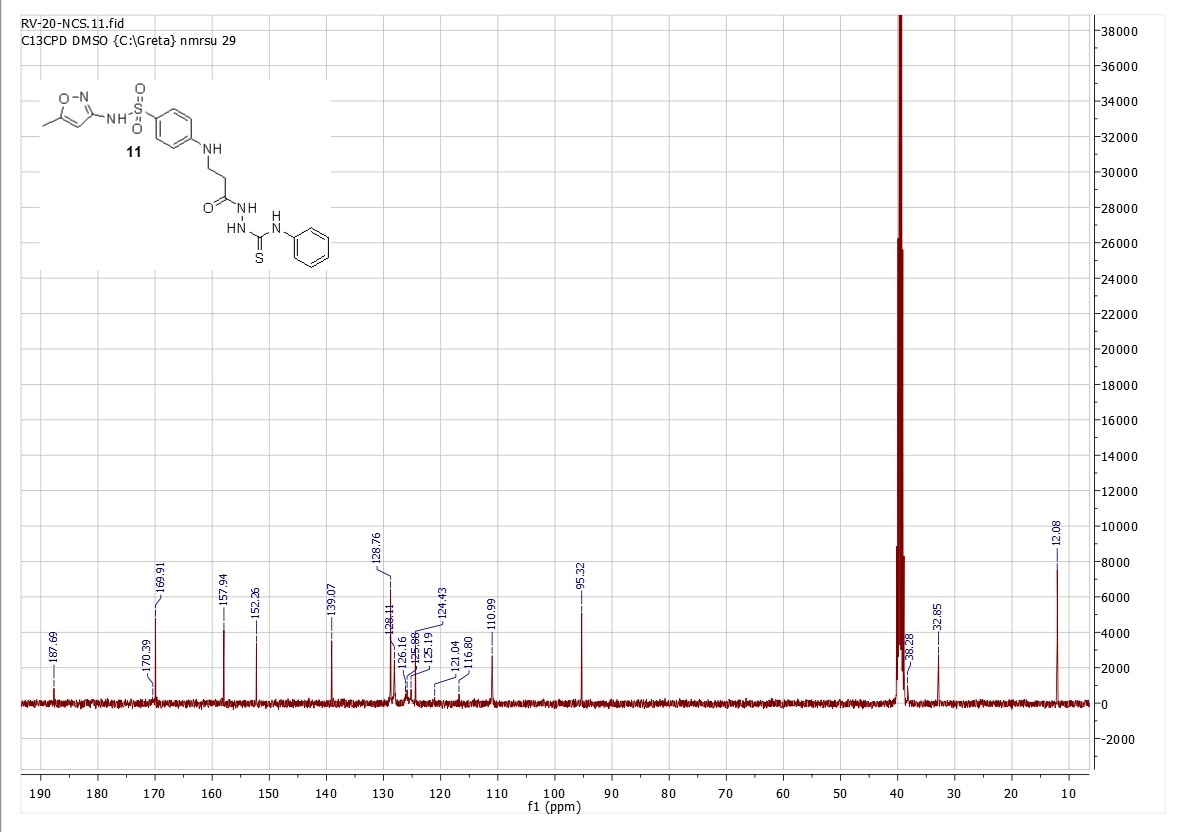
**

**S22 Fig.** ^13^C NMR of compound **11** at 101 MHz (DMSO-d_6_)

**
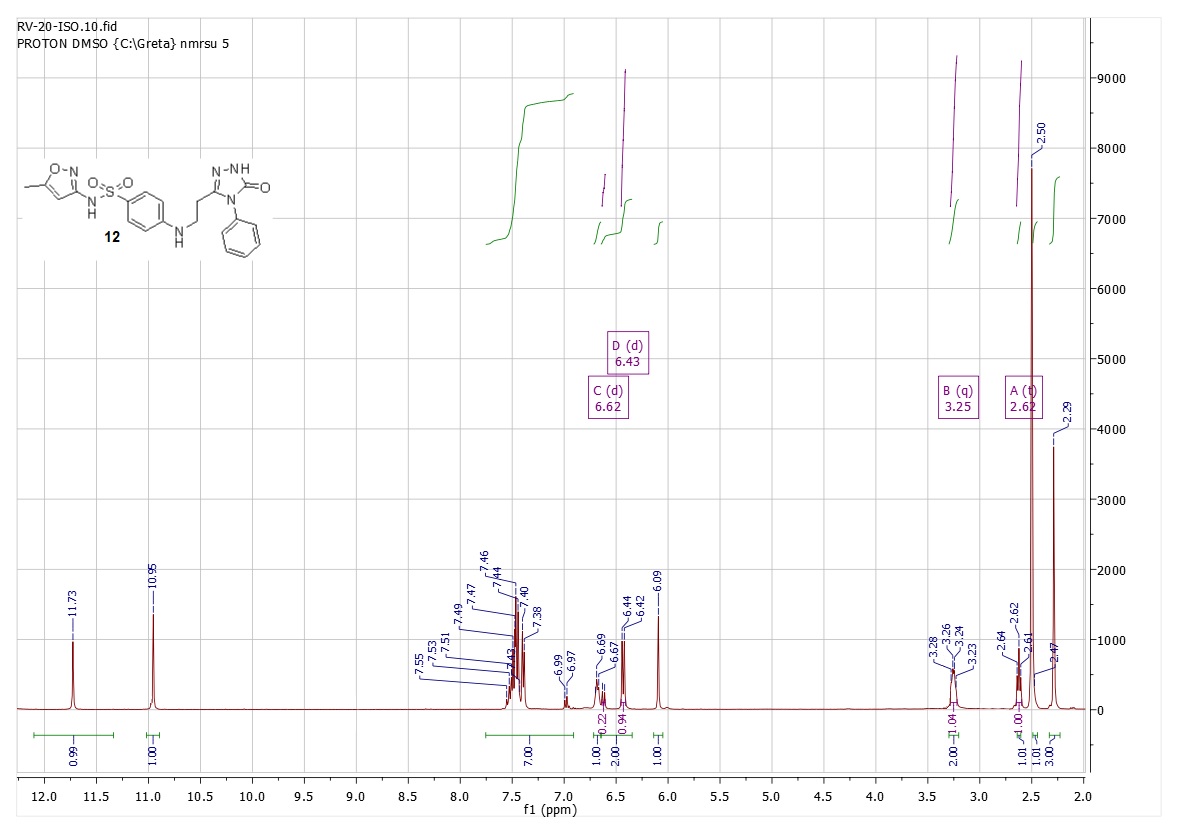
**

**S23 Fig.** ^1^H NMR of compound **12** at 400 MHz (DMSO-d_6_)

**
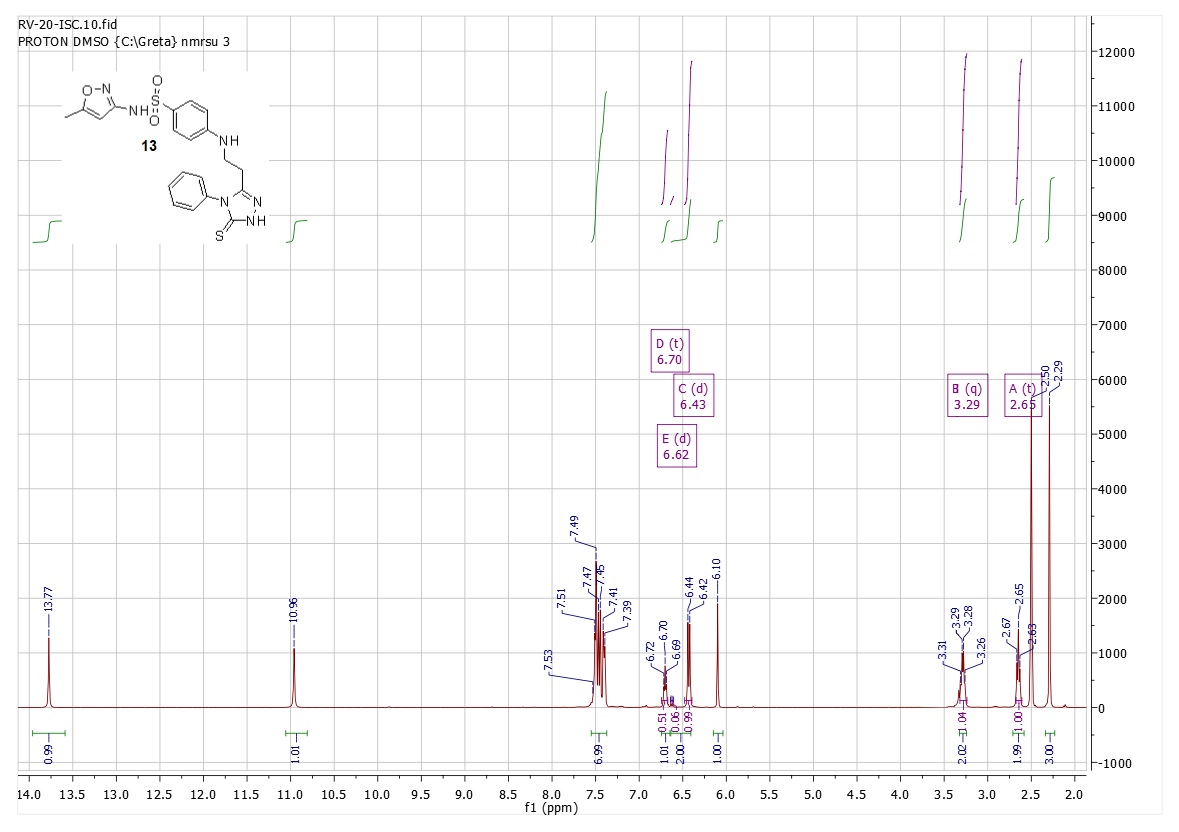
**

**S24 Fig.** ^1^H NMR of compound **13** at 400 MHz (DMSO-d_6_)

**
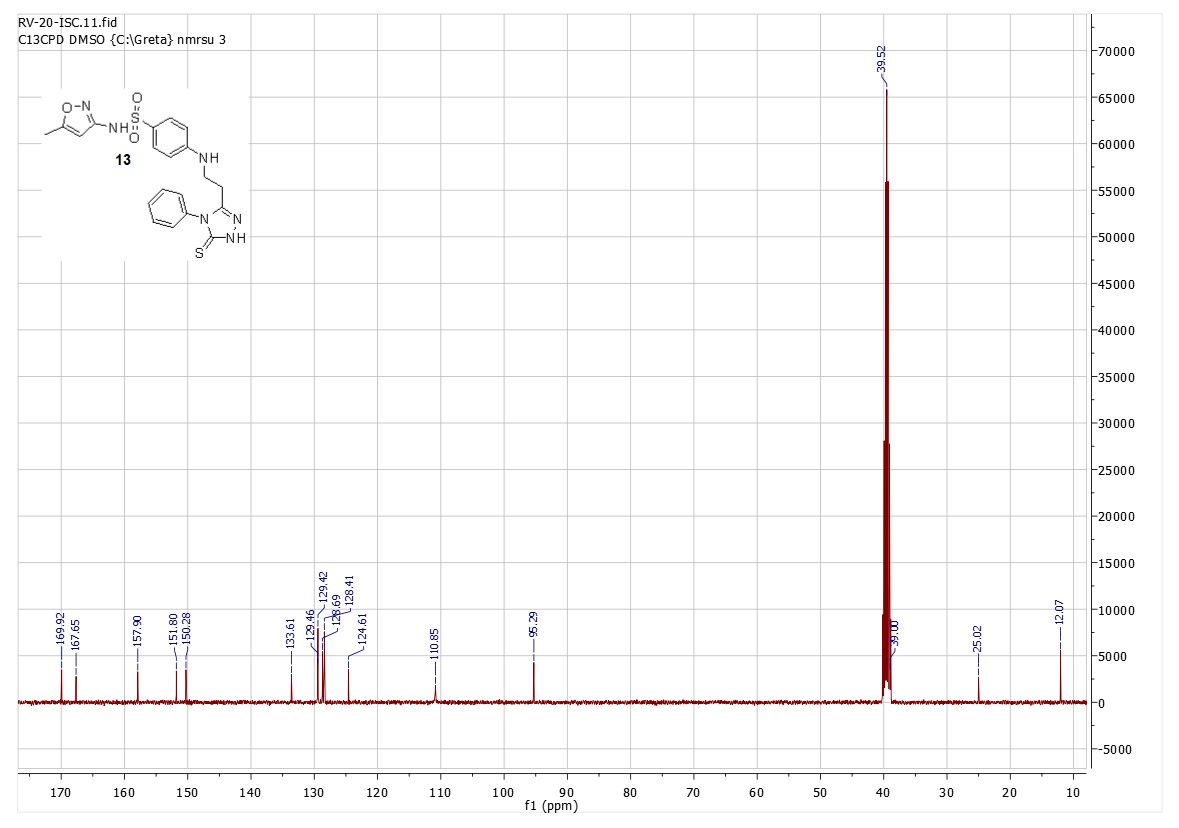
**

**S25 Fig.** ^13^C NMR of compound **13** at 101 MHz (DMSO-d_6_)

**
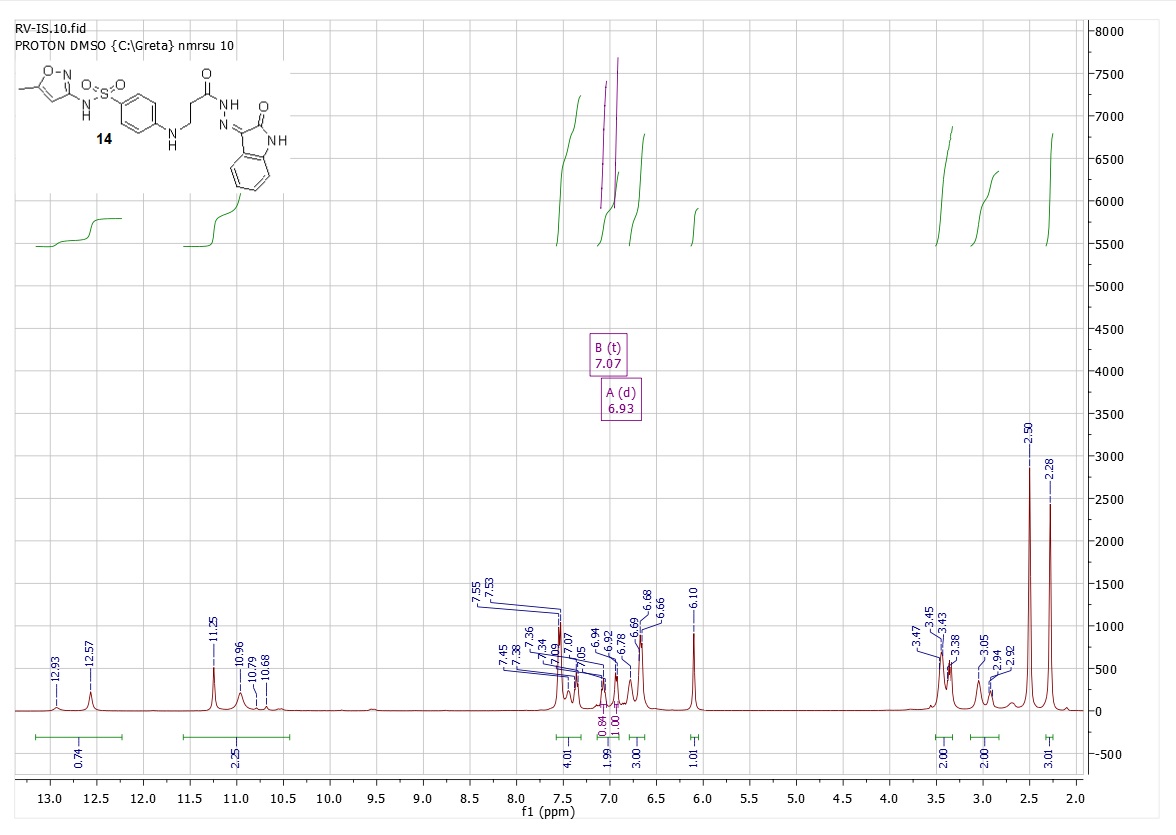
**

**S26 Fig.** ^1^H NMR of compound **14** at 400 MHz (DMSO-d_6_)

**
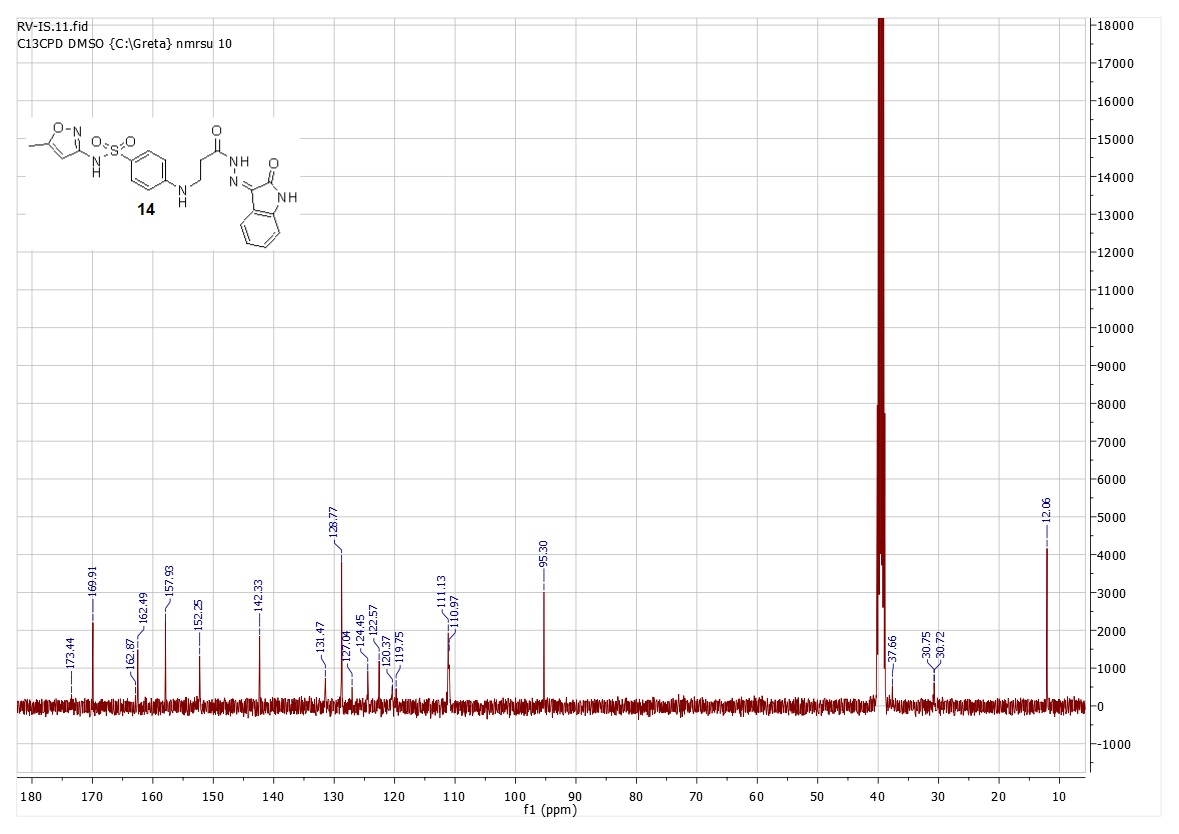
**

**S27 Fig.** ^13^C NMR of compound **14** at 101 MHz (DMSO-d_6_)


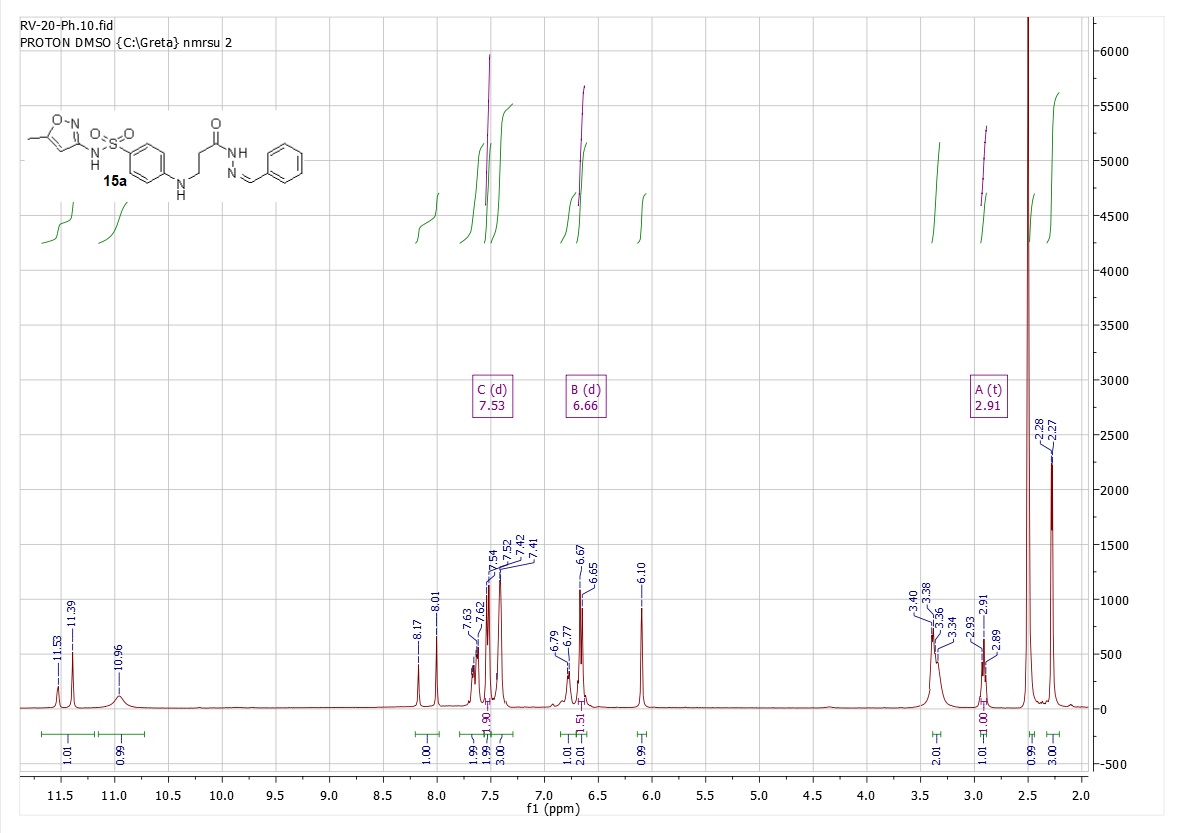


**S28 Fig.** ^1^H NMR of compound **15a** at 400 MHz (DMSO-d_6_)


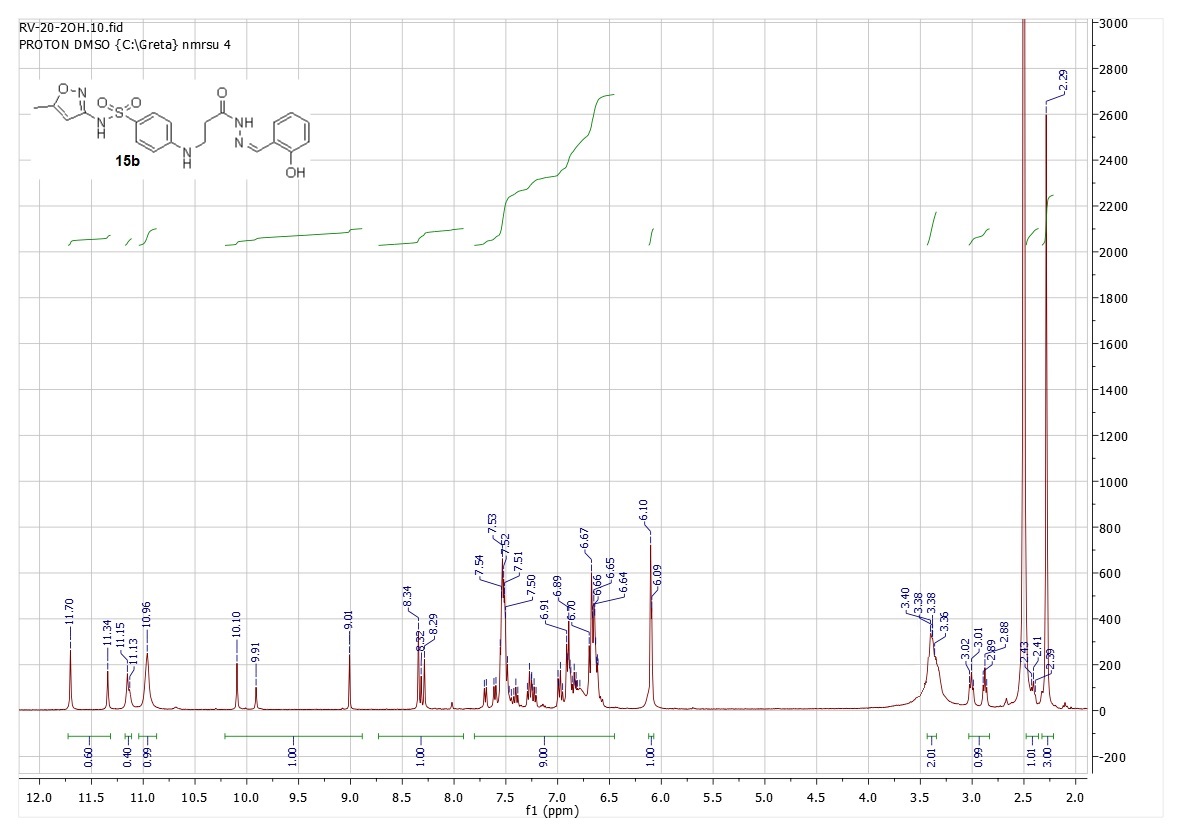


**S29 Fig.** ^1^H NMR of compound **15b** at 400 MHz (DMSO-d_6_)


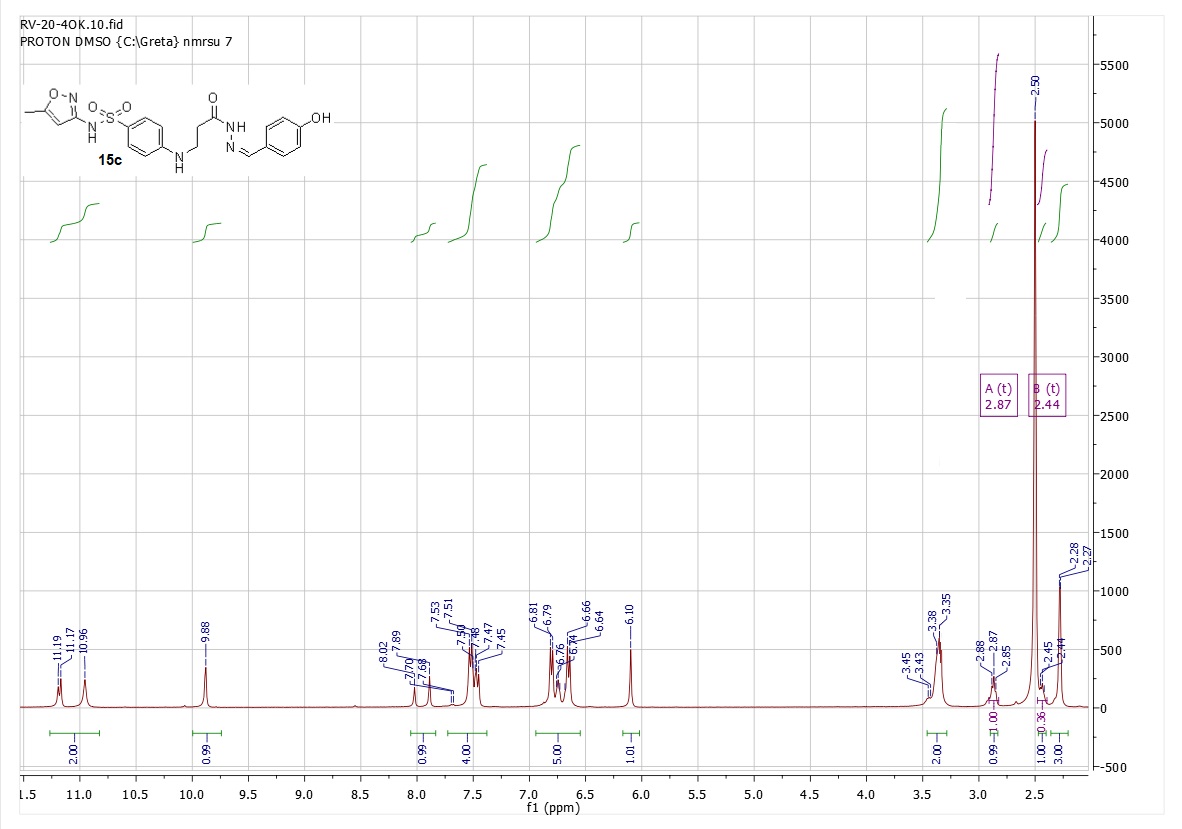


**S30 Fig.** ^1^H NMR of compound **15c** at 400 MHz (DMSO-d_6_)


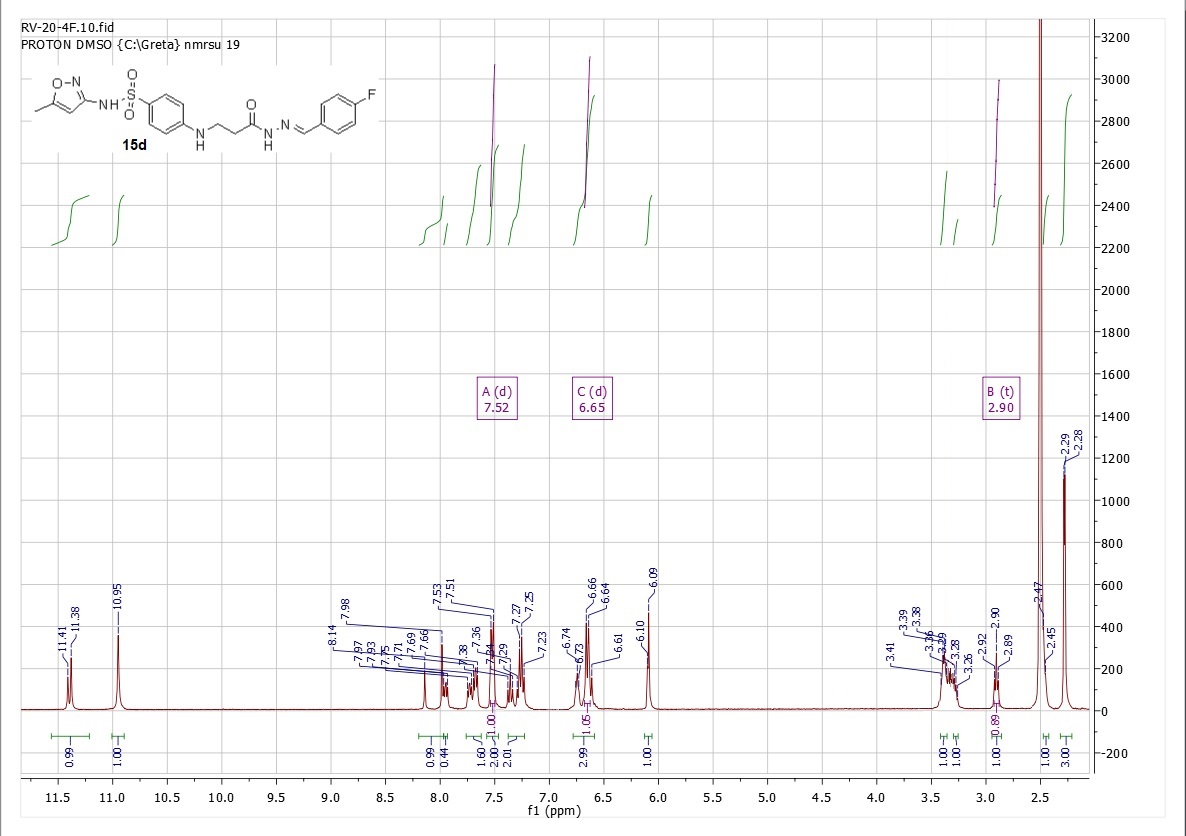


**S31 Fig.** ^1^H NMR of compound **15d** at 400 MHz (DMSO-d_6_)


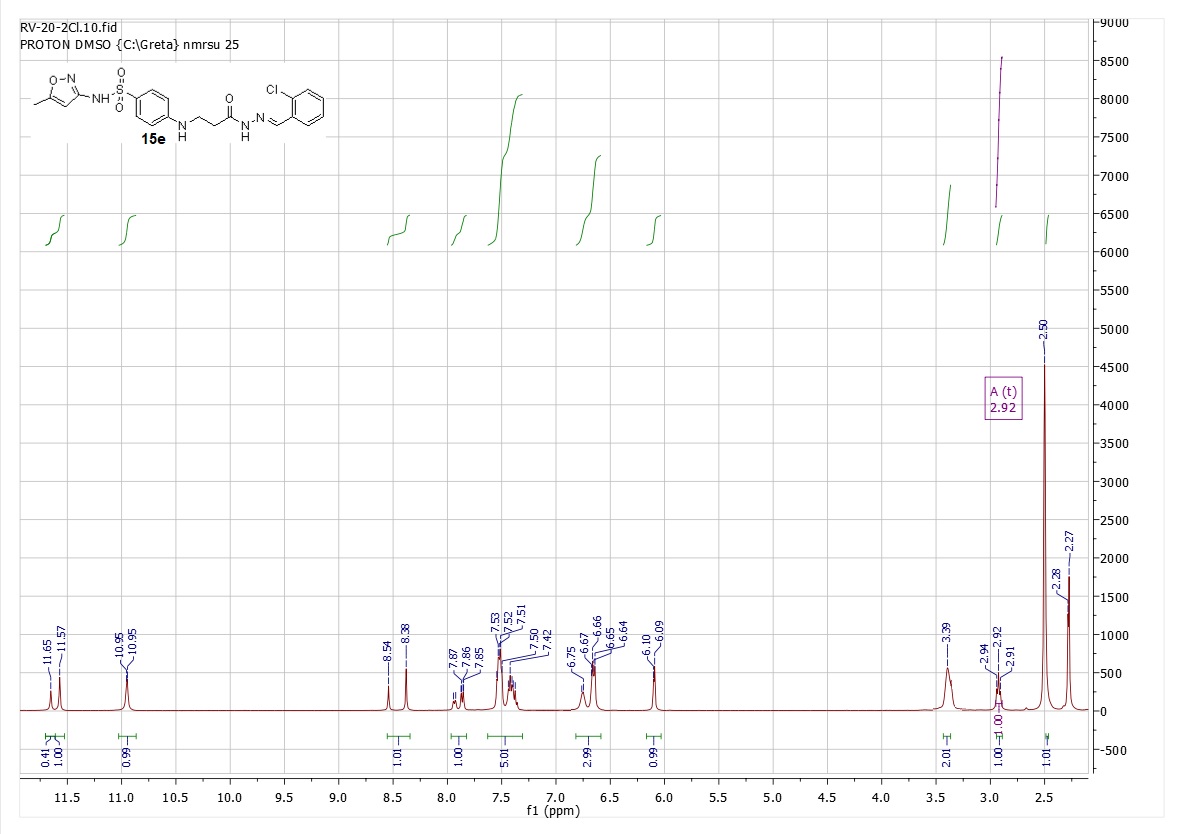


**S32 Fig.** ^1^H NMR of compound **15e** at 400 MHz (DMSO-d_6_)


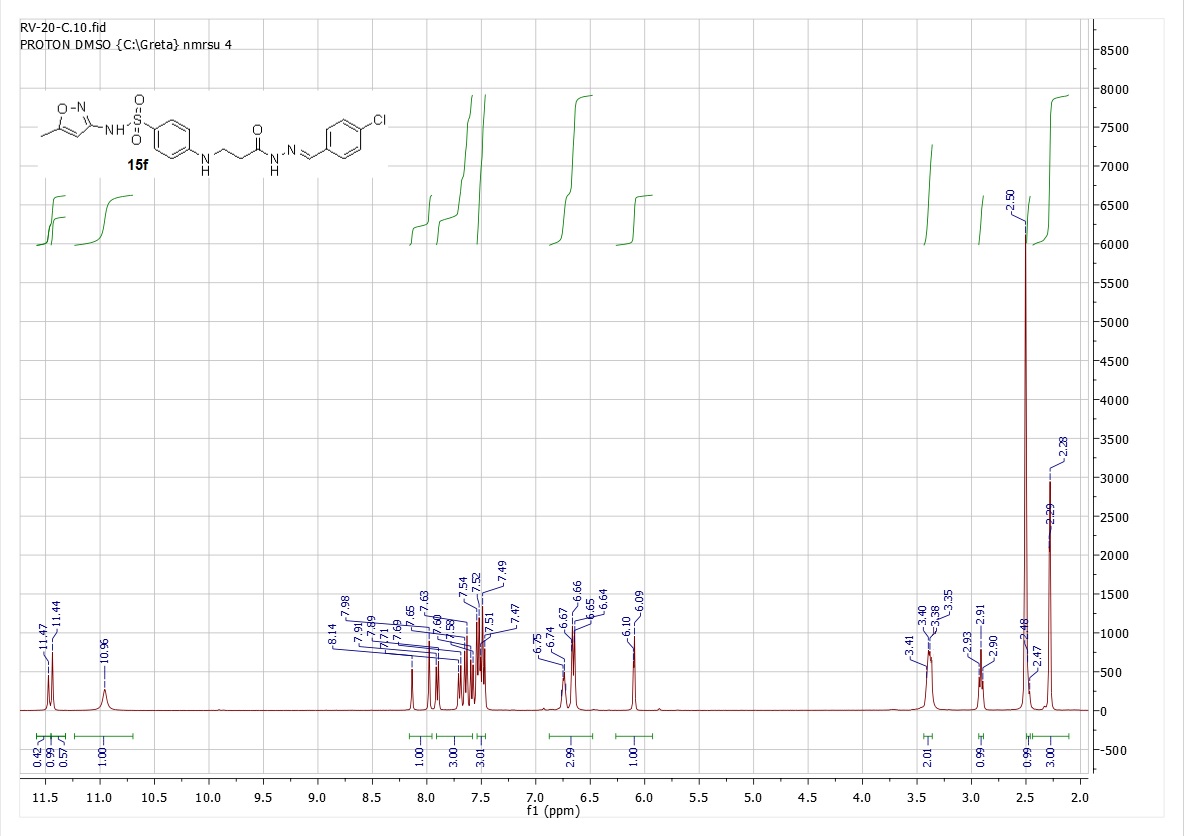


**S33 Fig.** ^1^H NMR of compound **15f** at 400 MHz (DMSO-d_6_)


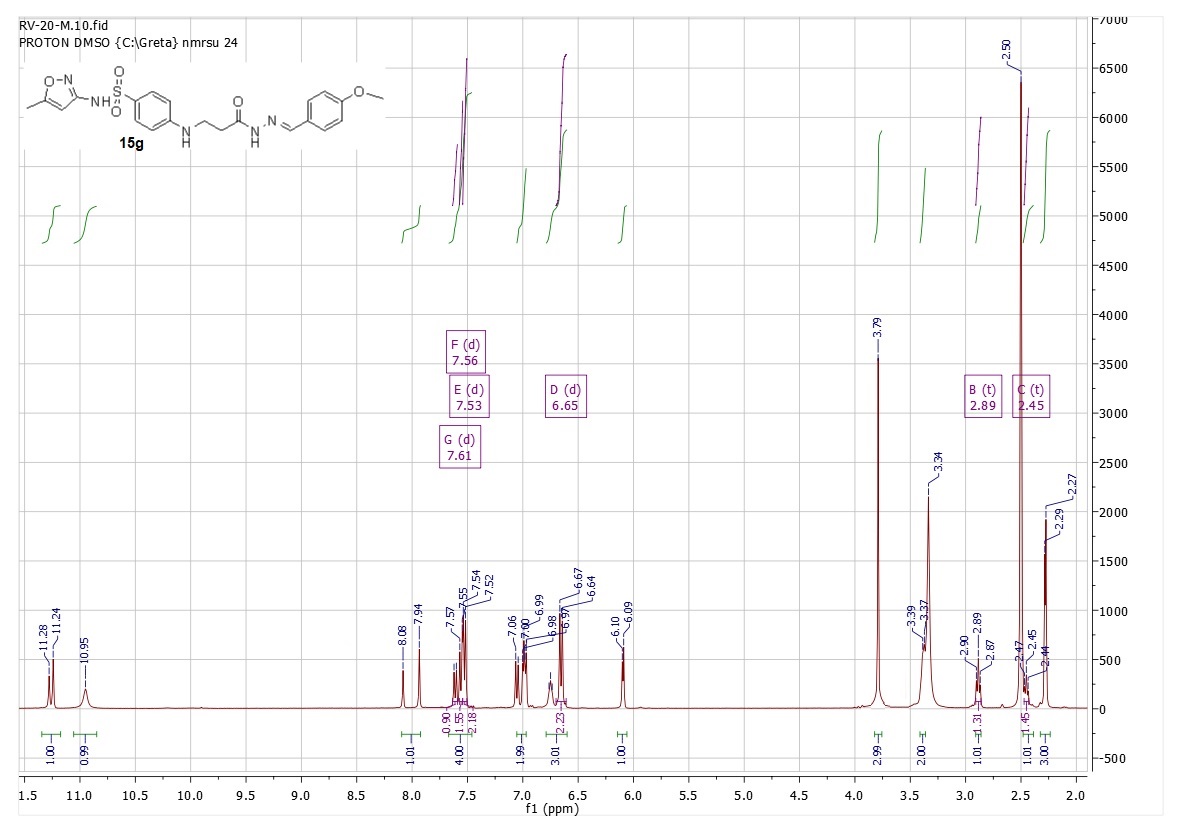


**S34 Fig.** ^1^H NMR of compound **15g** at 400 MHz (DMSO-d_6_)


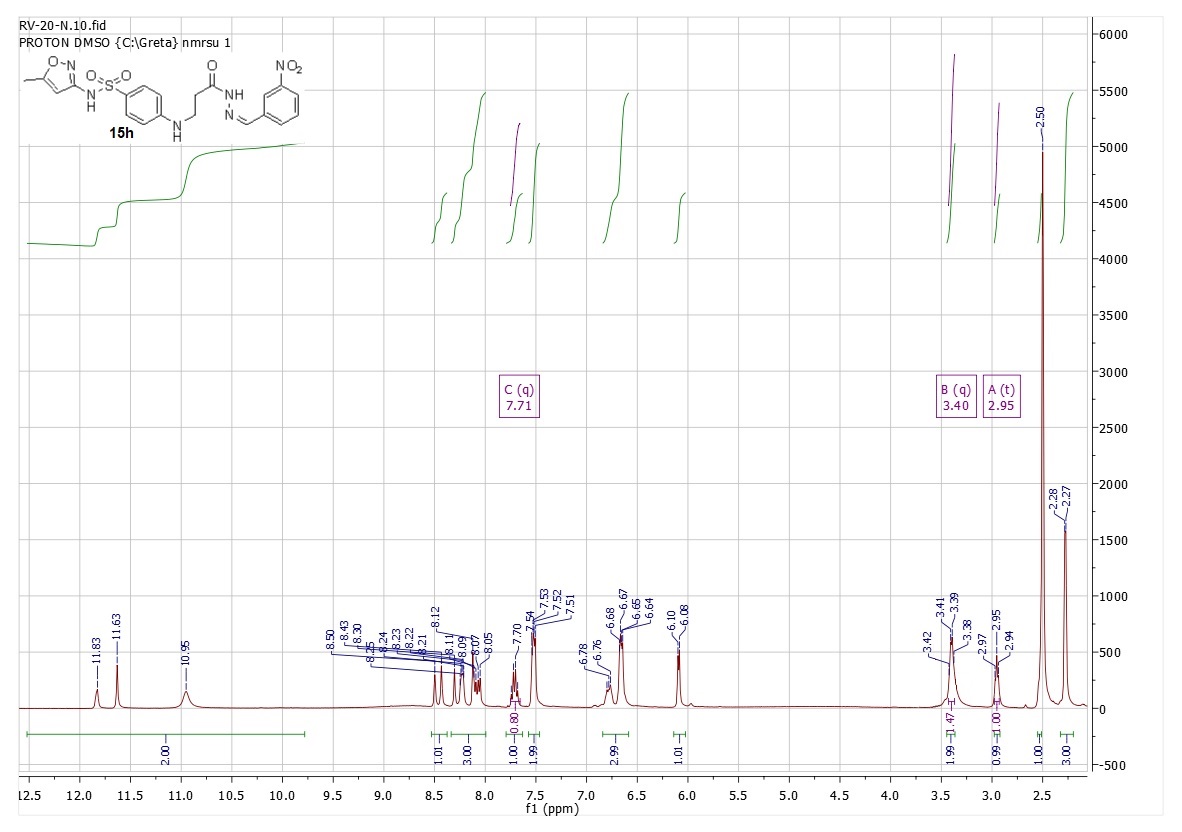


**S35 Fig.** ^1^H NMR of compound **15h** at 400 MHz (DMSO-d_6_)

**
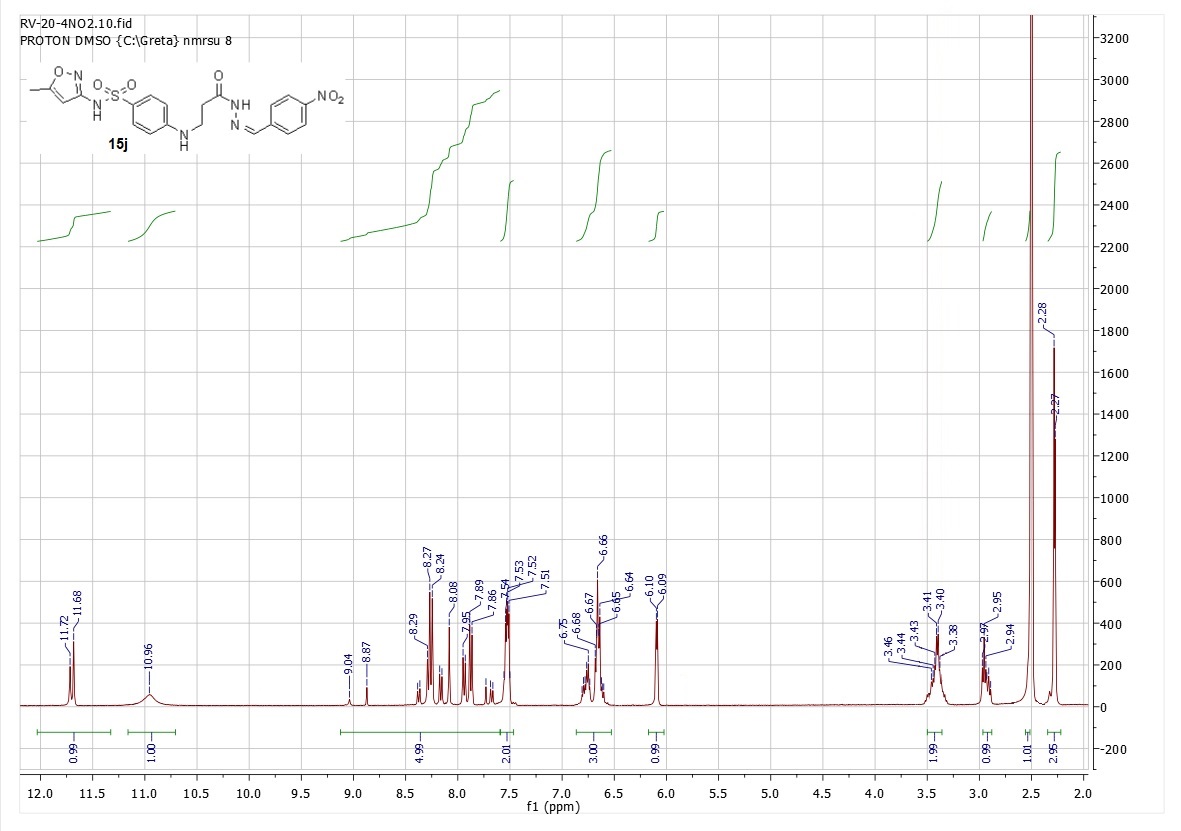
**

**S36 Fig.** ^1^H NMR of compound **15j** at 400 MHz (DMSO-d_6_)

**
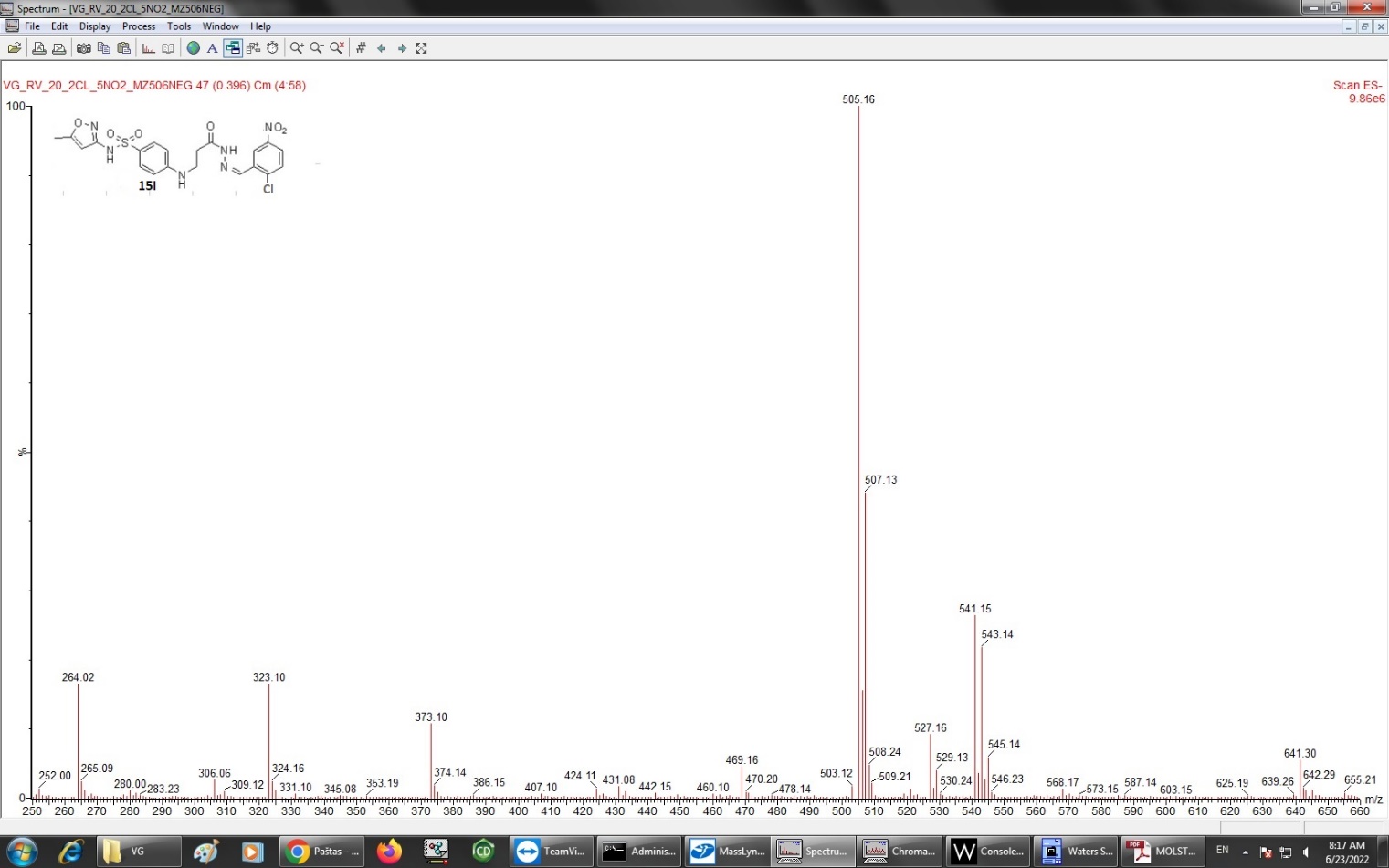
**

**S37 Fig.** Mass spectrum of compound **15i**


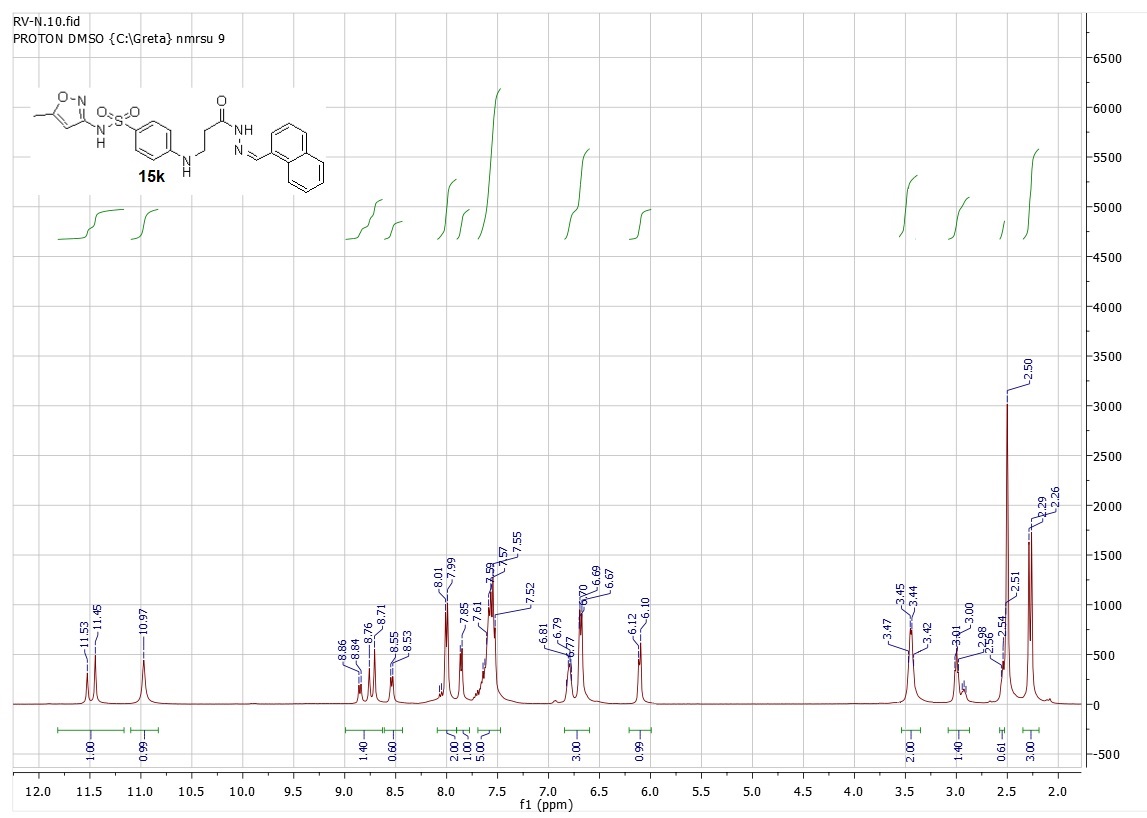


**S38 Fig.** ^1^H NMR of compound **15k** at 400 MHz (DMSO-d_6_)


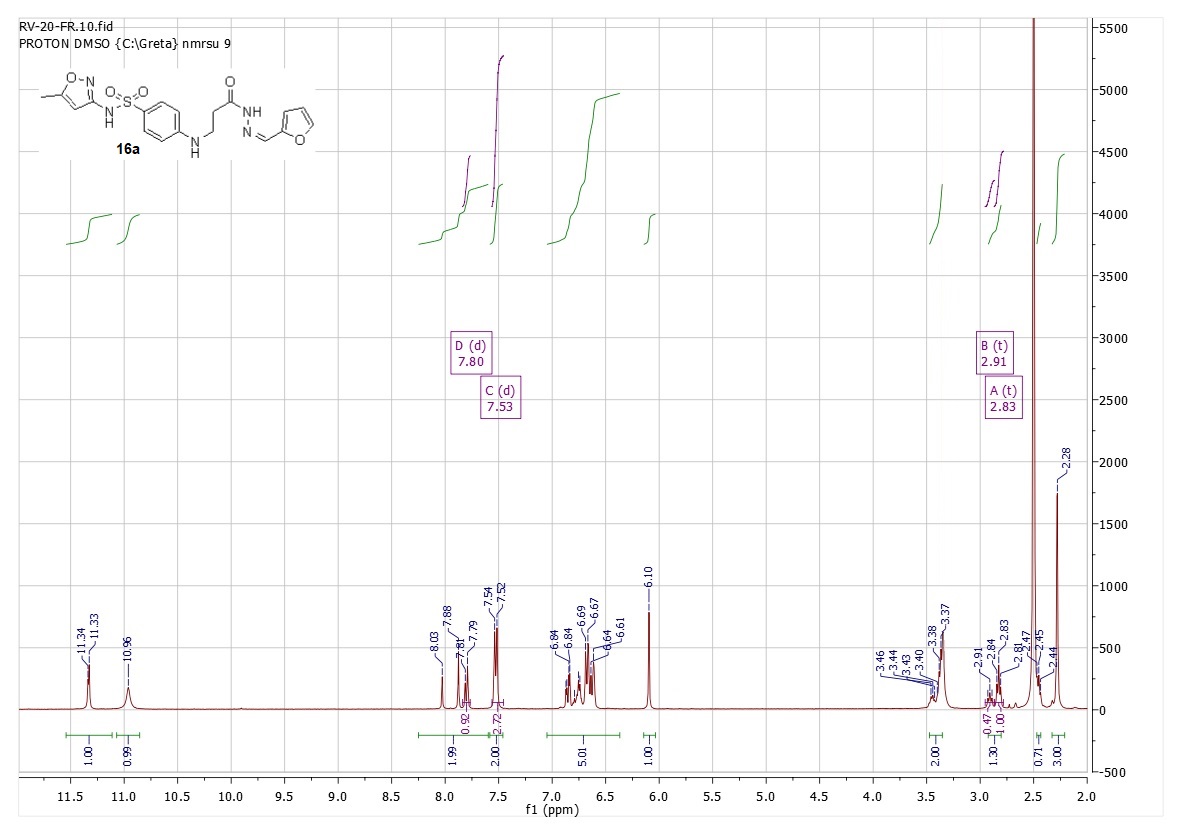


**S39 Fig.** ^1^H NMR of compound **16a** at 400 MHz (DMSO-d_6_)


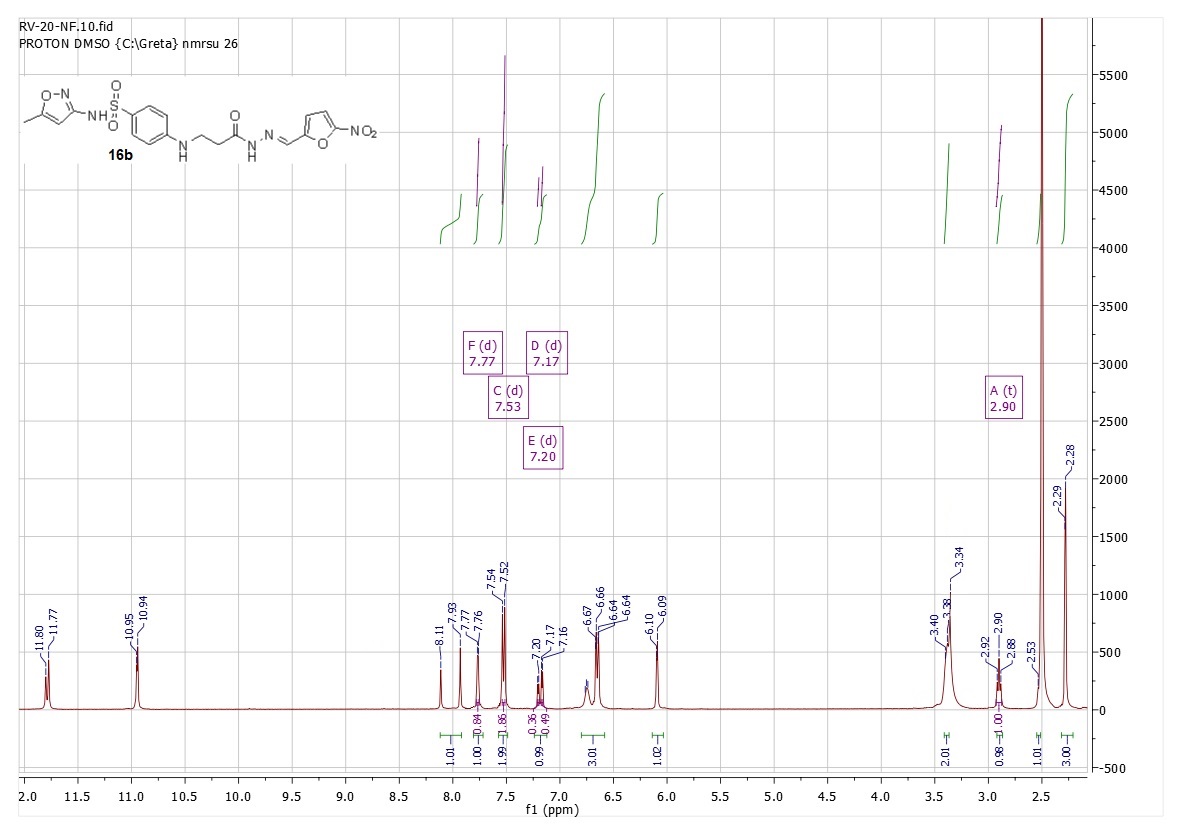


**S40 Fig.** ^1^H NMR of compound **16b** at 400 MHz (DMSO-d_6_)


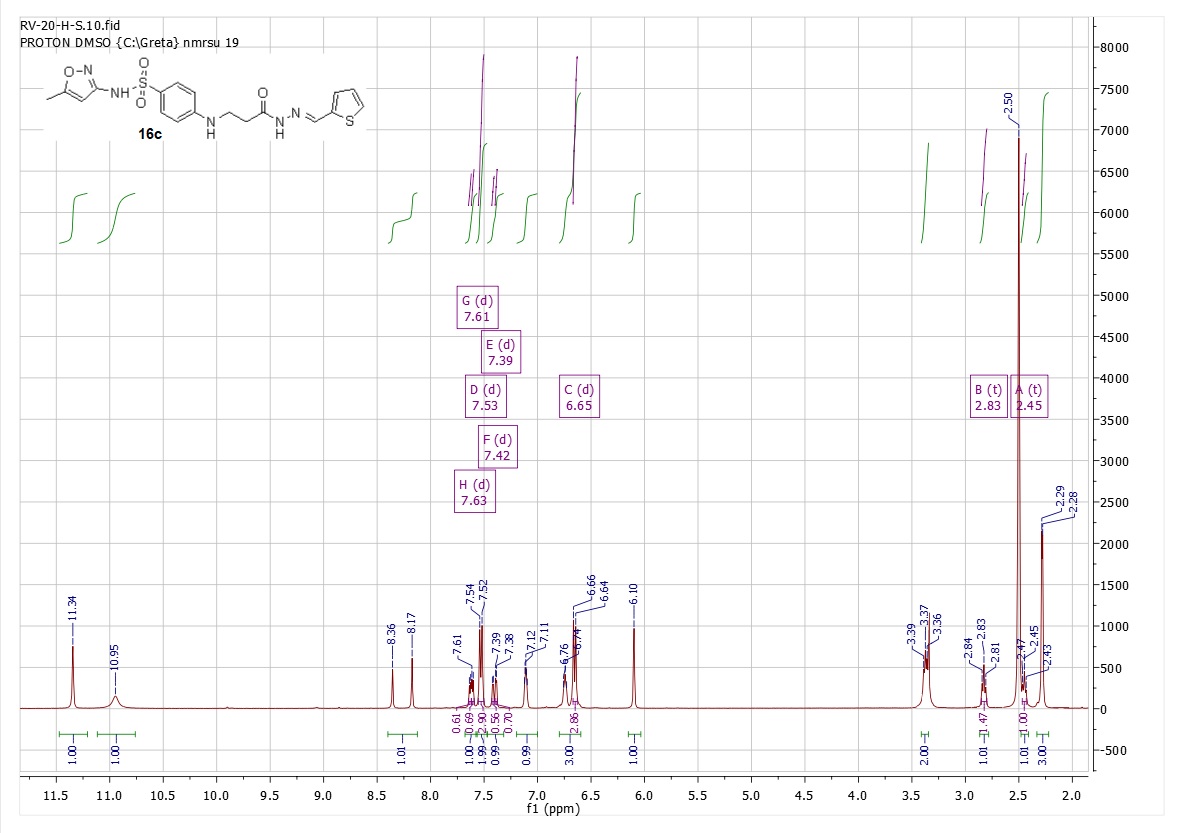


**S41 Fig.** ^1^H NMR of compound **16c** at 400 MHz (DMSO-d_6_)


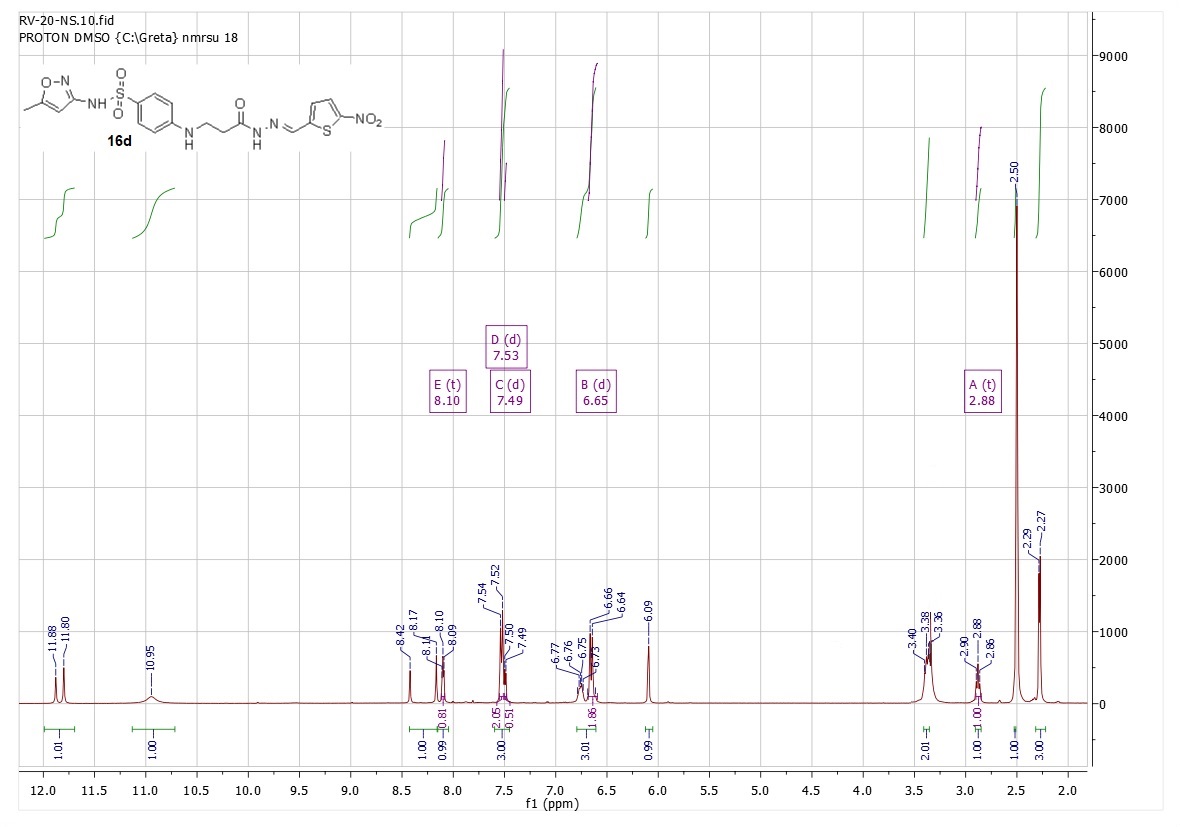


**S42 Fig.** ^1^H NMR of compound **16d** at 400 MHz (DMSO-d_6_)
